# SLC7A11 upregulation via AR and NEDD4L ubiquitination contributes to ferroptosis inhibition and enzalutamide resistance in castration-resistant prostate cancer

**DOI:** 10.1038/s41419-025-07809-4

**Published:** 2025-08-05

**Authors:** Min Tang, Luotong Xue, Bao Wang, Zhixin Jiang, Pu Li, Hengtao Bu, Chesong Zhao, Yurong Zhang, Shuaimei Liu, Xiyuan Chen, Bianjiang Liu, Xiaoxin Meng, Jie Li

**Affiliations:** 1 Department of Urology, The First Affiliated Hospital with Nanjing Medical University, Nanjing, Jiangsu China; 2 NHC Key Laboratory of Contraceptives Vigilance and Fertility Surveillance, Nanjing, Jiangsu China; 3 Jiangsu Provincial Medical Key Laboratory of Fertility Protection and Health Technology Assessment, Nanjing, Jiangsu China; 4 Jiangsu Health Development Research Center, Nanjing, Jiangsu China; 5 Department of Urology, Shuyang Hospital, Suqian, Jiangsu China

**Keywords:** Drug development, Cancer models

## Abstract

Advanced prostate cancer (PCa) is associated with a poor prognosis, particularly in patients who progress to castration-resistant prostate cancer (CRPC). The emergence of enzalutamide (Enz), a second-generation androgen receptor inhibitor, has effectively extended the median survival of these patients. However, drug resistance frequently develops during clinical use. Multiple studies have suggested that ferroptosis inducers can reverse cancer resistance to Enz, though the exact mechanisms remain unclear. In this study, we found that CRPC cells exhibit a concentration-dependent decrease in ferroptosis in response to Enz. This change is attributed to the upregulation of the protein level of SLC7A11. Protein stability assays and database analyses showed that SLC7A11 undergoes post-translational modification, likely connected to Enz-mediated downregulation of the SLC7A11-specific E3 ubiquitin ligase NEDD4L. This phenomenon was significantly reversed by the addition of erastin, a targeted inhibitor of the cystine/glutamate transport system Xc-. The data from both in vitro and in vivo experiments indicate that combining Enz with erastin significantly inhibits the proliferation of drug-resistant CRPC cells, thereby enhancing tumor suppression. These findings offer novel insights into targeting SLC7A11 with erastin as a potential strategy to overcome Enz resistance.

## Introduction

Prostate cancer (PCa) is the second most common solid tumor that threatens men’s health worldwide [1]. In 1941, Huggies first discovered that prostate cancer has androgen-dependent properties [2]. Reducing androgen levels or blocking androgen receptors (AR) can inhibit the progression of prostate cancer, making androgen deprivation therapy (ADT) a first-line treatment to prevent the progression and metastasis of prostate cancer [3]. However, although the initial efficacy of ADT exceeds 80% [4], most patients eventually progress to castration-resistant prostate cancer (CRPC). CRPC is characterized by persistent AR function and resistance to ADT. The median survival of CRPC patients is typically less than one year [5].

The introduction of second-generation AR antagonists enzalutamide (Enz), has provided hope to CRPC patients, significantly extending their median survival to 32.4 months [6]. Enz is a potent androgen receptor signaling inhibitor with no agonistic activity. It blocks the nuclear translocation of activated AR, preventing their localization to androgen response elements, co-activator recruitment, and ultimately inhibiting the proliferation of CRPC cells [5]. However, soon after its clinical use, new challenges arose: ~42% of CRPC patients do not respond to Enz, and even among those who do, resistance typically develops within 11.2 months [7]. Current studies suggest that resistance to Enz in CRPC may be linked to the development of AR splicing variants [8], AR mutations or deletion [9], and the increased AR gene transcriptional activity [10]. Nevertheless, the exact mechanisms remain unclear, and effective intervention targets to reverse Enz resistance are lacking. Thus, it is crucial to explore the underlying mechanisms in greater detail.

Ferroptosis, a form of regulatory cell death characterized by excessive intracellular lipid peroxide accumulation, is distinct from necrosis, apoptosis, and autophagy. It is marked by increased cytoplasmic and mitochondrial reactive oxygen species (ROS), mitochondrial shrinkage, increased membrane density, reduced or absent mitochondrial cristae, and ruptured outer membrane [11]. Ferroptosis has been implicated in various pathological states, including degenerative diseases like Alzheimer’s and Parkinson’s disease, as well as tumor-related cell death [12, 13]. Inducing ferroptosis is considered an important approach to inhibit tumor cell growth. Research into ferroptosis has shown that it can be triggered by certain small molecules or drugs. For example, erastin, a specific ferroptosis inducer, significantly enhances the anticancer activity of cytarabine and doxorubicin, first-line chemotherapy agents, in HL60 cells [14]. Inducing ferroptosis has been shown to overcome drug resistance in acute myeloid leukemic cells [15]. A major regulatory network for ferroptosis has been established, and it is widely accepted that disrupting signaling pathways maintaining intracellular redox balance leads to ferroptosis. The glutamate/cystine antiporter system Xc- and glutathione peroxidase GPX4 are considered key regulatory mechanisms in ferroptosis [16, 17].

In our study, we found that adding a specific concentration of Enz to CRPC cell lines upregulates SLC7A11 protein expression and inhibits ferroptosis. When erastin, an SLC7A11 inhibitor, was added, the sensitivity of CRPC cell lines to Enz increased. This suggests that SLC7A11 may play a role in the development of Enz resistance, and we are investigating the molecular mechanisms underlying this process.

## Materials and methods

### Cell culture and construction drug-resistant cell lines

The cells used in the experiment were prostate cancer cells C4-2 and 22RV1, both CRPC cell lines, purchased from the Chinese Academy of Sciences (Shanghai, China). The cell culture medium was RPMI-1640 (Gibco, USA), supplemented with 10% fetal bovine serum (FBS, Gibco) and 1% penicillin-streptomycin (Beyotime, Shanghai, China). All cell lines were cultured in a humidified incubator with 5% (v/v) CO_2_ at 37 °C.

To construct drug-resistant cell lines, the C4-2 and 22RV1 were cultured in Enz at one-tenth of its IC50 concentration for 1–2 weeks. The medium was changed regularly during culture. The morphology of the cells was observed and recorded every 2–3 passages. If there are significant changes in cell morphology or high level of cell death, the drug was withdrawn after 24–48 h of treatment, and fresh medium was provided to restore the cells for 24 h before re-administering the drug. If the cell were in good condition, the drug concentration was gradually increased every 2–3 passages. The IC50 was tested at least once a month, and the final dosing concentration used in this study was 40 μM. The drug resistance mechanism hypothesized and validated in this study is as follows: Enzalutamide stabilizes SLC7A11 by inhibiting its ubiquitination mediated by NEDD4L, thereby enhancing cystine uptake and promoting glutathione (GSH) biosynthesis. This, in turn, reduces susceptibility to ferroptosis and ultimately facilitates the development of enzalutamide resistance in prostate cancer cells.

### Determination of glutathione content (microplate-based methods)

Briefly, cells were washed twice with PBS, digested with trypsin (Genview, Beijing, China), and collected into 1.5 ml Eppendorf tubes. After centrifugation and resuspension, the cells were subjected to ultrasonic disruption followed by re-centrifugation, and the supernatant was collected for measurement. For tissue samples, the tissues were rinsed, blood was removed, and the samples were pulverized before centrifugation. After mixing the remaining reagents with the supernatant, 100 μl of detection solution was added to a 96-well plate, and absorbance was measured at 420 nm with a 1 cm light path using a spectrophotometer. The experiment was repeated three times, and the GSH content of the cells and tissues was calculated using a standard formula.

### Measurement of the labile iron pool (LIP)

Flow cytometry with the fluorescent iron sensor calcein acetoxymethyl ester (CA-AM) was used to detect the intracellular labile iron concentration. First, the adherent cells in each group were digested with trypsin and then made into cell suspensions. After being washed three times with PBS to remove the culture medium, a freshly—prepared CA—AM solution at a concentration of 10 μM was added, and the cells were incubated in an incubator at 37 °C for 30 min. Subsequently, the cells were washed twice with PBS to eliminate the excess CA-AM. The analysis was carried out using a Cytoflex LX flow cytometer (Beckman Coulter, USA) along with the software CyExpert. The experiment was repeated three times. The differences in cell fluorescence reflected the levels of the labile iron pool.

### Mitochondrial ROS analysis

Mitochondrial ROS production was measured using flow cytometry with the MitoSOX Red Mitochondrial Superoxide Indicator (YEASEN, Shanghai, China). Briefly, cells were digested with trypsin to prepare single-cell suspensions, washed three times with PBS, and incubated with freshly prepared 1 μM MitoSOX solution at 37 °C for 30 min. After incubation, cells were washed twice with PBS to remove excess MitoSOX and analyzed using a Cytoflex LX flow cytometer (Beckman Coulter, USA) with CyExpert software. Differences in cellular fluorescence intensity reflected variations in mitochondrial ROS levels. Each experiment was repeated three times.

### Mitochondrial membrane potential determination

Mitochondrial membrane potential was assessed using the JC-1 fluorescent probe. JC-1 forms red fluorescent aggregates in mitochondria with intact membrane potential (high MMP). Depolarized MMP (low MMP) causes JC-1 to remain in monomeric form, emitting green fluorescence. The red/green fluorescence ratio was quantified to assess MMP integrity. The dye buffer provided with the kit was diluted (5× to 1×) and kept on ice. After the cells were incubated in a 37 °C incubator, the JC-1 staining solution was discarded, and the cells were washed twice with the diluted dye buffer. Two mL of fresh cell culture solution was added, and the cells were observed under a fluorescence stereomicroscope (THUNDER Imager Live Cell).

### EDU assay

The EDU assay is based on the incorporation of the thymidine analog EdU (5-ethynyl-2′-deoxyuridine) during DNA synthesis, followed by a Click reaction to label EdU with a fluorescent dye, allowing the detection of cell proliferation. After 24 h of cell treatment, 30,000 PCa cells/well were seeded in the 24-well plate, and the EDU assay was performed after overnight incubation. According to the protocol, the cell supernatant was removed, and the Click reaction solution was prepared. Fluorescence detection was performed, and inverted fluorescence microscopy was used for observation. DAPI staining was observed in the blue channel, while Azide 555 was observed in the RFP channel (red). Fields with uniform cell distribution were photographed, and the percentage of red-labeled cells was calculated.

### Western blot analysis

Clean the glass plates and prepare the necessary materials for gel preparation. Equal amounts of protein were separated by SDS-PAGE and transferred onto PVDF membranes. Membranes were blocked with 5% non-fat milk in TBS-T for 2 h at room temperature, followed by overnight incubation with primary antibodies at 4 °C. The dilution ratios for primary antibodies used in this study were as follows: AR (1:5000), SLC7A11 (1:1000), PRDX6 (1:2000), GPX4 (1:1000), GAPDH (1:10,000), and Tubulin (1:5000). After washing with TBS-T, membranes were incubated with HRP-conjugated secondary antibodies for 2 h at room temperature. For secondary antibodies, the dilution ratios were as follows: rabbit-derived antibodies (1:5000) and mouse-derived antibodies (1:5000). The signal was detected using enhanced chemiluminescence (ECL) and imaged on a gel documentation system.

### RNA extraction and qRT-PCR analysis

Cells were lysed in TRIzol reagent (1 mL per 10^6^ cells), followed by chloroform addition (0.2 mL per mL of TRIzol). Samples were shaken vigorously and incubated at room temperature for 3 min. After centrifugation at 12,000 × *g* for 15 min at 4 °C, the aqueous phase was transferred to a fresh tube, and RNA was precipitated with isopropanol (0.5 mL per mL of TRIzol). The RNA pellet was washed with 75% ethanol, air-dried, and dissolved in RNase-free water. RNA concentration and purity were assessed by spectrophotometry. Total RNA was reverse transcribed using a cDNA synthesis kit, and qRT-PCR was performed with SYBR Green Master Mix on a real-time PCR system. GAPDH or β-actin was used as an internal control. The 2^−ΔΔCt^ method was used to calculate relative gene expression.

### Co-Immunoprecipitation (Co-IP)

Cells were lysed in ice-cold RIPA buffer (50 mM Tris-HCl pH 7.4, 150 mM NaCl, 1% NP-40, 0.5% sodium deoxycholate, 0.1% SDS) supplemented with protease inhibitor cocktail (Roche) and phosphatase inhibitors (PhosSTOP, Roche). Lysates were clarified by centrifugation at 12,000 × *g* for 15 min at 4 °C. Supernatants were pre-cleared with protein G magnetic beads (Dynabeads, Invitrogen) for 1 h at 4 °C. Target proteins were immunoprecipitated by incubating lysates with primary antibodies (NEDD4L and SLC7A11) overnight at 4 °C, followed by addition of protein G beads for 2 h. Beads were washed 4 times with ice-cold RIPA buffer and eluted with 2× SDS-PAGE loading buffer at 95 °C for 5 min. Eluates were analyzed by Western blot using corresponding antibodies.

### Xenotransplantation model in vivo studies

Twenty healthy male mice (5 weeks old) were randomly divided into 4 groups, with 5 animals per group for subcutaneous tumor formation. To induce tumors, 6 × 10^6^ C4-2 cells were suspended in 200 μL of 0.9% NaCl solution and injected subcutaneously into the right armpit of the mice. Tumors were allowed to grow until they reached 100 mm³, after which the mice were treated with either Enz (10 mg/kg/day) or Erastin(20 mg/kg/day) orally for 4 weeks. Tumor size was measured every 4 days using the formula [length (mm) × width^2^(mm)] × 0.5. At the end of the experiment, mice were euthanized by carbon dioxide asphyxiation, and tumor tissues were removed for measurement of ferroptosis-related indicators.

### Database

In our study, we utilized the E3 ubiquitin ligase prediction database UbiBroswer version 1.0 (http://ubibrowser.bio-it.cn/ubibrowser/). We also used transcription factors databases, including hTFtarget (http://bioinfo.life.hust.edu.cn/hTFtarget#!) and CHEA transcription (https://maayanlab.cloud/Harmonizome/dataset/CHEA+Transcription+Factor+Targets), to explore molecular pathways.

### Statistics

Experimental data were analyzed using GraphPad Prism. The Kolmogorov-Smirnov test was used to assess the normality of the data distribution (*p* > 0.05 indicated a normal distribution). For multiple comparisons, analysis of variance (ANOVA) was used, with *p* ≤ 0.05 considered statistically significant.

## Results

### Enzalutamide downregulates ferroptosis indices in CRPC prostate cancer cell line

**Fig. 1:**
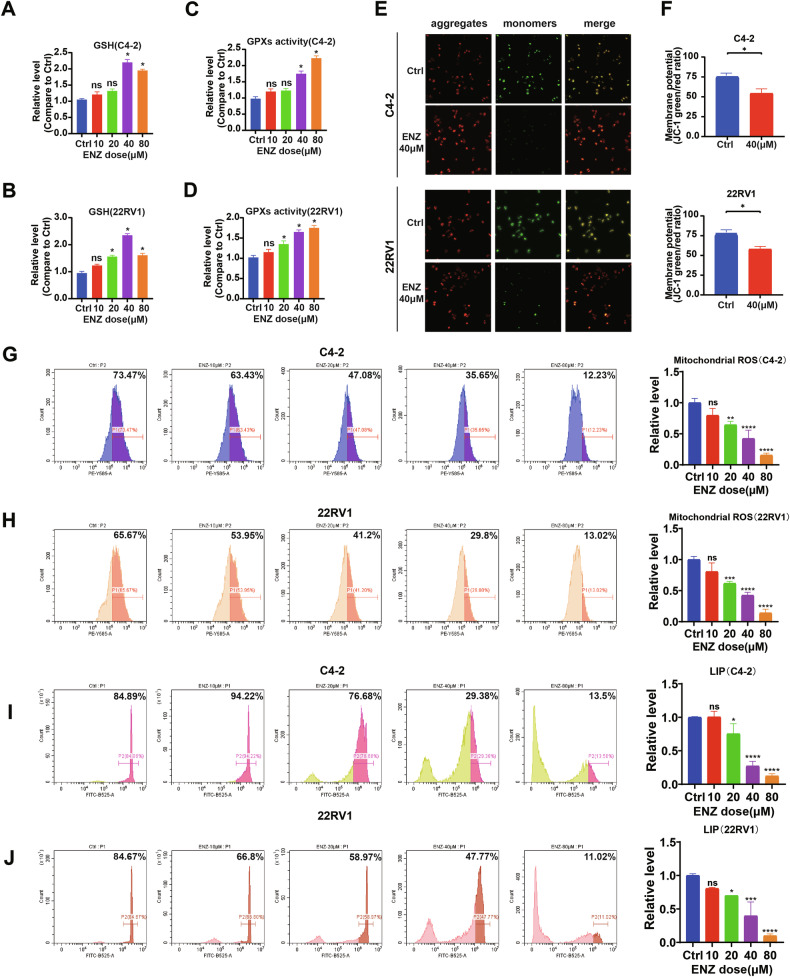
Enzalutamide downregulates ferroptosis indices in CRPC prostate cancer cell line. **A–D** Measurement of glutathione and peroxidase activity in CRPC cells under various concentrations of enzalutamide. GSH levels in C4-2 (**A**) and 22RV1 (**B**) cell lines exhibited a concentration-dependent increase, peaking at 40 μM. **C**, **D** Peroxidase activity in C4-2 cells significantly increased at 40 μM and further elevated at 80 μM (**C**). In 22RV1, significant increases were observed at 20 μM and further increased at 40 μM and 80 μM (**D**). **E**, **F** JC-1 fluorescence changes in C4-2 and 22RV1 cells treated with 40 μM enzalutamide. Green fluorescence indicates MMP collapse (ferroptosis), while red fluorescence reflects preserved MMP. Concentration-dependent decreases in mitochondrial ROS levels were observed in both C4-2 (**G**) and 22RV1 cells (**H**). Concentration-dependent decreases in LIP levels were also observed in both C4-2 (**I**) and 22RV1 cells (**J**). ns not significant, *p < 0.05, **p < 0.01, ***p < 0.001, ****p < 0.0001.

First, in this study, we added different concentrations of enzalutamide (with DMSO as a drug carrier and control) to the cell culture supernatant for 24 h. The concentrations included 10 μM, 20 μM, 40 μM and 80 μM. The measured indices included GSH, total iron ions, peroxidase activity (GPXs activity), and mitochondrial membrane potential (JC-1). Preserved mitochondrial membrane potential (reflected by a higher red/green fluorescence ratio), elevated glutathione levels, and increased peroxidase activity are associated with reduced ferroptosis, whereas elevated total iron ions indicate ferroptosis induction. In the C4-2 and 22RV1 cell lines, GSH levels showed a dose-dependent increase that peaked at 40 μM (Fig. 1A, B). Next, we measured peroxidase activity in C4-2 cells, which increased significantly at 40 μM and further increased at 80 μM (Fig. 1C). In 22RV1, significant increases were observed at 20 μM, with further increases at 40 μM and 80 μM (Fig. 1D). Based on these results, we selected a 40 μM concentration of enzalutamide to further investigate mitochondrial membrane potential changes. The JC-1 fluorescence ratio (red/green) reflects mitochondrial membrane potential (MMP). Higher green fluorescence (monomeric JC-1) indicates MMP collapse and mitochondrial dysfunction, a hallmark of ferroptosis, while higher red fluorescence (aggregated JC-1) represents preserved MMP. After the addition of 40 μM enzalutamide, the proportion of green fluorescence decreased in both cell lines compared to the control group (Fig. 1E, F).

Subsequently, we used flow cytometry to measure mitochondrial reactive oxygen species (ROS) levels and Labile iron pool (LIP) levels in CRPC cells treated with different concentrations of enzalutamide. The analysis indicated that mitochondrial ROS levels and LIP levels decreased in a dose-dependent manner in both C4-2 and 22RV1 cells (Fig. 1G, J).

Taken together, these results indicate that enzalutamide treatment effectively reduces ferroptosis levels in CRPC cells.

### SLC7A11 regulates ferroptosis in CRPC cells in response to enzalutamide

**Fig. 2:**
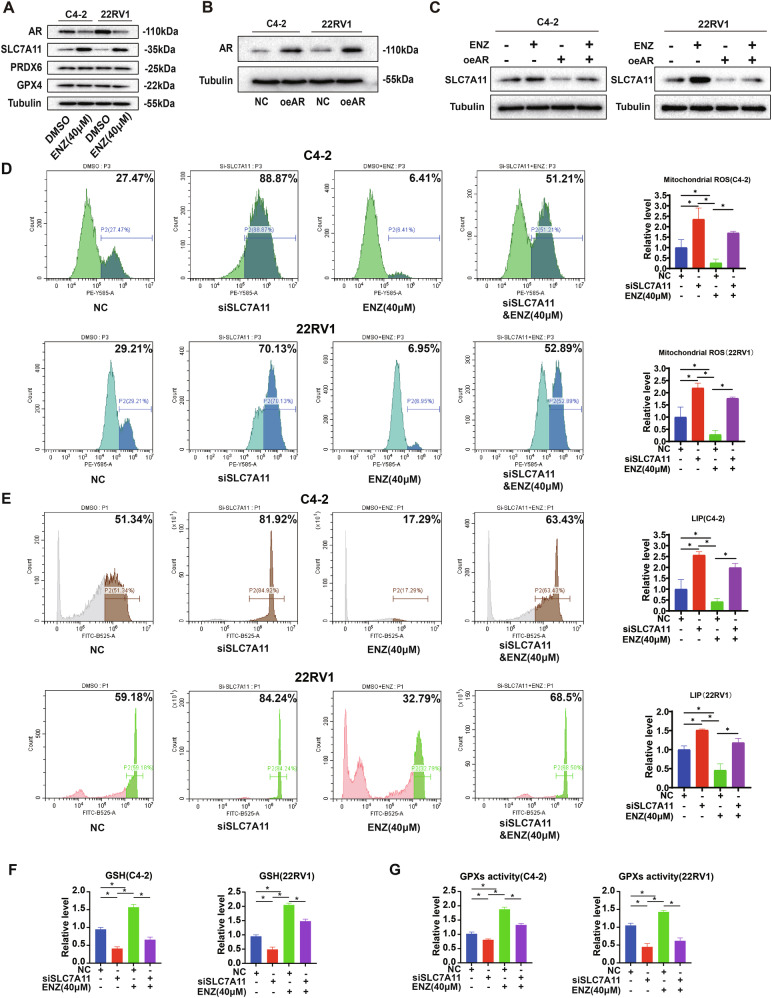
SLC7A11 regulates ferroptosis in CRPC cells in response to enzalutamide. **A–C** Enzalutamide regulates SLC7A11 protein expression via AR **A** Expression levels of ferroptosis regulatory proteins in C4-2 and 22RV1 cell lines after treatment with 40 μM enzalutamide. **B** Efficiency of AR overexpression in C4-2 and 22RV1 cell lines. **C** SLC7A11 protein expression levels in C4-2 and 22RV1 cells were evaluated under conditions of AR overexpression, enzalutamide (40 μM) treatment, or enzalutamide addition following AR overexpression. Flow cytometry was utilized to detect mitochondrial ROS levels and LIP levels in C4-2 and 22RV1 cells (**D**, **E**). Results indicated that knocking down SLC7A11 reversed the changes in mitochondrial ROS levels (**D**) and LIP levels (**E**) induced by enzalutamide in C4-2 and 22RV1 cells. **F** The increase in GSH levels caused by enzalutamide was reversed upon addition of enzalutamide following SLC7A11 knockdown in C4-2 and 22RV1 cells. The peroxidase activity exhibited a similar pattern (**G**). *p < 0.05, ns: not significant. NC refers to Negative Control, which is the control group transfected with empty vector siRNA.

We examined the protein expression levels of three key regulatory molecules of ferroptosis: GPX4, PRDX6, and SLC7A11, in response to enzalutamide. As observed, there were no significant changes in the protein expression levels of PRDX6 and GPX4 in the two CRPC cell lines treated with enzalutamide at 40 μM, while the expression of SLC7A11 was significantly up-regulated (Fig. 2A). We then explored the regulatory relationship between AR and SLC7A11. First, we constructed the CRPC cell line after AR overexpression and recorded the efficiency (Fig. 2B). Following AR overexpression, the protein level of SLC7A11 was down-regulated compared to control group (Fig. 2C). When enzalutamide was added, the expression level of SLC7A11 was up-regulated compared to the control group. However, the expression of SLC7A11 was down-regulated when enzalutamide was added after AR overexpression. This suggests that enzalutamide can regulate SLC7A11 protein expression through AR.

We then investigated the correlation between SLC7A11 and enzalutamide in ferroptosis levels. Groups were established based on the no-load plasmid control (NC) group, SLC7A11 knockdown group, enzalutamide (40 μM) group, and siSLC7A11 combined with enzalutamide (40 μM) group. Flow cytometry was used to detect mitochondrial ROS levels and LIP levels in C4-2 and 22RV1 cell lines (Fig. 2D, E). It was found that ROS levels and LIP levels increased when SLC7A11 expression was knocked down, but decreased when 40 μM enzalutamide was added. When SLC7A11 was knocked down before adding enzalutamide, mitochondrial ROS levels and LIP levels rose again (Fig. 2D, E). We also measured glutathione and peroxidase activity in the C4-2 cell line under the same conditions. Intracellular glutathione levels were down-regulated by SLC7A11 knockdown and increased with the addition of enzalutamide. The increase in GSH levels caused by enzalutamide was reversed when enzalutamide was added after SLC7A11 knockdown (Fig. 2F). Similar results were observed in 22RV1 (Fig. 2F). Peroxidase activity followed the same pattern in the same experimental group (Fig. 2G).

**Fig. 3:**
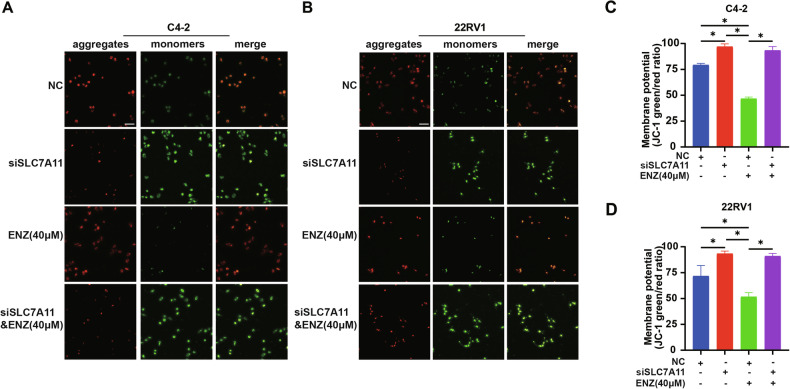
Knocking down SLC7A11 in CRPC cells reversed mitochondrial membrane potential changes induced by enzalutamide. **A–D** Following SLC7A11 knockdown in C4-2 cells, the proportion of green fluorescence was increased compared to the control, while the proportion decreased after the addition of enzalutamide (40 μM). **A–C** When SLC7A11 was knocked down prior to enzalutamide addition, the green fluorescence ratio significantly increased compared to enzalutamide treatment alone. **B–D** Similar results were obtained in 22RV1 cells. *p < 0.05, ns not significant.

The mitochondrial membrane potential JC-1 test also supported these results. We detected the proportion of green and red fluorescence of JC-1 in C4-2 cells (Fig. 3A, C). When SLC7A11 was knocked down, the proportion of green fluorescence increased compared to the control group, and the proportion of green fluorescence decreased after the addition of enzalutamide (40 μM). When SLC7A11 was knocked down before enzalutamide addition, the green fluorescence ratio increased significantly increased compared to enzalutamide alone. Similar results were obtained in 22 RV1 (Fig. 3B, D).

In summary, SLC7A11 mediates enzalutamide-induced ferroptosis suppression in CRPC cells by preserving mitochondrial membrane potential and redox homeostasis.

### Erastin combined with enzalutamide inhibits proliferation in enzalutamide-resistant cell lines

**Fig. 4:**
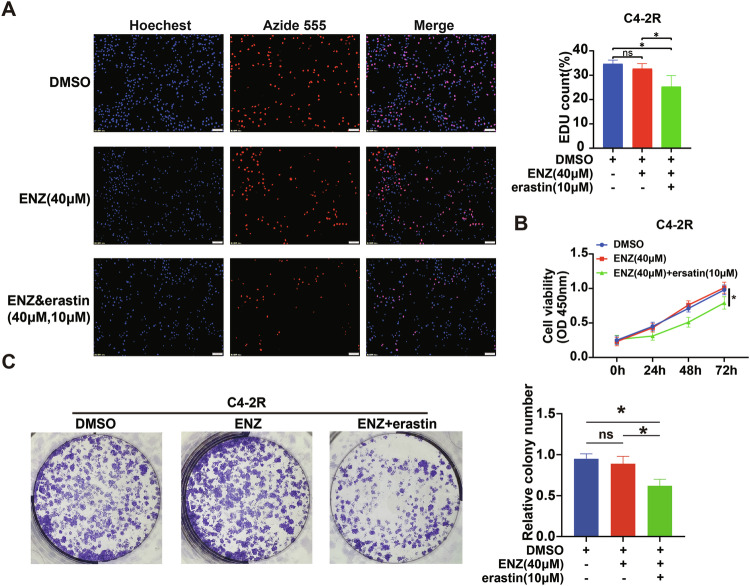
Erastin combined with enzalutamide inhibits proliferation in enzalutamide-resistant cell lines. **A** The EDU assay examined cell proliferation under drug treatment; Red fluorescence indicates actively proliferating cells. **B** Cell proliferation activity, assessed using the CCK8 method, indicated that proliferation was inhibited under these cell culture conditions. **C** Results from cell colony formation experiments showed that more colonies indicated more active proliferation. *p < 0.05, ns not significant.

Erastin is a highly effective cell-permeable inducer of ferroptosis [18]. It inhibits the activity of cystine/glutamate reverse transporter XC-system, with SLC7A11 as a crucial functional component of this system. To investigate the feasibility of targeting SLC7A11 to reverse enzalutamide resistance, we added erastin to the cell culture supernatant. First, the enzalutamide-resistant cell line C4-2R was constructed, and cell proliferation was detected in the DMSO group (carrier control), enzalutamide group (40 μM), erastin group (10 μM), and erastin combined with enzalutamide group (40 μM). In the EDU assay, we found that enzalutamide alone could not inhibit the proliferation of resistant cells, whereas erastin combined with enzalutamide significantly inhibited the proliferation of resistant cell lines (Fig. 4A–C). Similar conclusions were confirmed in cell colony formation experiments, where the number of cell colonies significantly decreased with the combination of the two drugs (Fig. 4A, C). Using the same groups, we measured cell viability via CCK8. The cell proliferation curve shifted significantly downward when enzalutamide was combined with erastin, indicating that cell proliferation was inhibited (Fig. 4B).

These results indicate that targeting SLC7A11 can inhibit the proliferation of enzalutamide-resistant cell lines.

### SLC7A11 may be post-transcriptionally regulated by AR

**Fig. 5:**
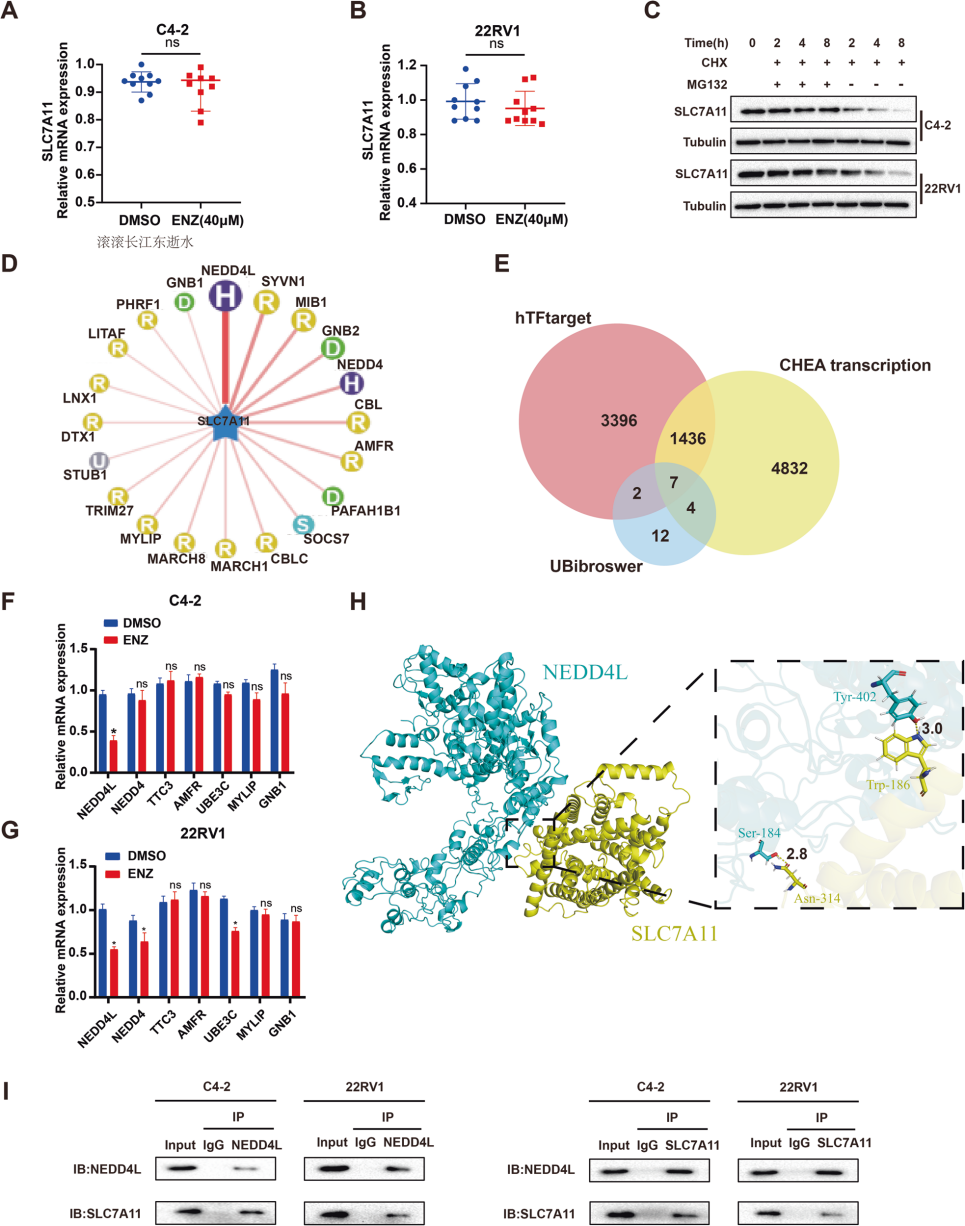
SLC7A11 may be post-transcriptionally regulated by AR. **A–C** SLC7A11 regulation occurs through proteasome degradation. **A** Comparison of SLC7A11 mRNA levels in C4-2 cells before and after treatment with enzalutamide (40 μM) (*n* = 10). **B** Analysis of SLC7A11 mRNA levels in 22RV1 cells before and after enzalutamide treatment (40 μM) (*n* = 10). **C** Investigation of SLC7A11 protein levels over time with the addition of CHX and MG132, or CHX only. **D**, **E** Database predictions identified seven AR-regulated E3 ubiquitin ligases targeting SLC7A11. **D** The predicted target of E3 ubiquitin ligase is SLC7A11. **E** Screening of E3 ubiquitin ligases that can be regulated by AR, in conjunction with transcription factor databases. **F**, **G** The expression of E3 ubiquitin ligase NEDD4L was downregulated by enzalutamide. **H** 3D interaction model of NEDD4L and SLC7A11. **I** The co-immunoprecipitation (Co-IP) results in C4-2 and 22RV1 cells demonstrated that when lysates were immunoprecipitated with anti-NEDD4L monoclonal antibody or control IgG, followed by Western blotting with anti-SLC7A11 antibody, SLC7A11 was specifically detected in NEDD4L immunoprecipitates but not in IgG controls, confirming their direct interaction. Similarly, reciprocal Co-IP using an anti-SLC7A11 antibody also detected a band corresponding to NEDD4L. *p < 0.05, ns not significant.

In this study, we detected the mRNA expression levels of SLC7A11 after enzalutamide treatment (40 μM). The results showed no significant change in SLC7A11 mRNA levels before and after treatment (Fig. 5A, B). We then added cycloheximide (CHX) and MG132 to the cell culture. Cycloheximide, an inhibitor of protein synthesis in eukaryotic cells, effectively inhibits new protein synthesis. MG132 is a proteasome inhibitor. Under CHX conditions, there was no significant change in protein levels after adding MG132 at 2, 4, and 8 h (Fig. 5C). When MG132 was not added, the protein degraded over time. This suggests that SLC7A11 is regulated by proteasome degradation.

We then explored the post-transcriptional regulation of SLC7A11 and sought to identify E3 ubiquitin ligases that specifically bind to SLC7A11. Using the UBibrowser 1.0, we predicted 25 E3 ubiquitin ligases capable of binding to SLC7A11, showing the top 20 (Fig. 5D). Using transcription factor databases hTFtarget and CHEA, we predicted 1436 AR-regulated target genes. Among these, seven E3 ubiquitin ligases were predicted to regulate SLC7A11 (Fig. 5E).

After this, we examined mRNA changes before and after treatment with enzalutamide (40 μM), and further screened for E3 ubiquitin ligase. The results showed significant changes in NEDD4L in both the C4-2 and 22RV1 cell lines (Fig. 5F, G). The affinity between NEDD4L and SLC7A11 was then tested using molecular docking techniques. The side chain hydroxyl O-H of the Ser184 residue of the NEDD4L protein formed a hydrogen bond, with a bond length of 2.8 Å, with the side chain amide O of the Asn314 residue of the SLC7A11 protein. Additionally, the side chain phenol hydroxyl O-H of the Tyr402 residue of NEDD4L formed a 3.0 Å hydrogen bond with the side chain indole N of the Trp186 residue of SLC7A11. The binding free energy between the NEDD4L and SLC7A11 protein was calculated as −25.589 kcal/mol using Rosetta’s InterfaceAnalyzer. Through Interface_energy module analysis, the pairing energy between the Ser184 residue of NEDD4L and Asn314 residue of SLC7A11 was −1.403 kcal/mol. The pairing energy between the Ser184 and Asn314 residues was −0.324 kcal/mol, and the overall interface energy was −1.727 kcal/mol. The 3D configuration of the molecular docking was also generated (Fig. 5H). These results indicate a high affinity between NEDD4L and SLC7A11.

These findings suggest that SLC7A11 may be regulated by ubiquitination through AR, with NEDD4L potentially acting as an E3 ubiquitin ligase in this regulatory process. To further clarify this mechanism, we confirmed the interaction between NEDD4L and SLC7A11 using co-immunoprecipitation (co-IP) assays (Fig. 5I), thereby further corroborating our conclusion.

### In vivo experiments demonstrated that targeting SLC7A11 can increase sensitivity to enzalutamide

**Fig. 6:**
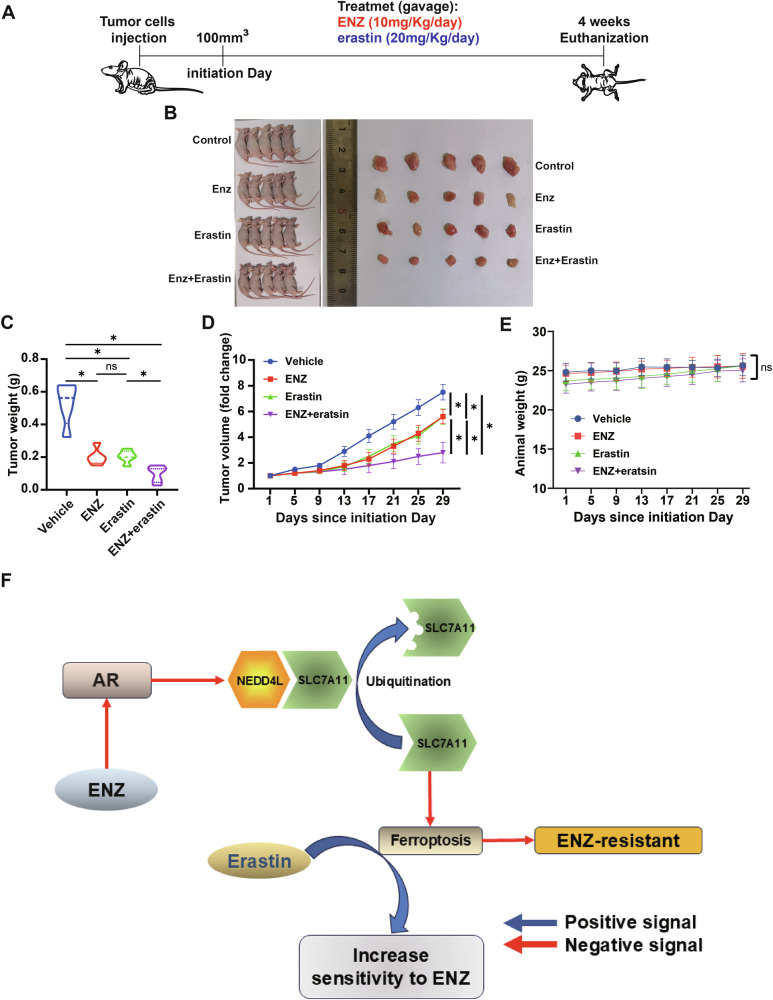
In vivo experiments demonstrated that targeting SLC7A11 can increase sensitivity to enzalutamide. **A** Experimental plan for subcutaneous tumor formation in nude mice. **B** Visualization of tumor model and tumor body. **C** Measurement of tumor weight. **D** Tumor volume growth curve. **E** Weight change curve of nude mice over time. **F** The mechanistic diagram of this study. *p < 0.05, ns not significant.

We constructed a prostate cancer animal model using nude mice, which were randomly assigned to four groups. Enzalutamide (10 mg/kg/day) or erastin (20 mg/kg/day) was administered by intragastric administration after the subcutaneous tumor reached 100 mm^3^ (Fig. 6A, B). After 4 weeks, tumor volume and weight were measured. The results showed that tumor weight was significantly lower in the groups receiving enzalutamide or erastin compared to the control group. The tumor volume in the combination group (enzalutamide and erastin) was lower than that in either the enzalutamide or erastin groups alone (Fig. 6C). This pattern was also confirmed in volume measurement (Fig. 6D). There was no significant difference in the body weight of the mice (Fig. 6E).

Together, these results suggest that targeting SLC7A11 with erastin can effectively enhance the sensitivity to enzalutamide (Fig. 6F).

## Discussion

In this study, we found that there was a concentration-dependent decrease in ferroptosis in CRPC cell lines when different concentrations of enzalutamide were added. This process relies on the post-translational regulation of SLC7A11 by enzalutamide. It is likely that enzalutamide regulates the ubiquitization level of SLC7A11 through AR. It is predicted that NEDD4L may be an important E3 ubiquitin ligase, as suggested by database predictions, and it may serve as a bridge between AR and SLC7A11. Moreover, we verified the interaction between NEDD4L and SLC7A11 through co–IP experiments, which further strengthened our conclusion.

The main mechanism of action of enzalutamide is its ability to directly bind to AR, inhibit androgen-AR binding, androgen receptor nuclear translocation, and androgen receptor-mediated DNA binding. Current research on enzalutamide resistance mechanisms includes the following aspects: 1. Androgen receptor splicing variants. Androgen receptor splicing variant V7 (AR-V7) is the main variant, leading to the expression of androgen receptors in regions lacking ligand-binding proteins [8]. This region is critical for the effectiveness of enzalutamide, and patients who are AR-V7-positive have significantly lower overall survival rates compared to AR-V7-negative patients. 2. Androgen receptor reactivation. This occurs through mediated activation of AR levels by genes or proteins such as cAMP response element binding protein 5 (CREB5) [19], precursor factor FOXA1 [20], proto-oncogene cell myelomatosis oncogene (C-MYC) [21], and eukaryotic translation initiation factor 4F (eIF4F) [22]. 3. Androgen receptor mutation. Most mutations occur in the carboxyl terminal ligand binding domain, and AR F876L mutations can convert the androgen receptor antagonist enzalutamide into an androgen receptor agonist [23]. Additionally, enzalutamide resistance may involve overexpression of glucocorticoid receptor [24], activation of Wnt signal [25], glycolysis [26], and other factors.

Numerous studies have shown that ferroptosis is an adaptive process critical for the elimination of carcinogenic cells [27]. Several studies have demonstrated the potential of ferroptosis inducers in cancer treatment. For instance, erastin can inhibit the progression of triple-negative breast cancer [28] and reverse ABCB1-mediated docetaxel resistance in ovarian cancer [29]. Drugs that have been in clinical use for many years have also been linked to ferroptosis. For example, salazosulfodiazine effectively inhibits the Xc-system [30], and lanperidone alters the gating of VDAC (Mitochondrial voltage-dependent anion channels), leading to mitochondrial dysfunction, ROS production, and ultimately ferroptosis [31]. Cisplatin inhibits peroxidase activity, and studies show that combining cisplatin with a ferroptosis inducer is more effective than cisplatin alone [32]. This shows that ferroptosis has the potential to impact various human diseases. Developing new drugs or therapies based on ferroptosis has promising prospects.

Sensitivity to ferroptosis is closely linked to various biological processes, including those involving amino acids, iron, polyunsaturated fatty acids, and glutathione (GSH) metabolism. Targeting these pathways may modulate the sensitivity of tumor cells to ferroptosis [33]. Tumor cells often require large amounts of GSH to neutralize intracellular ROS. Cysteine, the rate-limiting precursor of GSH synthesis, cannot be synthesized by tumor cells and must be imported from the extracellular environment. This process relies on the cystine/glutamate antiporter SLC7A11 (solute carrier family 7 member 11), part of the system Xc- [34]. SLC7A11 imports cystine into the cell and exports glutamate in a 1:1 ratio. Once inside the cell, cystine is reduced to cysteine via a reaction involving NADPH. Cysteine is then used to synthesize GSH through a two-step process: first, γ-glutamylcysteine is formed by combining cysteine with glutamate, catalyzed by γ-glutamylcysteine synthetase (γ-GCS). Next, γ-glutamylcysteine binds with glycine, catalyzed by glutathione synthetase (GS), to form GSH. When SLC7A11 function is inhibited, lipid peroxides and ROS accumulate, leading to ferroptosis. High SLC7A11 expression is associated with poor prognosis in several cancers, including acute myeloid leukemia, breast cancer, ovarian cancer, and colon cancer. A study by Weixiong Zhong et al. showed that immunohistochemistry of tumor microarrays from prostate cancer tissues (*N* = 165) with high Gleason scores revealed significant increases in xCT protein expression. Ghoochani et al. also demonstrated that SLC7A11 inhibitors significantly inhibit the growth of prostate cancer cells [35].

Ubiquitination refers to the process by which ubiquitin molecules bind to target proteins for specific modification or degradation via the proteasome pathway, catalyzed by various enzymes, including ubiquitin-activating enzymes, ubiquitin-conjugating enzymes, ubiquitin ligases, and deubiquitinating enzymes. Ubiquitination plays a vital role in processes such as the cell cycle, apoptosis, differentiation, metastasis, gene expression, and transcription regulation. It is also crucial in inflammation, tissue repair, and tumorigenesis, including prostate cancer [36]. Studies have shown that ERK1/2 inhibits the Cullin 3/SPOP-mediated ubiquitination and degradation of PrLZ, regulating prostate cancer progression [37]. Additionally, PELO promotes prostate cancer progression by enhancing PLK1-induced ubiquitination and degradation of Smad4 [38]. This study is the first to report that AR-mediated SLC7A11 upregulation and NEDD4L ubiquitination together contribute to ferroptosis inhibition and enzalutamide resistance in CRPC.

However, there are limitations to this study. For instance, there is a lack of validation using clinical tissue samples. This is partly due to the challenges of collecting relevant samples from sporadic clinical patients with enzalutamide resistance. In future studies, it will be necessary to continuously accumulate samples and verify these findings. Additionally, more research is needed to further elucidate the regulation of SLC7A11 ubiquitination.

In this study, we found that Enz stabilizes SLC7A11 by inhibiting NEDD4L-mediated ubiquitination, thereby enhancing cystine uptake and GSH synthesis. GSH supports GPX4 activity to neutralize lipid peroxides, preserving mitochondrial integrity and suppressing ROS. This AR/SLC7A11/ferroptosis axis provides a novel mechanistic basis for overcoming enzalutamide resistance in CRPC.

## Supplementary information

Full and uncropped western blots

## Data Availability

Data were generated by the authors and deposited in a repository.
