# Correction: SLC7A11 upregulation via AR and NEDD4L ubiquitination contributes to ferroptosis inhibition and enzalutamide resistance in castration-resistant prostate cancer

**DOI:** 10.1038/s41419-025-08123-9

**Published:** 2025-11-18

**Authors:** Min Tang, Luotong Xue, Bao Wang, Zhixin Jiang, Pu Li, Hengtao Bu, Chesong Zhao, Yurong Zhang, Shuaimei Liu, Xiyuan Chen, Bianjiang Liu, Xiaoxin Meng, Jie Li

**Affiliations:** 1 Department of Urology, The First Affiliated Hospital with Nanjing Medical University, Nanjing, Jiangsu China; 2 NHC Key Laboratory of Contraceptives Vigilance and Fertility Surveillance, Nanjing, Jiangsu China; 3 Jiangsu Provincial Medical Key Laboratory of Fertility Protection and Health Technology Assessment, Nanjing, Jiangsu China; 4 Jiangsu Health Development Research Center, Nanjing, Jiangsu China; 5 Department of Urology, Shuyang Hospital, Suqian, Jiangsu China

**Keywords:** Drug development, Cancer models

Correction to: *Cell Death and Disease* 10.1038/s41419-025-07809-4, published online 05 August 2025

After publication, we noticed several inadvertent errors in the figure and figure legends, which might affect the fluency of reading. These are typographical mistakes and do not affect the scientific conclusions of the study.

1. Figure 5I (There was a typographical error in the cell line name)
  Incorrect: “C2-4”
  Correct: “C4-2”
2. Fig. 1 legend, Line 2
  Incorrect: “GSH levels in 22RV1 (A) and C4-2 (B) cell lines...”
  Correct: “GSH levels in C4-2 (A) and 22RV1 (B) cell lines...”
3. Fig. 5B legend
  Incorrect: “Analysis of mRNA levels in C4-2 cells before and after...”
  Correct: “Analysis of SLC7A11 mRNA levels in 22RV1 cells before and after...”

**Original Figure 5** 
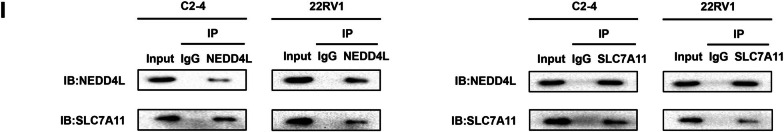


**Amended Figure 5** 
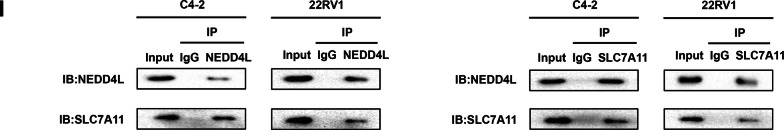


The original article has been corrected.

