# Correction: SLC7A11 upregulation via AR and NEDD4L ubiquitination contributes to ferroptosis inhibition and enzalutamide resistance in castration-resistant prostate cancer

**DOI:** 10.1038/s41419-026-08741-x

**Published:** 2026-05-05

**Authors:** Min Tang, Luotong Xue, Bao Wang, Zhixin Jiang, Pu Li, Hengtao Bu, Chesong Zhao, Yurong Zhang, Shuaimei Liu, Xiyuan Chen, Bianjiang Liu, Xiaoxin Meng, Jie Li

**Affiliations:** 1 Department of Urology, The First Affiliated Hospital with Nanjing Medical University, Nanjing, Jiangsu China; 2 NHC Key Laboratory of Contraceptives Vigilance and Fertility Surveillance, Nanjing, Jiangsu China; 3 Jiangsu Provincial Medical Key Laboratory of Fertility Protection and Health Technology Assessment, Nanjing, Jiangsu China; 4 Jiangsu Health Development Research Center, Nanjing, Jiangsu China; 5 Department of Urology, Shuyang Hospital, Suqian, Jiangsu China

**Keywords:** Drug development, Cancer models

Correction to: *Cell Death & Disease* 10.1038/s41419-025-08123-9, published online 18 November 2025

During our recent internal self-inspection, we identified that two IP bands in Figure 5I appear highly similar. After careful reflection and thorough analysis by the research team, we determined that this band duplication was caused by an inadvertent error during figure preparation. The original data for the manuscript have been fully preserved, are authentic and reliable, and were submitted to the journal at the time of initial submission. This image misuse does not affect the scientific integrity of the study or the validity of its conclusions.

**Original Figure 5** 
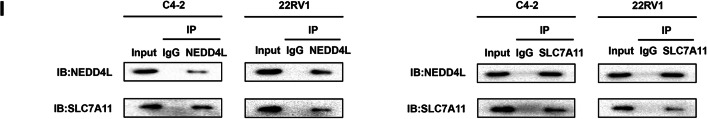


**Amended Figure 5** 
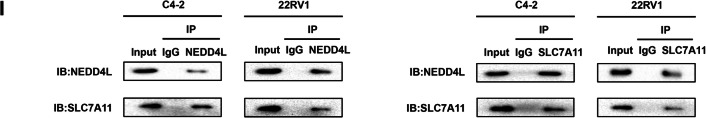


The original article has been corrected.

