# Supplementary figures and images for: SLC7A11 upregulation via AR and NEDD4L ubiquitination contributes to ferroptosis inhibition and enzalutamide resistance in castration-resistant prostate cancer (part 2 of 2)
Source: Cell Death Dis. 2025 Aug 5;16(1):591. doi: 10.1038/s41419-025-07809-4 (PMC12325610; doi:10.1038/s41419-025-07809-4)

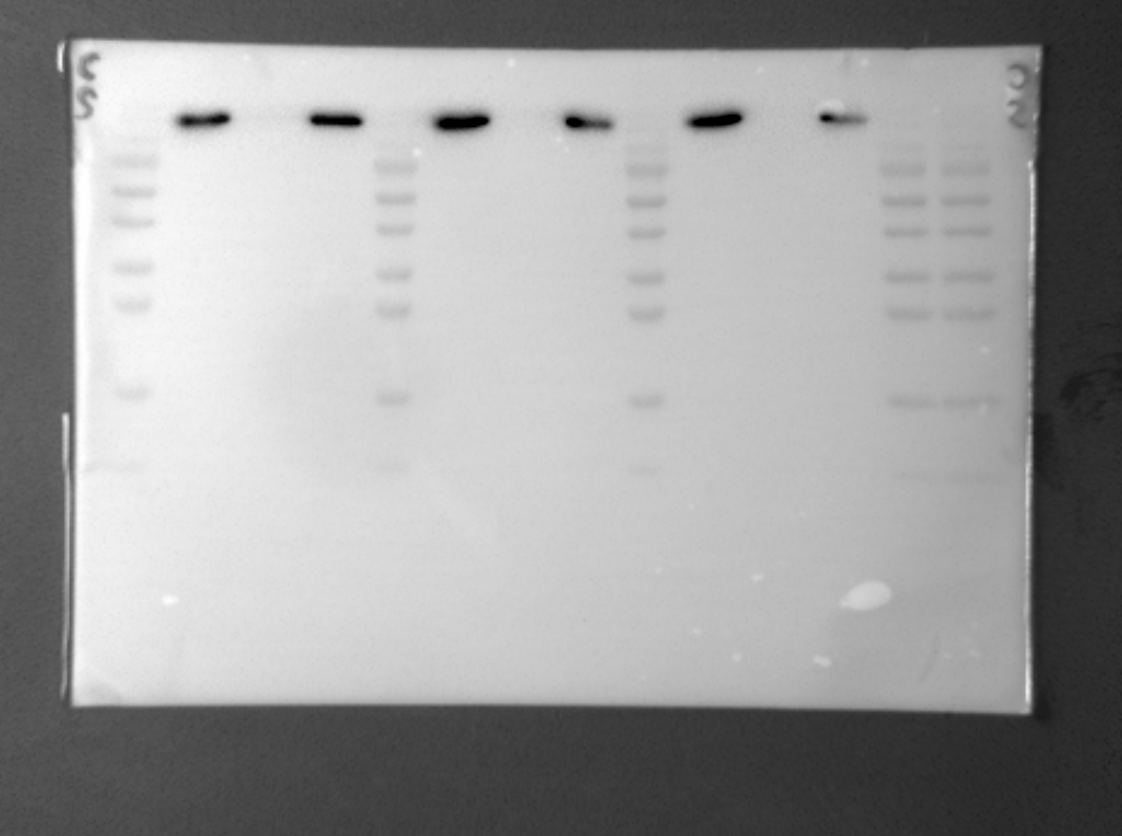

Supplement: Supplementary file 1 — Full and uncropped western blots [file 41419_2025_7809_MOESM1_ESM.zip › Full and uncropped western blots/Fig5I-co-ip/IP-SLC7A11/IB-NEDD4L/Merge C42.tif]

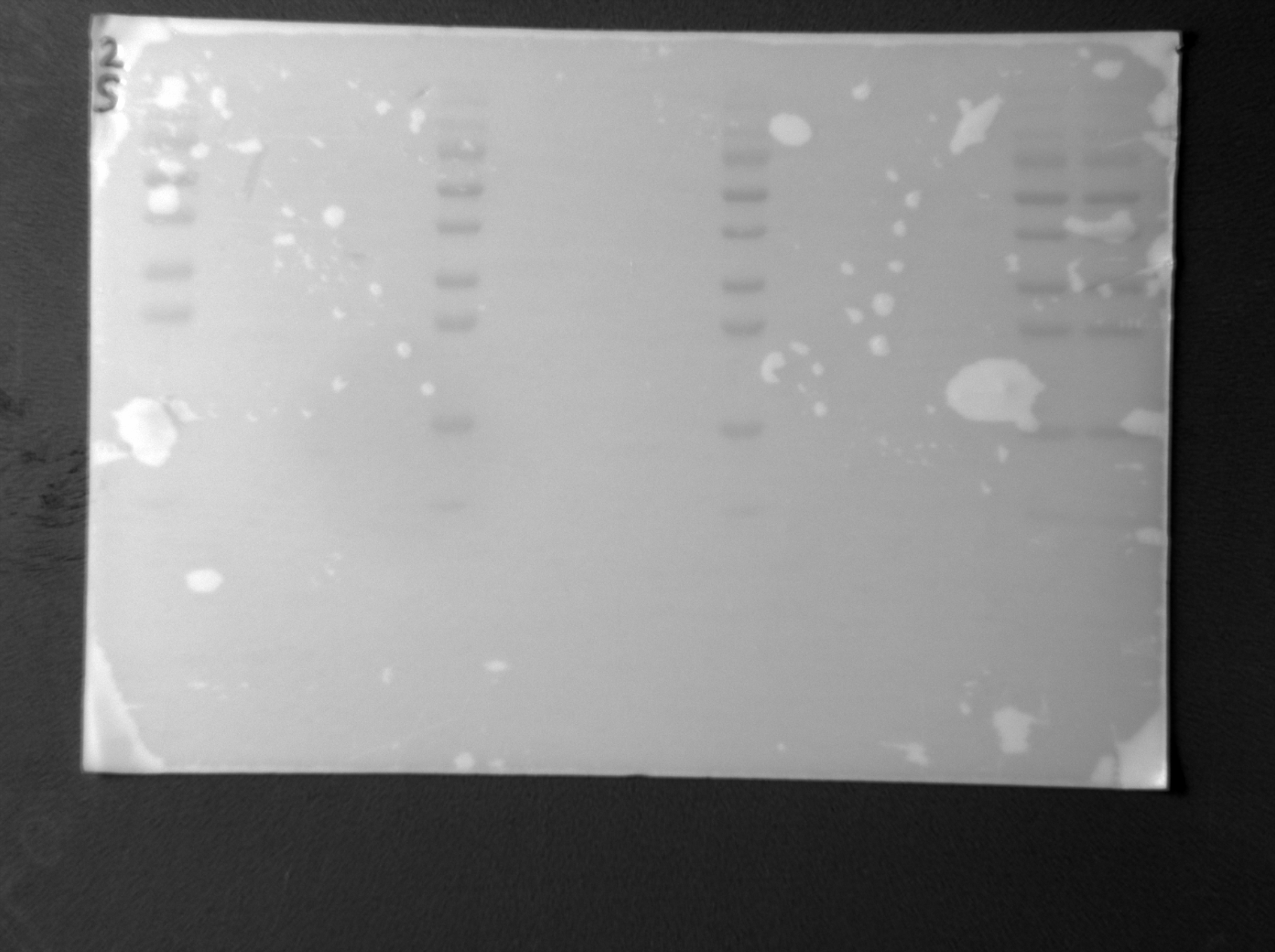

Supplement: Supplementary file 1 — Full and uncropped western blots [file 41419_2025_7809_MOESM1_ESM.zip › Full and uncropped western blots/Fig5I-co-ip/IP-SLC7A11/IB-NEDD4L/picture of film 22RV1.tif]

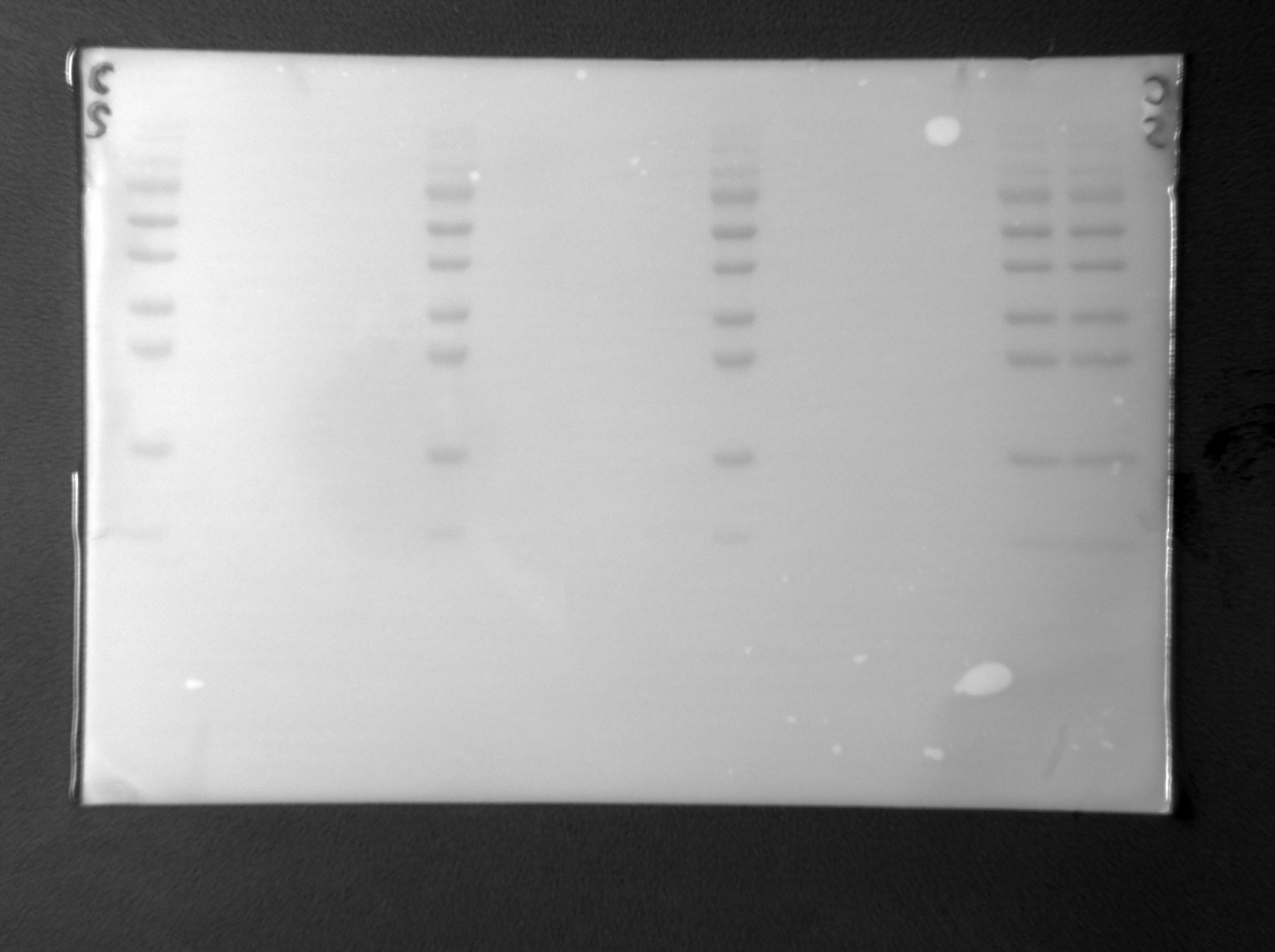

Supplement: Supplementary file 1 — Full and uncropped western blots [file 41419_2025_7809_MOESM1_ESM.zip › Full and uncropped western blots/Fig5I-co-ip/IP-SLC7A11/IB-NEDD4L/picture of film C42.tif]

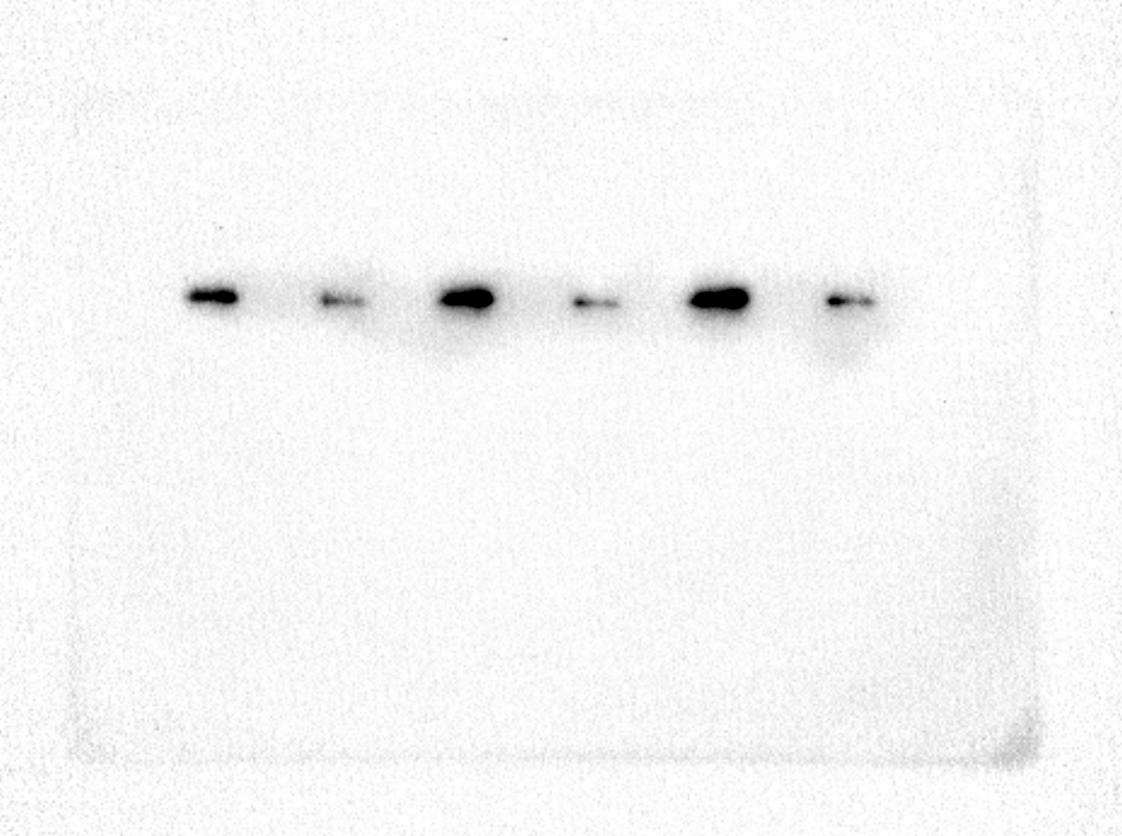

Supplement: Supplementary file 1 — Full and uncropped western blots [file 41419_2025_7809_MOESM1_ESM.zip › Full and uncropped western blots/Fig5I-co-ip/IP-SLC7A11/IB-SLC7A11/IP SLC7A11+IB SLC7A11 22RV1.tif]

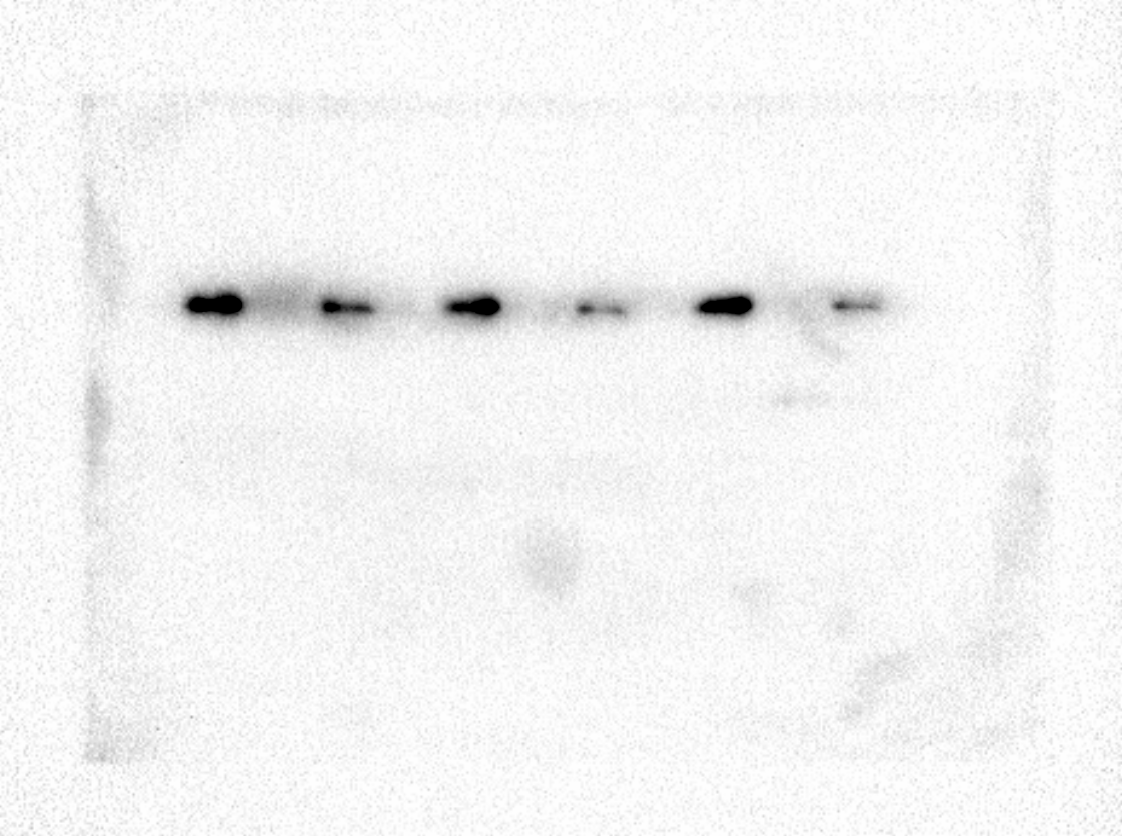

Supplement: Supplementary file 1 — Full and uncropped western blots [file 41419_2025_7809_MOESM1_ESM.zip › Full and uncropped western blots/Fig5I-co-ip/IP-SLC7A11/IB-SLC7A11/IP SLC7A11+IB SLC7A11 C42.tif]

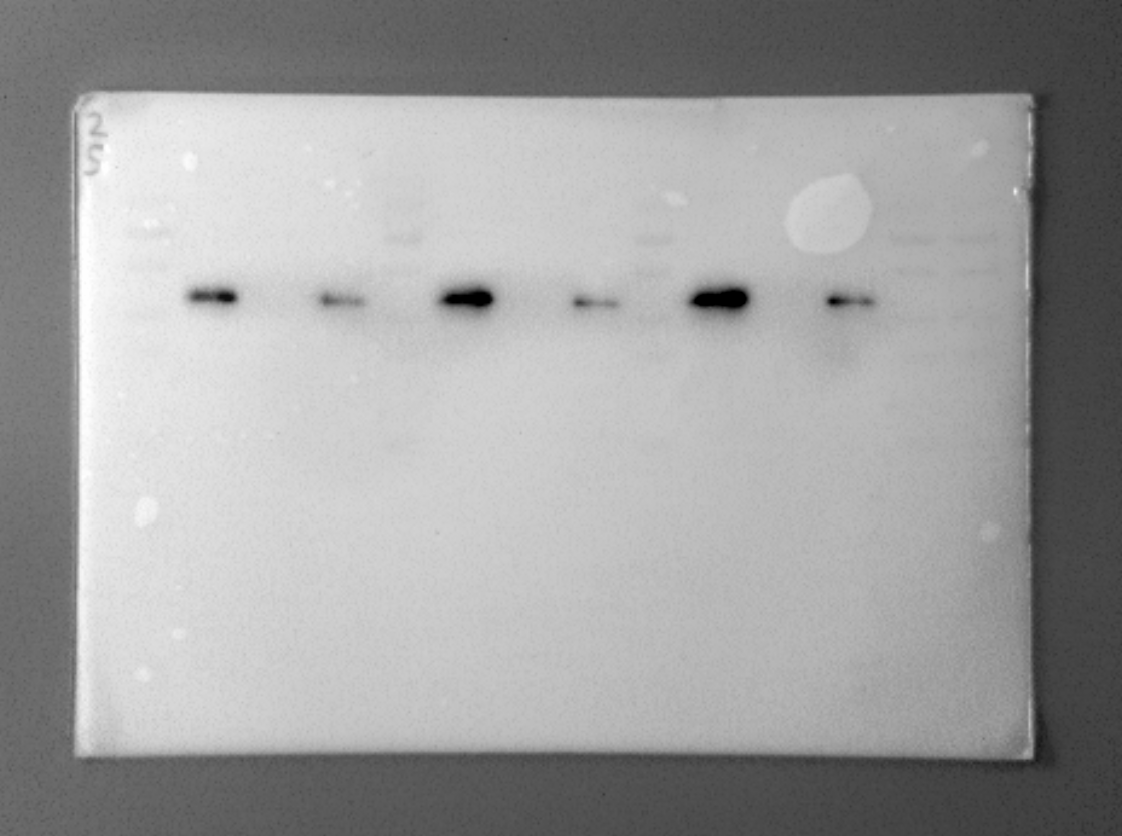

Supplement: Supplementary file 1 — Full and uncropped western blots [file 41419_2025_7809_MOESM1_ESM.zip › Full and uncropped western blots/Fig5I-co-ip/IP-SLC7A11/IB-SLC7A11/Merge 22RV1.tif]

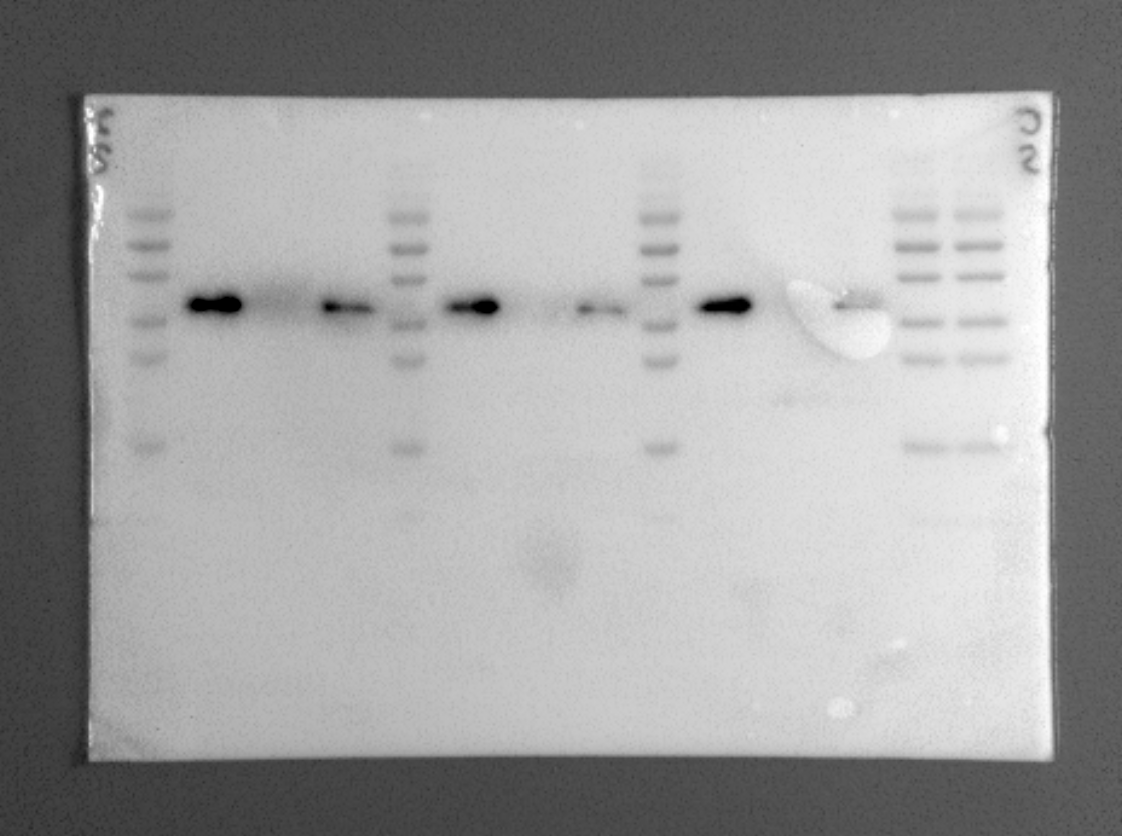

Supplement: Supplementary file 1 — Full and uncropped western blots [file 41419_2025_7809_MOESM1_ESM.zip › Full and uncropped western blots/Fig5I-co-ip/IP-SLC7A11/IB-SLC7A11/Merge C42.tif]

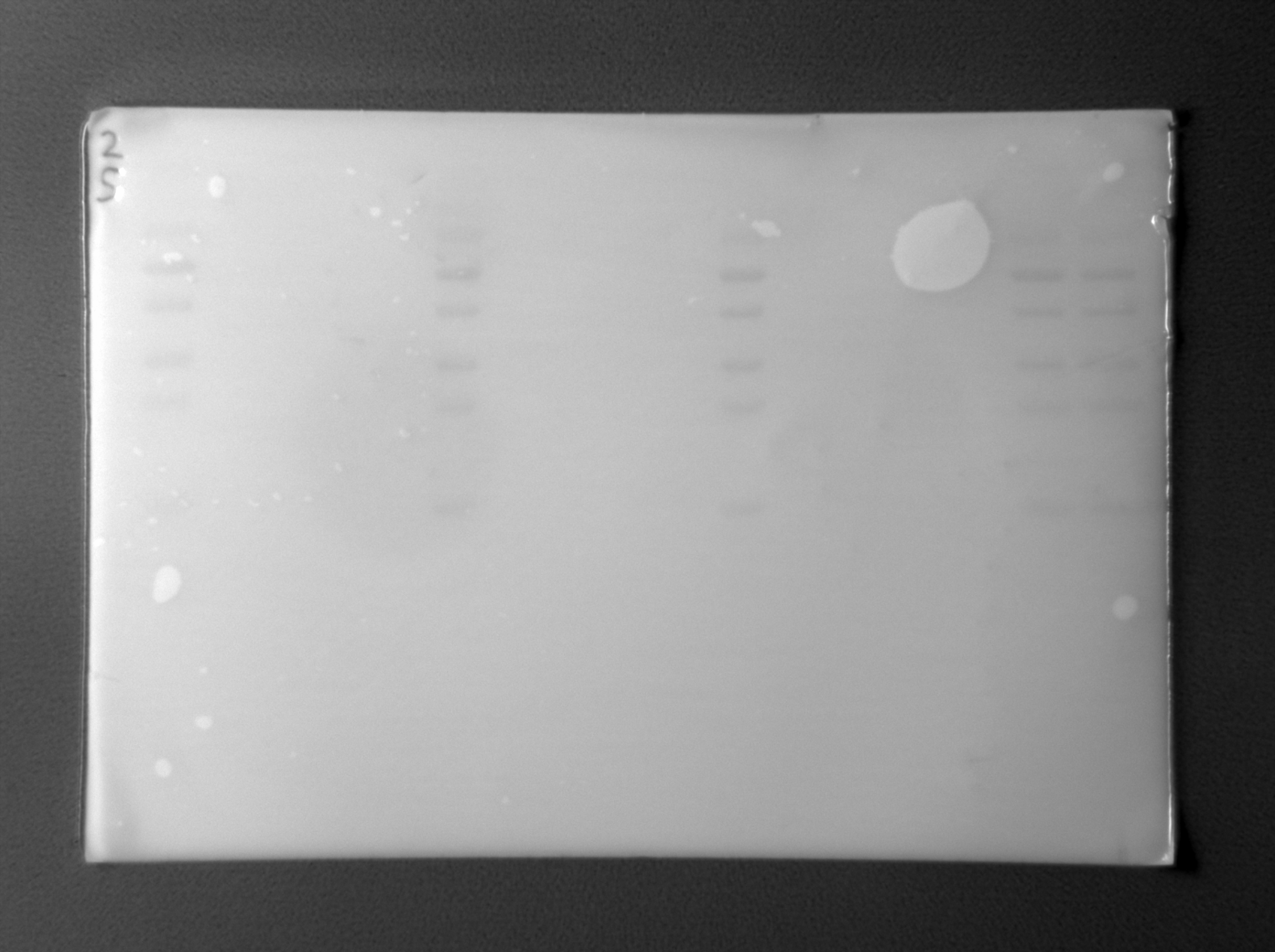

Supplement: Supplementary file 1 — Full and uncropped western blots [file 41419_2025_7809_MOESM1_ESM.zip › Full and uncropped western blots/Fig5I-co-ip/IP-SLC7A11/IB-SLC7A11/picture of film 22RV1.tif]

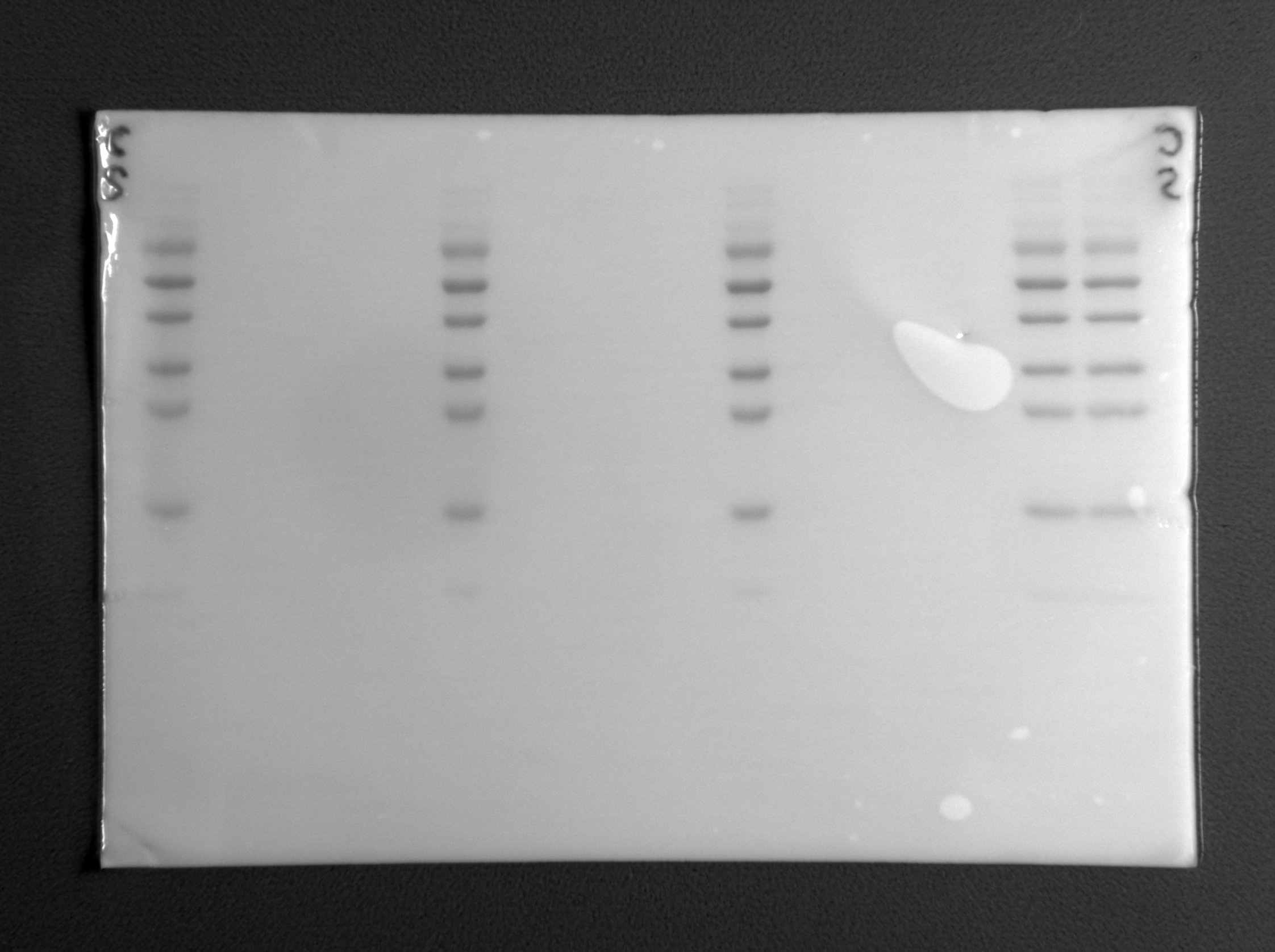

Supplement: Supplementary file 1 — Full and uncropped western blots [file 41419_2025_7809_MOESM1_ESM.zip › Full and uncropped western blots/Fig5I-co-ip/IP-SLC7A11/IB-SLC7A11/picture of film C42.tif]
