# Supplementary figures and images for: SLC7A11 upregulation via AR and NEDD4L ubiquitination contributes to ferroptosis inhibition and enzalutamide resistance in castration-resistant prostate cancer (part 1 of 2)
Source: Cell Death Dis. 2025 Aug 5;16(1):591. doi: 10.1038/s41419-025-07809-4 (PMC12325610; doi:10.1038/s41419-025-07809-4)

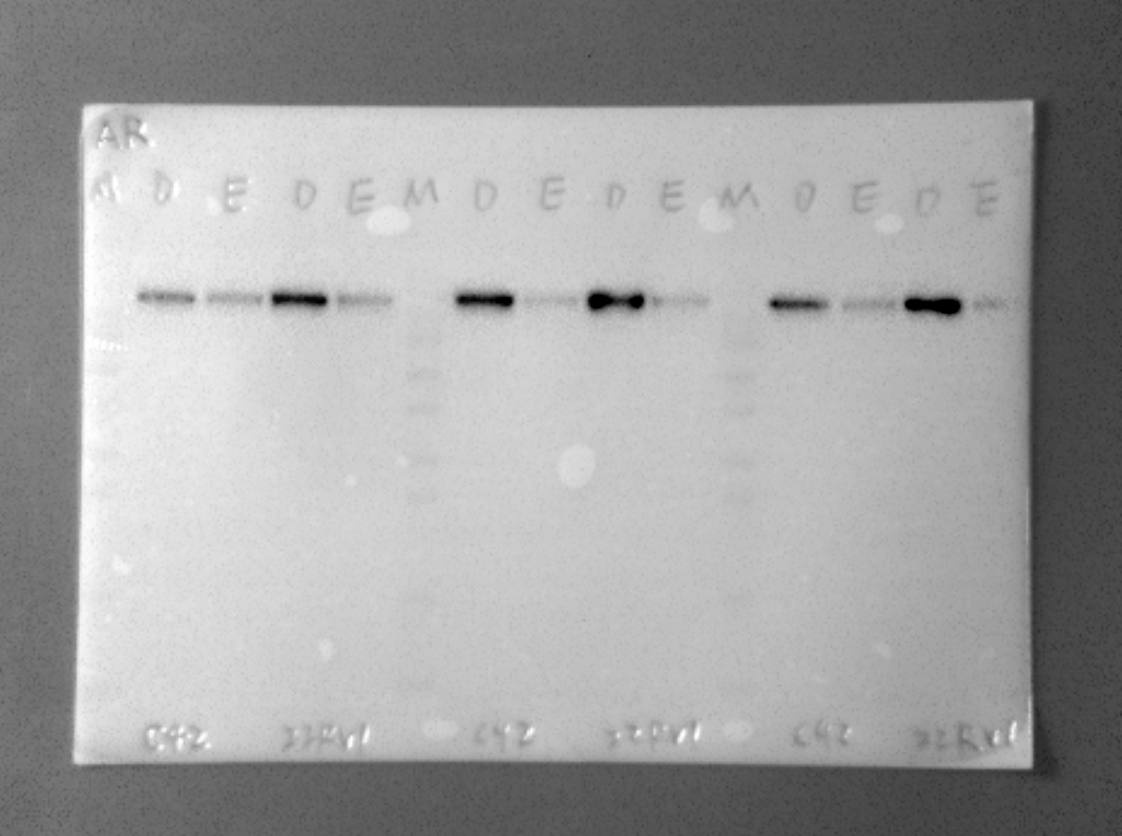

Supplement: Supplementary file 1 — Full and uncropped western blots [file 41419_2025_7809_MOESM1_ESM.zip › Full and uncropped western blots/Fig2A/AR/AR-Merge.tif]

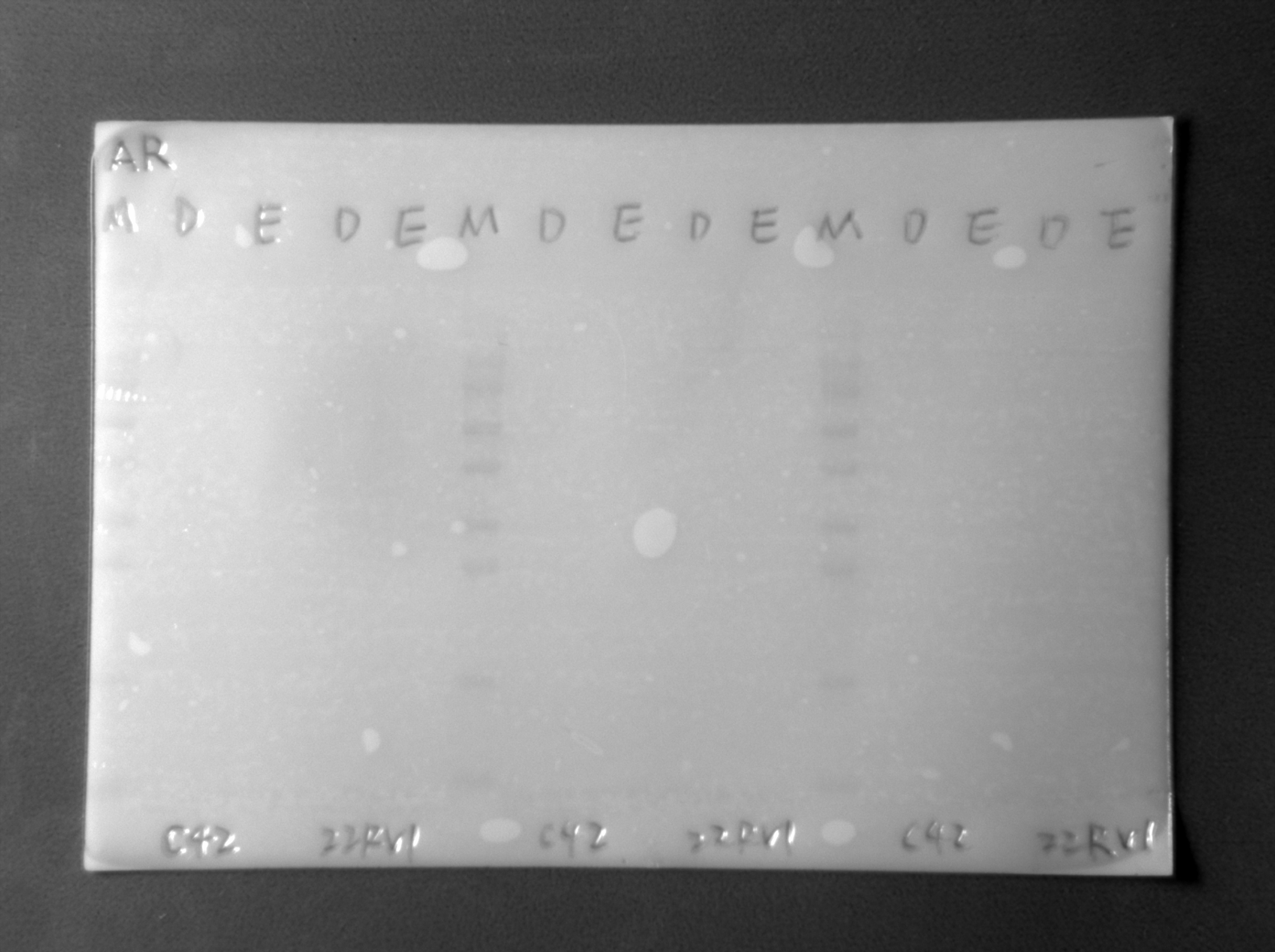

Supplement: Supplementary file 1 — Full and uncropped western blots [file 41419_2025_7809_MOESM1_ESM.zip › Full and uncropped western blots/Fig2A/AR/AR-picture of film.tif]

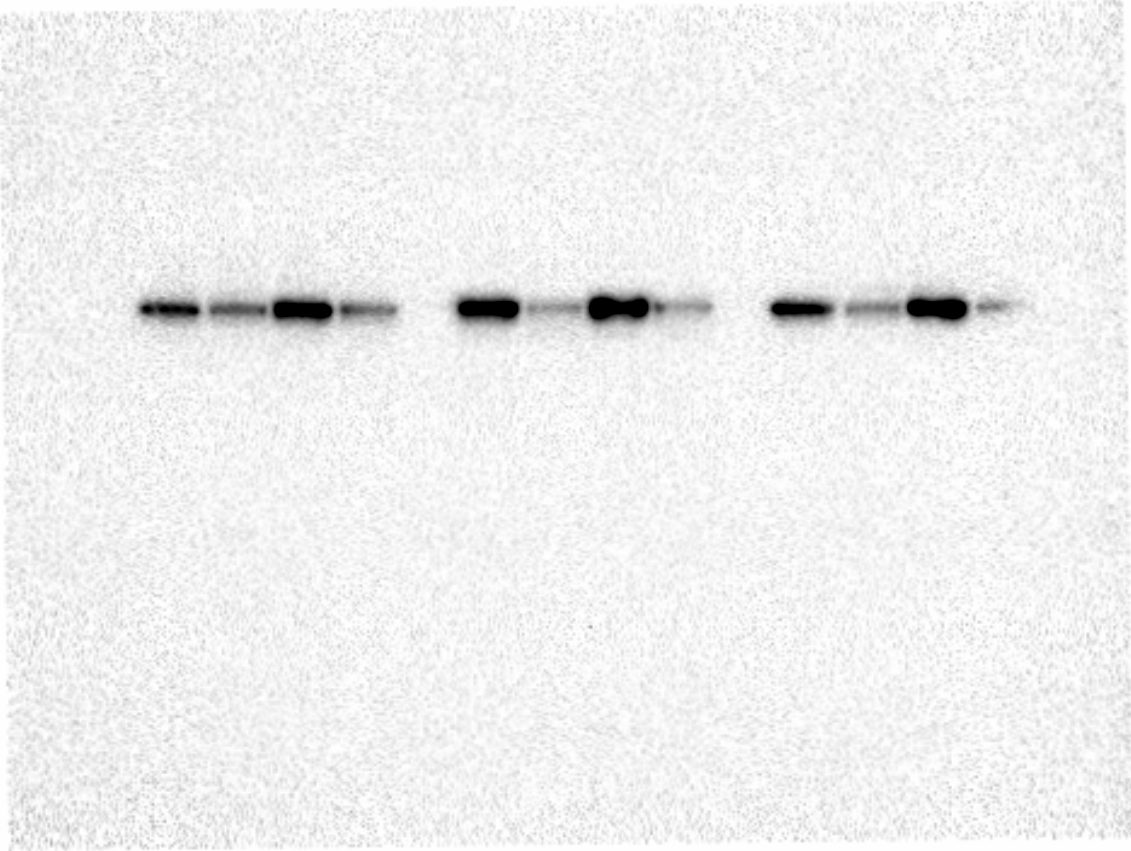

Supplement: Supplementary file 1 — Full and uncropped western blots [file 41419_2025_7809_MOESM1_ESM.zip › Full and uncropped western blots/Fig2A/AR/AR.tif]

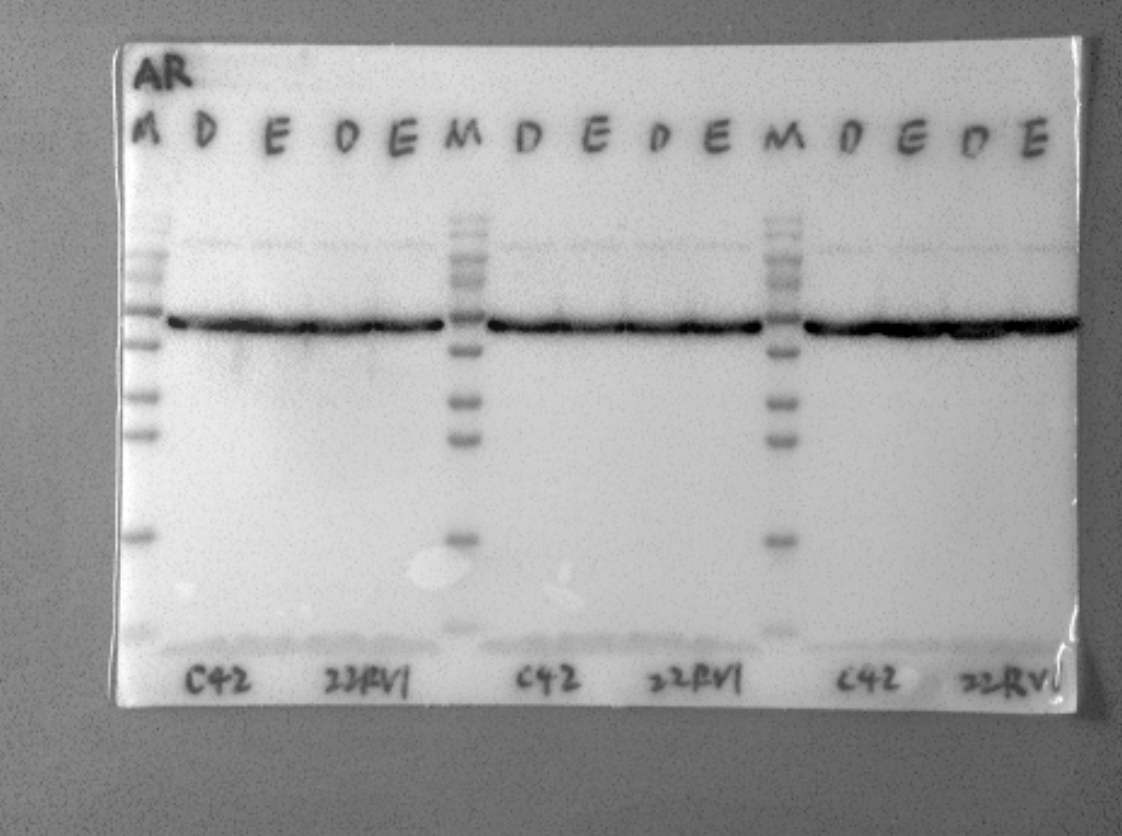

Supplement: Supplementary file 1 — Full and uncropped western blots [file 41419_2025_7809_MOESM1_ESM.zip › Full and uncropped western blots/Fig2A/AR/Tubulin-Merge.tif]

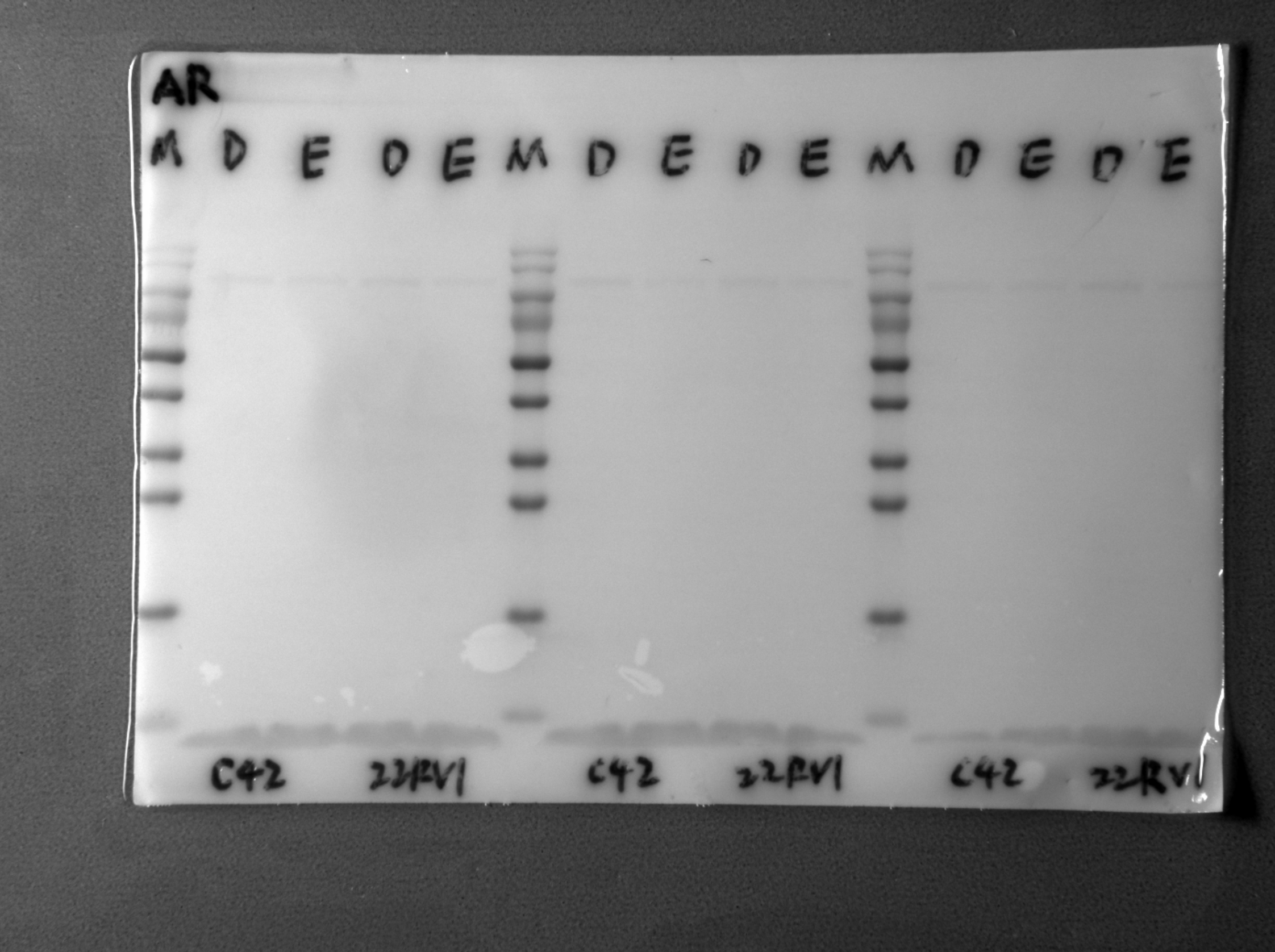

Supplement: Supplementary file 1 — Full and uncropped western blots [file 41419_2025_7809_MOESM1_ESM.zip › Full and uncropped western blots/Fig2A/AR/Tubulin-picture of film.tif]

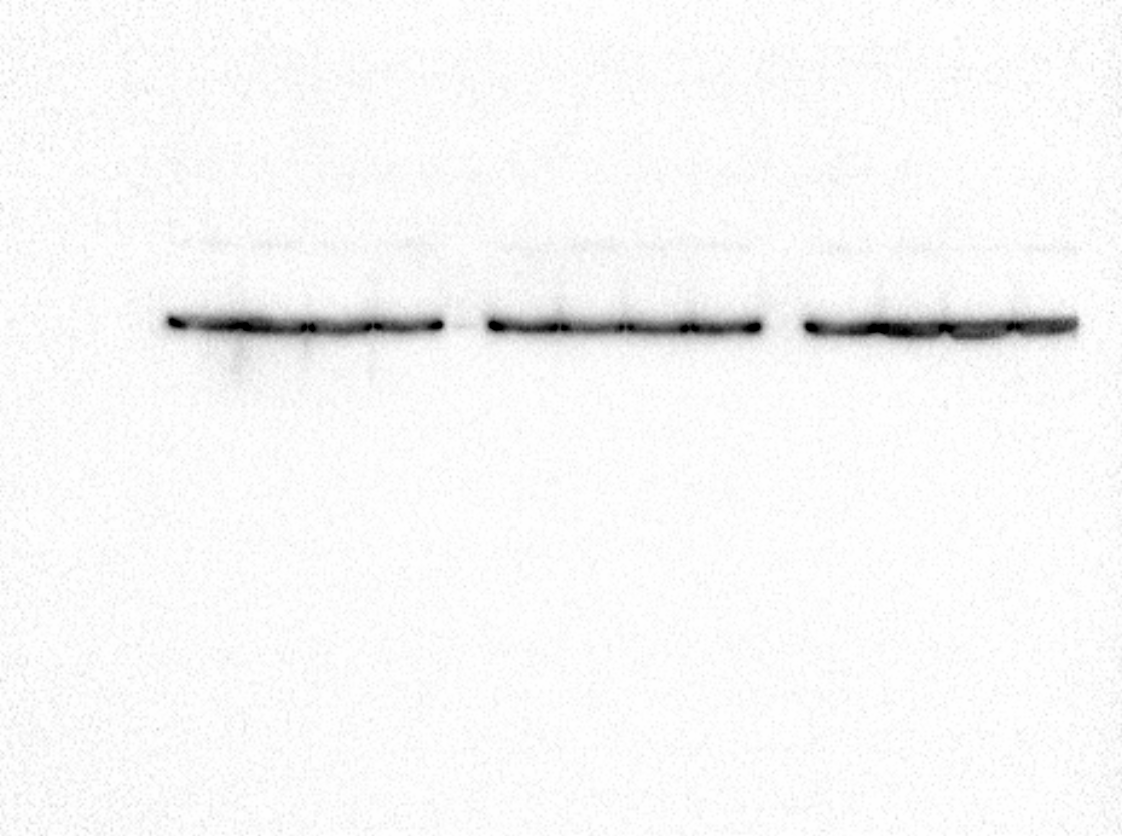

Supplement: Supplementary file 1 — Full and uncropped western blots [file 41419_2025_7809_MOESM1_ESM.zip › Full and uncropped western blots/Fig2A/AR/Tubulin.tif]

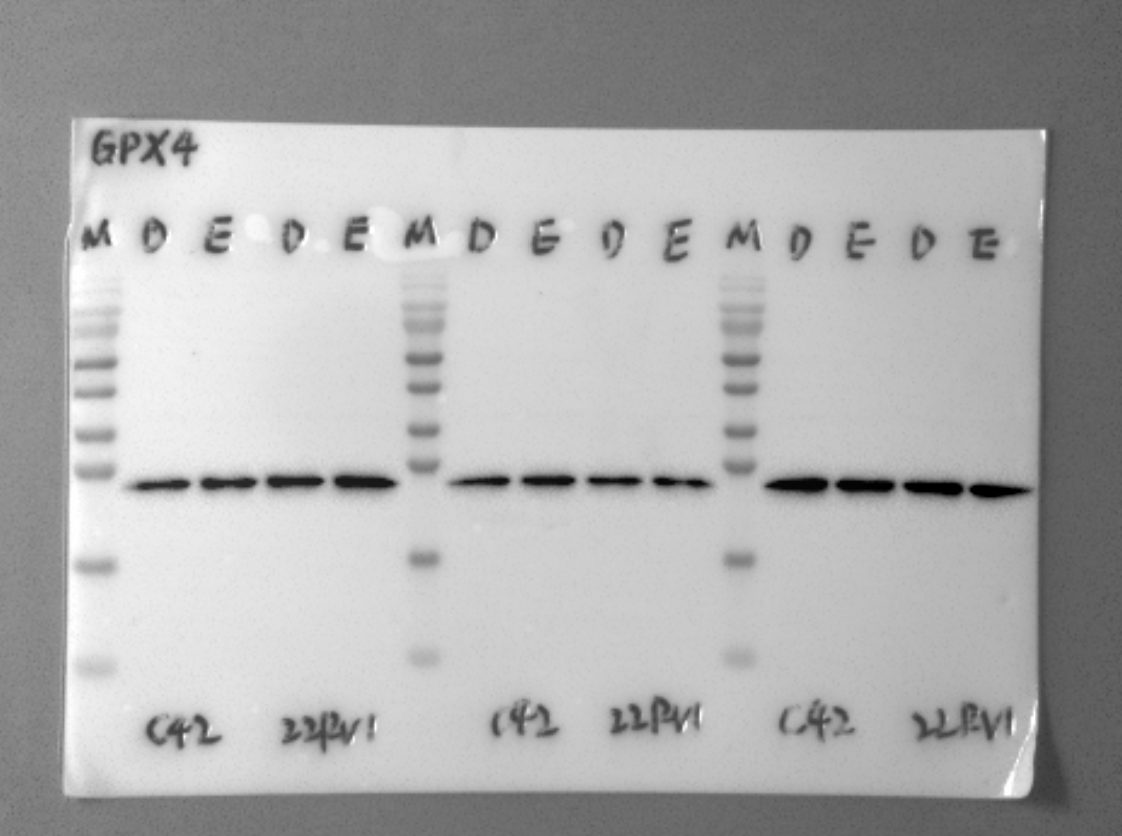

Supplement: Supplementary file 1 — Full and uncropped western blots [file 41419_2025_7809_MOESM1_ESM.zip › Full and uncropped western blots/Fig2A/GPX4/GPX4-Merge.tif]

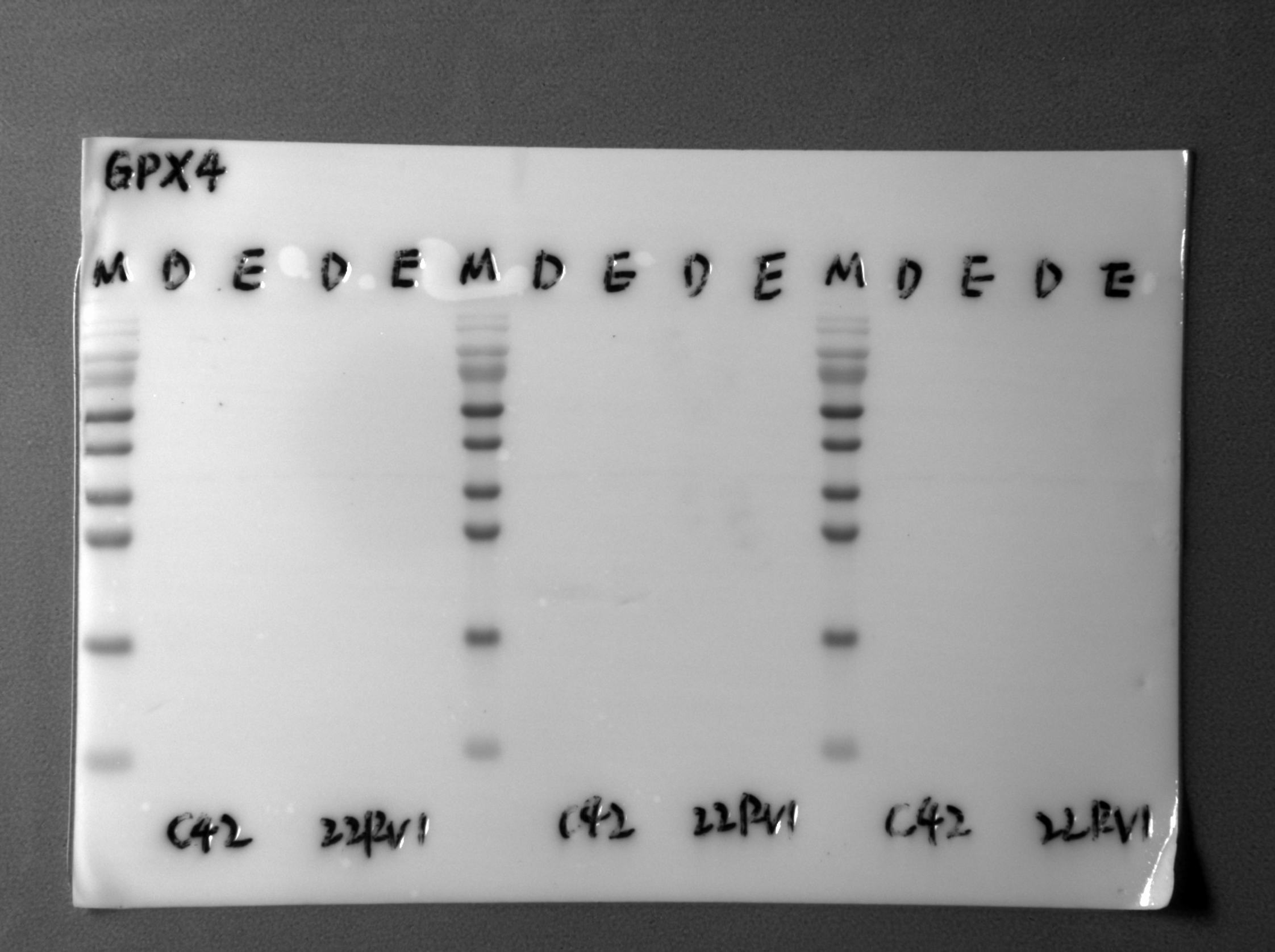

Supplement: Supplementary file 1 — Full and uncropped western blots [file 41419_2025_7809_MOESM1_ESM.zip › Full and uncropped western blots/Fig2A/GPX4/GPX4-picture of film.tif]

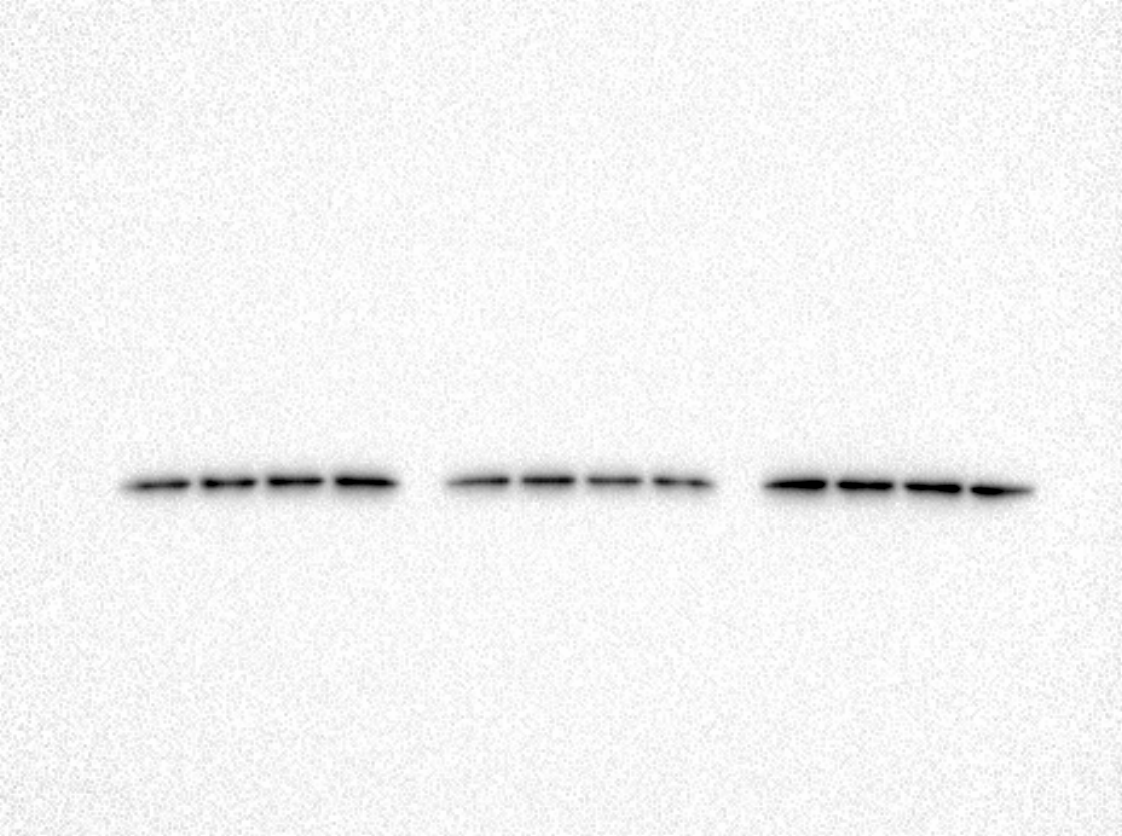

Supplement: Supplementary file 1 — Full and uncropped western blots [file 41419_2025_7809_MOESM1_ESM.zip › Full and uncropped western blots/Fig2A/GPX4/GPX4.tif]

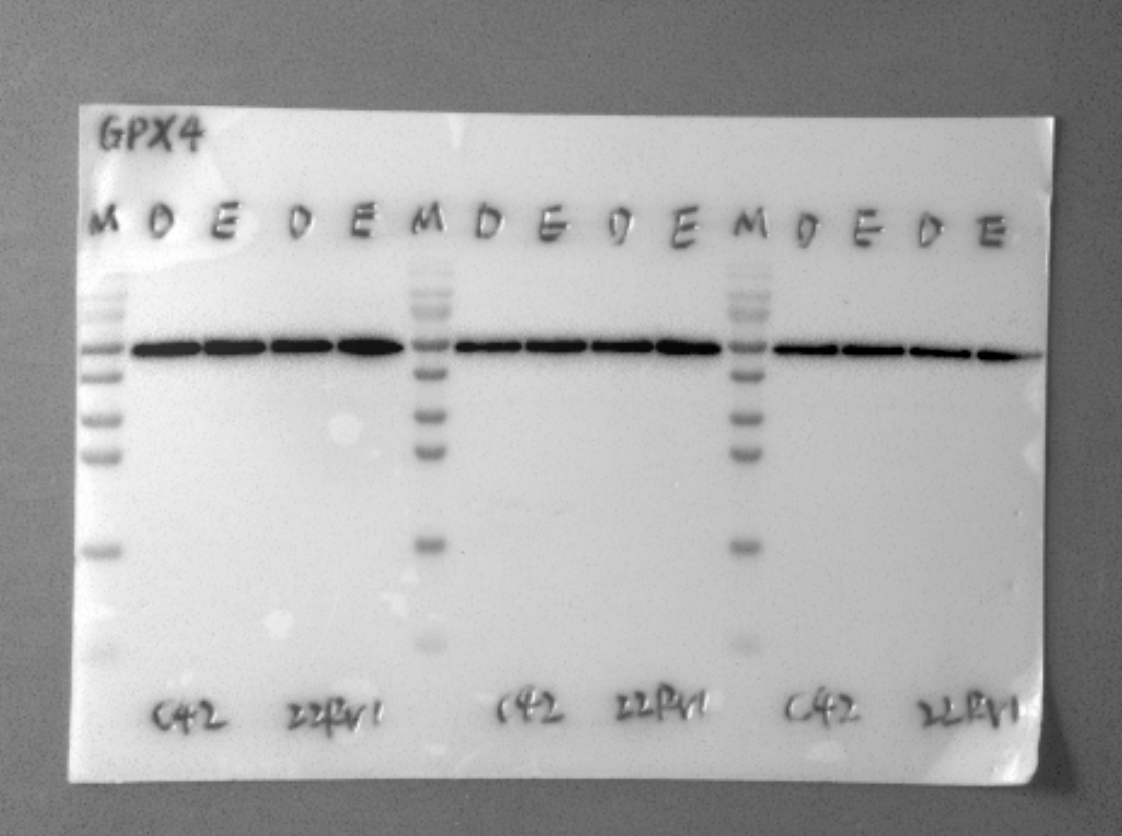

Supplement: Supplementary file 1 — Full and uncropped western blots [file 41419_2025_7809_MOESM1_ESM.zip › Full and uncropped western blots/Fig2A/GPX4/Tubulin-Merge.tif]

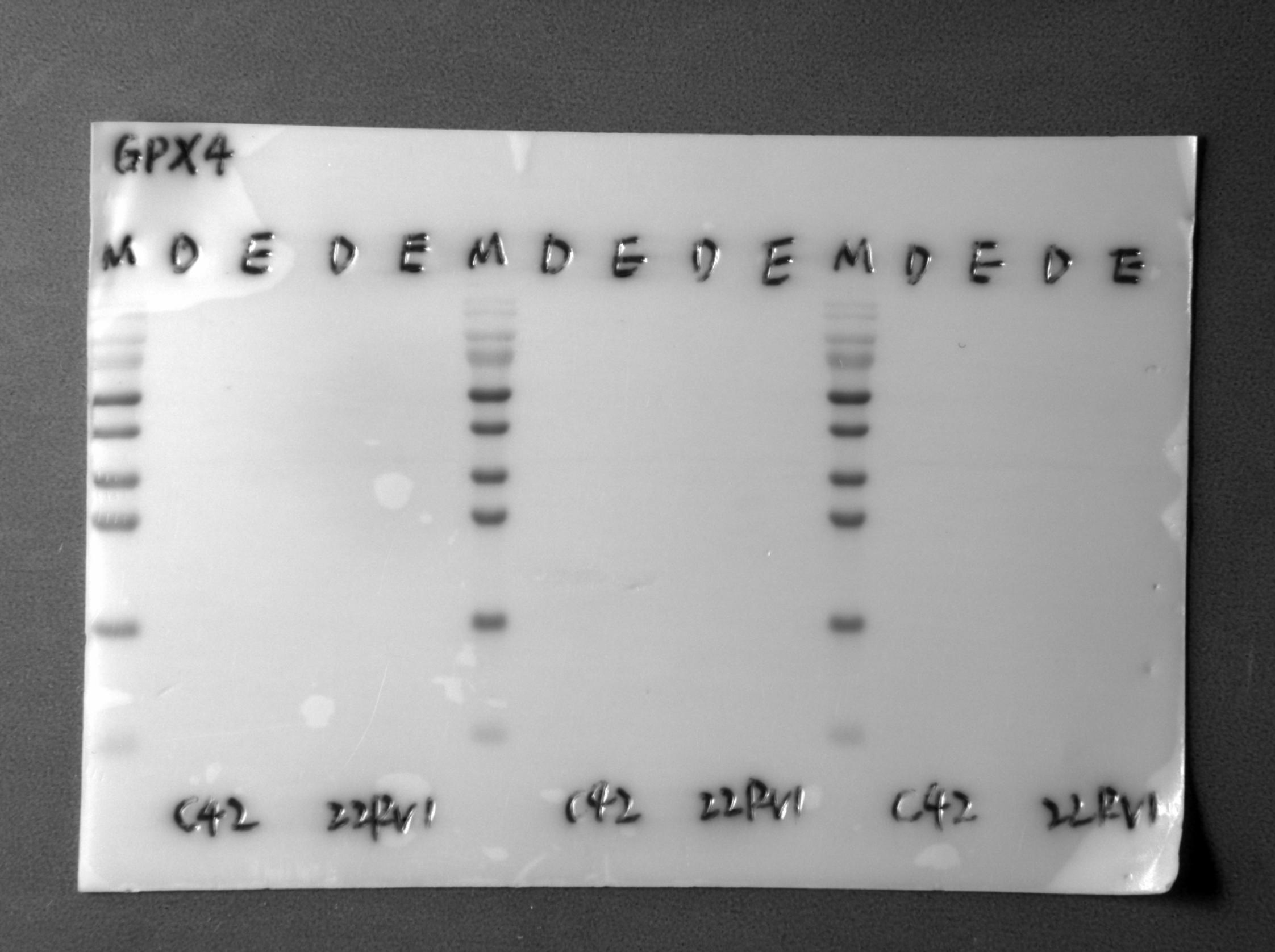

Supplement: Supplementary file 1 — Full and uncropped western blots [file 41419_2025_7809_MOESM1_ESM.zip › Full and uncropped western blots/Fig2A/GPX4/Tubulin-picture of film.tif]

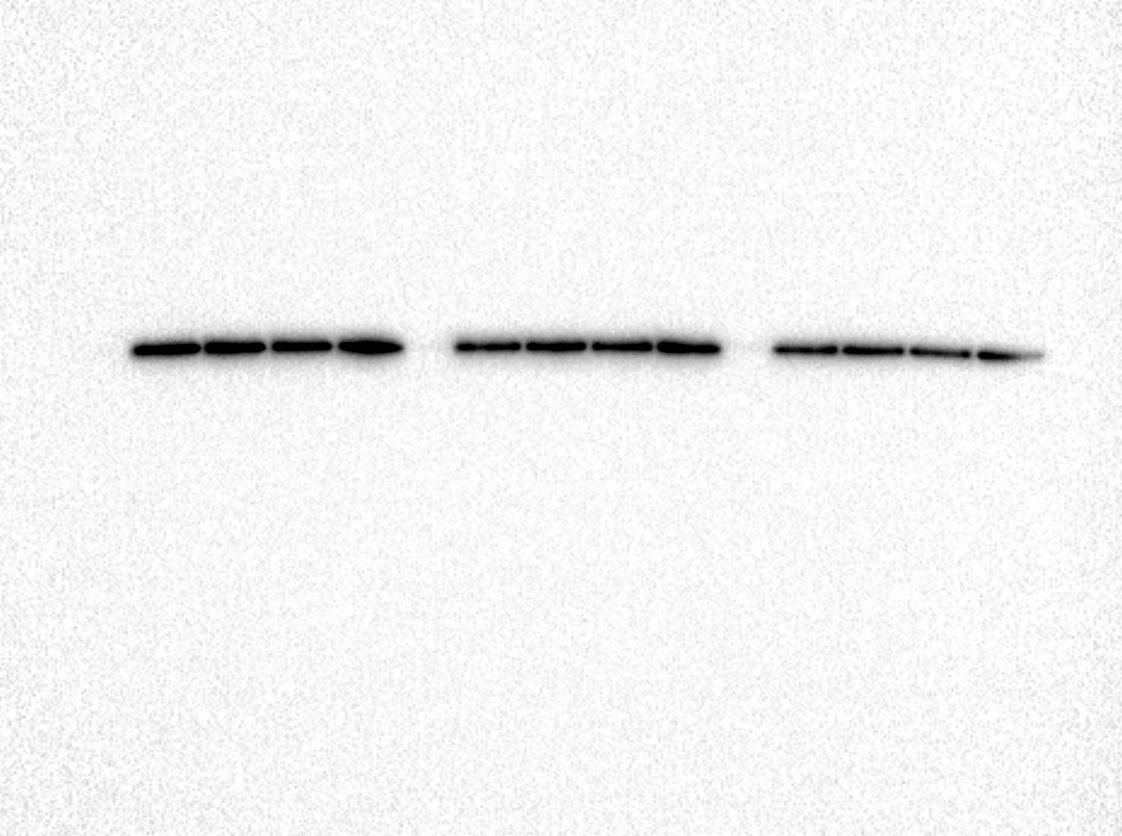

Supplement: Supplementary file 1 — Full and uncropped western blots [file 41419_2025_7809_MOESM1_ESM.zip › Full and uncropped western blots/Fig2A/GPX4/Tubulin.tif]

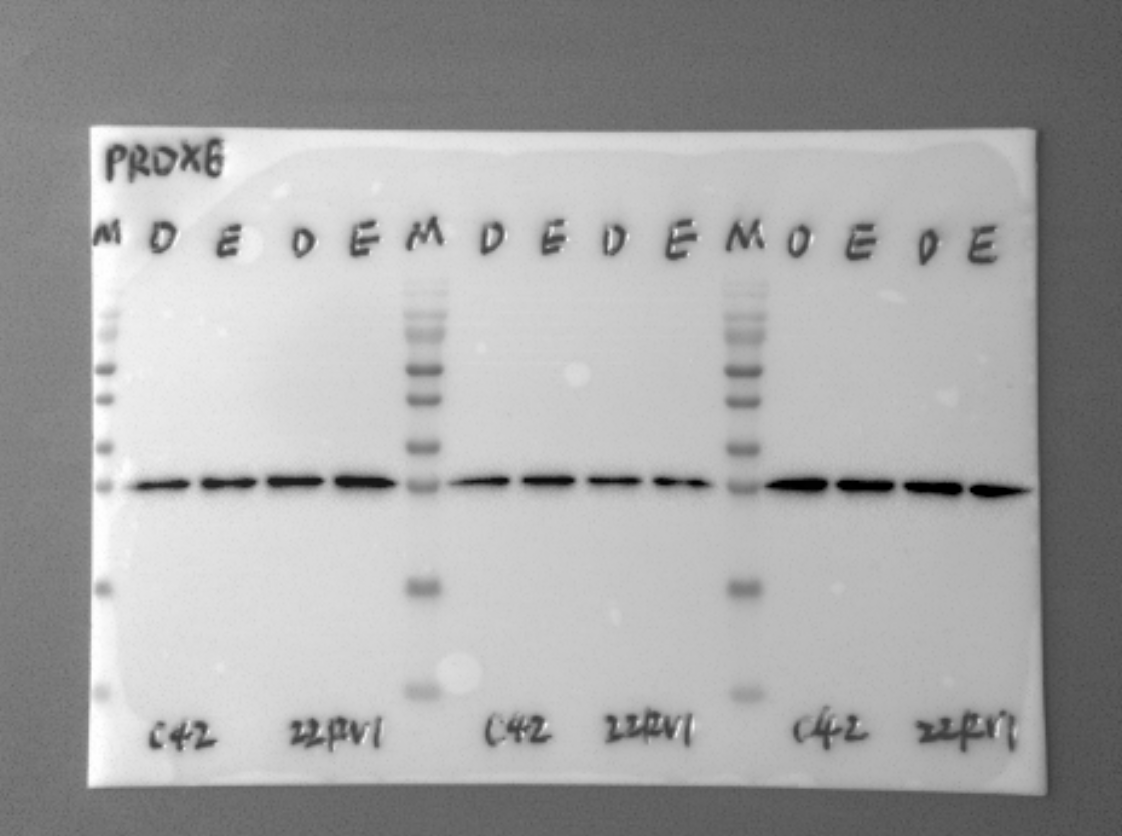

Supplement: Supplementary file 1 — Full and uncropped western blots [file 41419_2025_7809_MOESM1_ESM.zip › Full and uncropped western blots/Fig2A/PRDX6/PRDX6-Merge.tif]

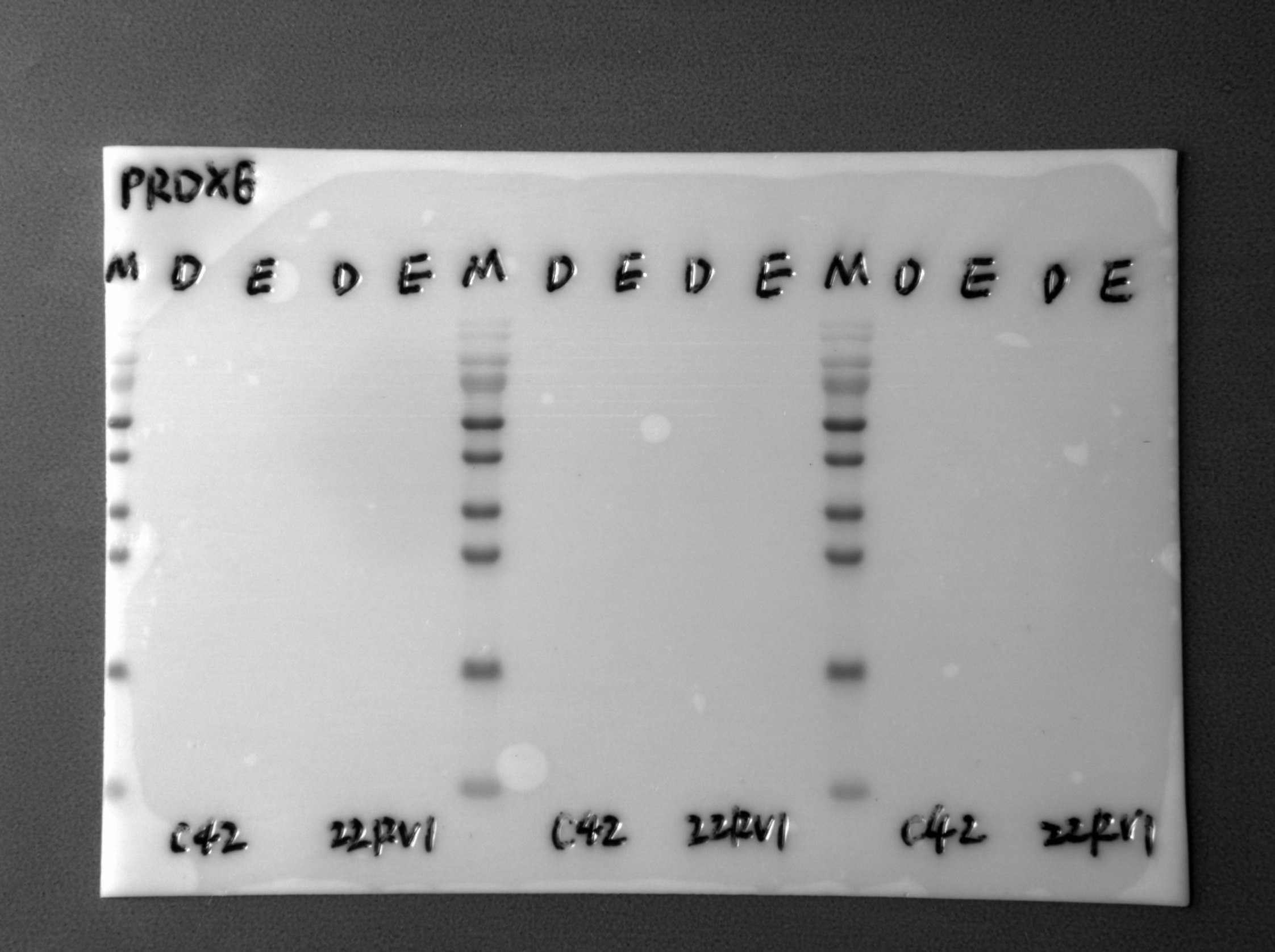

Supplement: Supplementary file 1 — Full and uncropped western blots [file 41419_2025_7809_MOESM1_ESM.zip › Full and uncropped western blots/Fig2A/PRDX6/PRDX6-picture of film.tif]

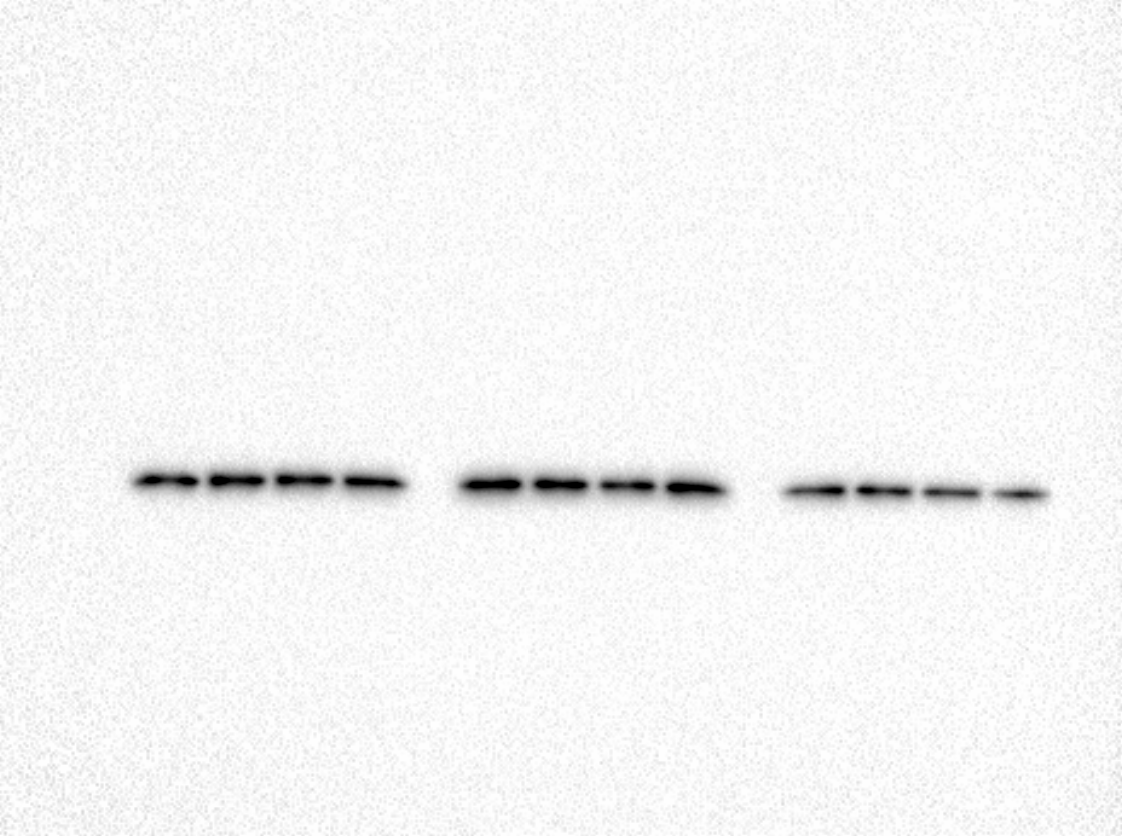

Supplement: Supplementary file 1 — Full and uncropped western blots [file 41419_2025_7809_MOESM1_ESM.zip › Full and uncropped western blots/Fig2A/PRDX6/PRDX6.tif]

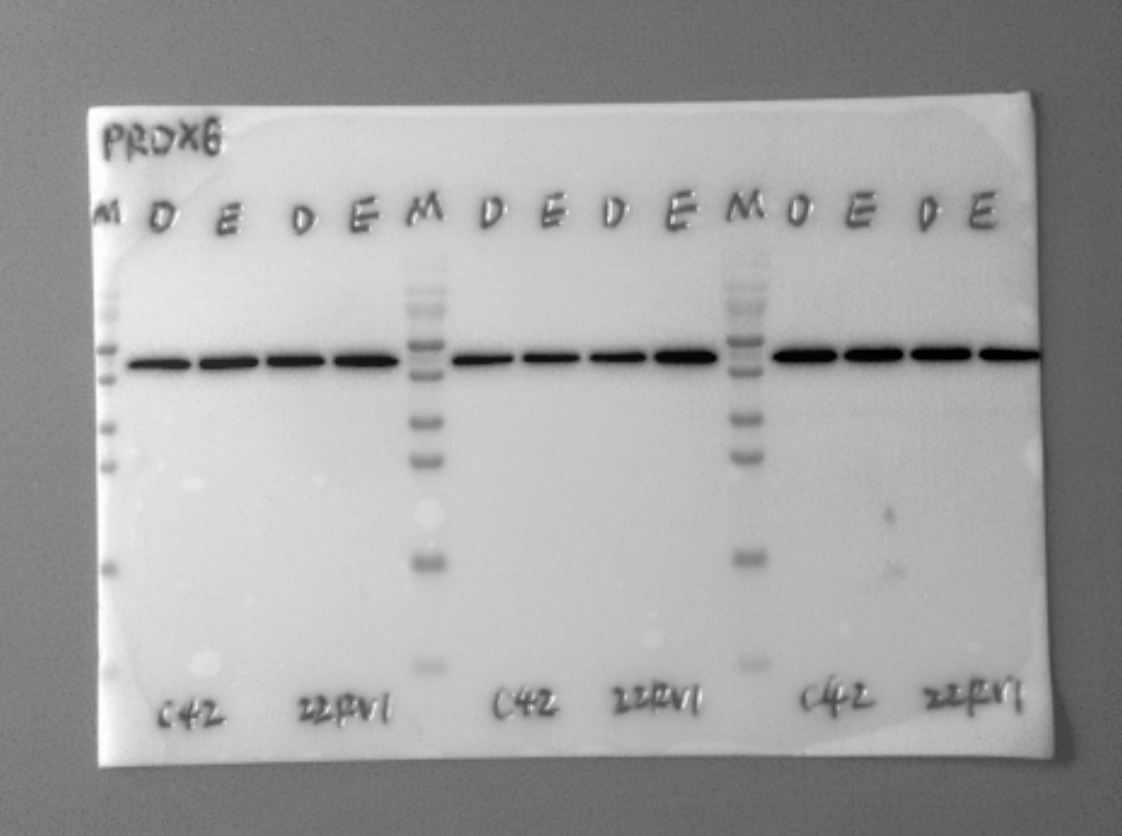

Supplement: Supplementary file 1 — Full and uncropped western blots [file 41419_2025_7809_MOESM1_ESM.zip › Full and uncropped western blots/Fig2A/PRDX6/Tubulin-Merge.tif]

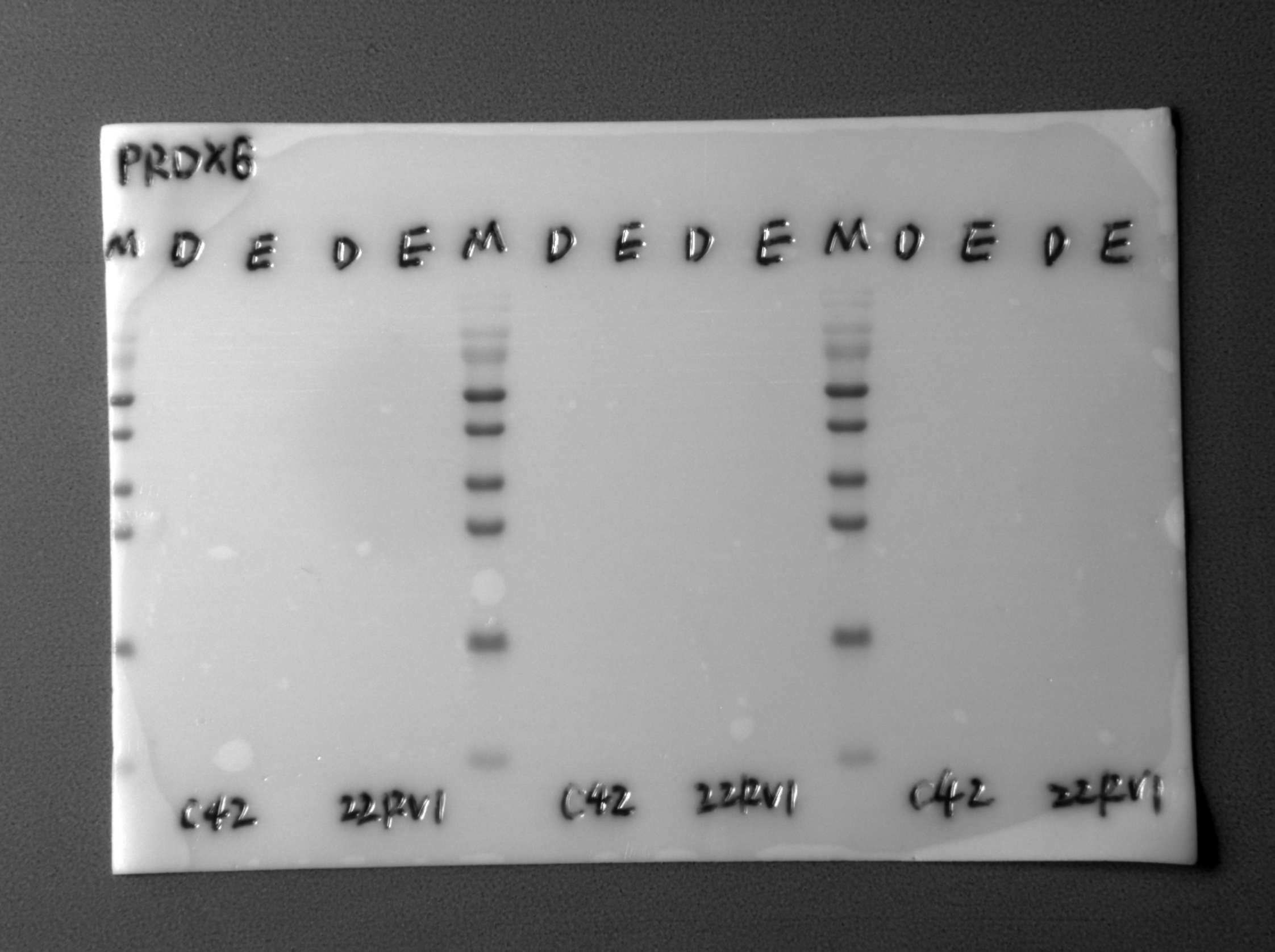

Supplement: Supplementary file 1 — Full and uncropped western blots [file 41419_2025_7809_MOESM1_ESM.zip › Full and uncropped western blots/Fig2A/PRDX6/Tubulin-picture of film.tif]

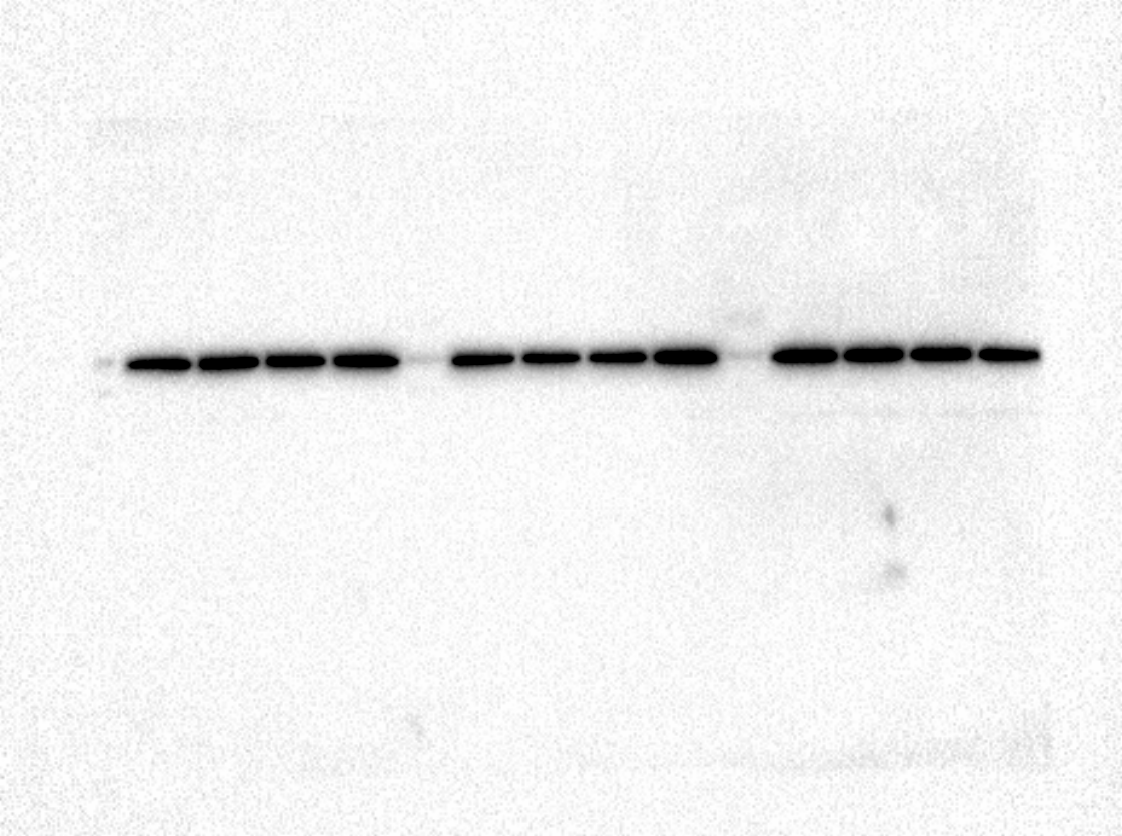

Supplement: Supplementary file 1 — Full and uncropped western blots [file 41419_2025_7809_MOESM1_ESM.zip › Full and uncropped western blots/Fig2A/PRDX6/Tubulin.tif]

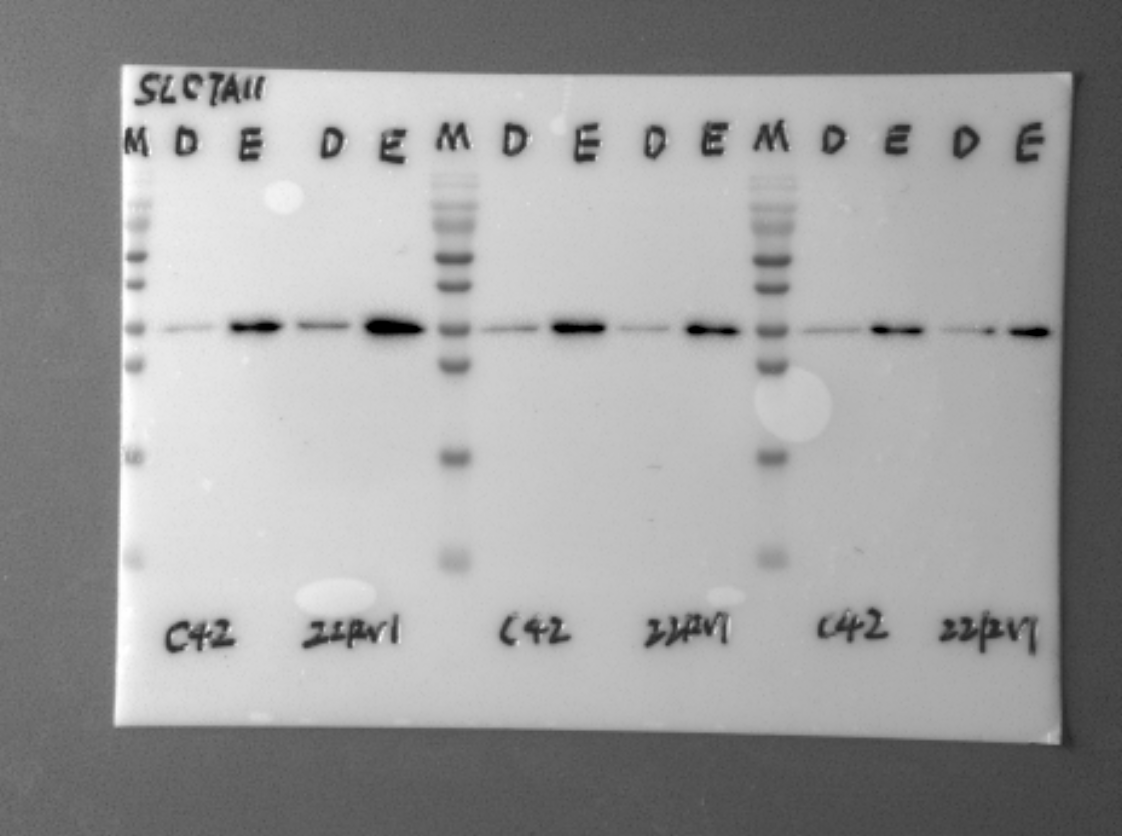

Supplement: Supplementary file 1 — Full and uncropped western blots [file 41419_2025_7809_MOESM1_ESM.zip › Full and uncropped western blots/Fig2A/SLC7A11/SLC7A11-Merge.tif]

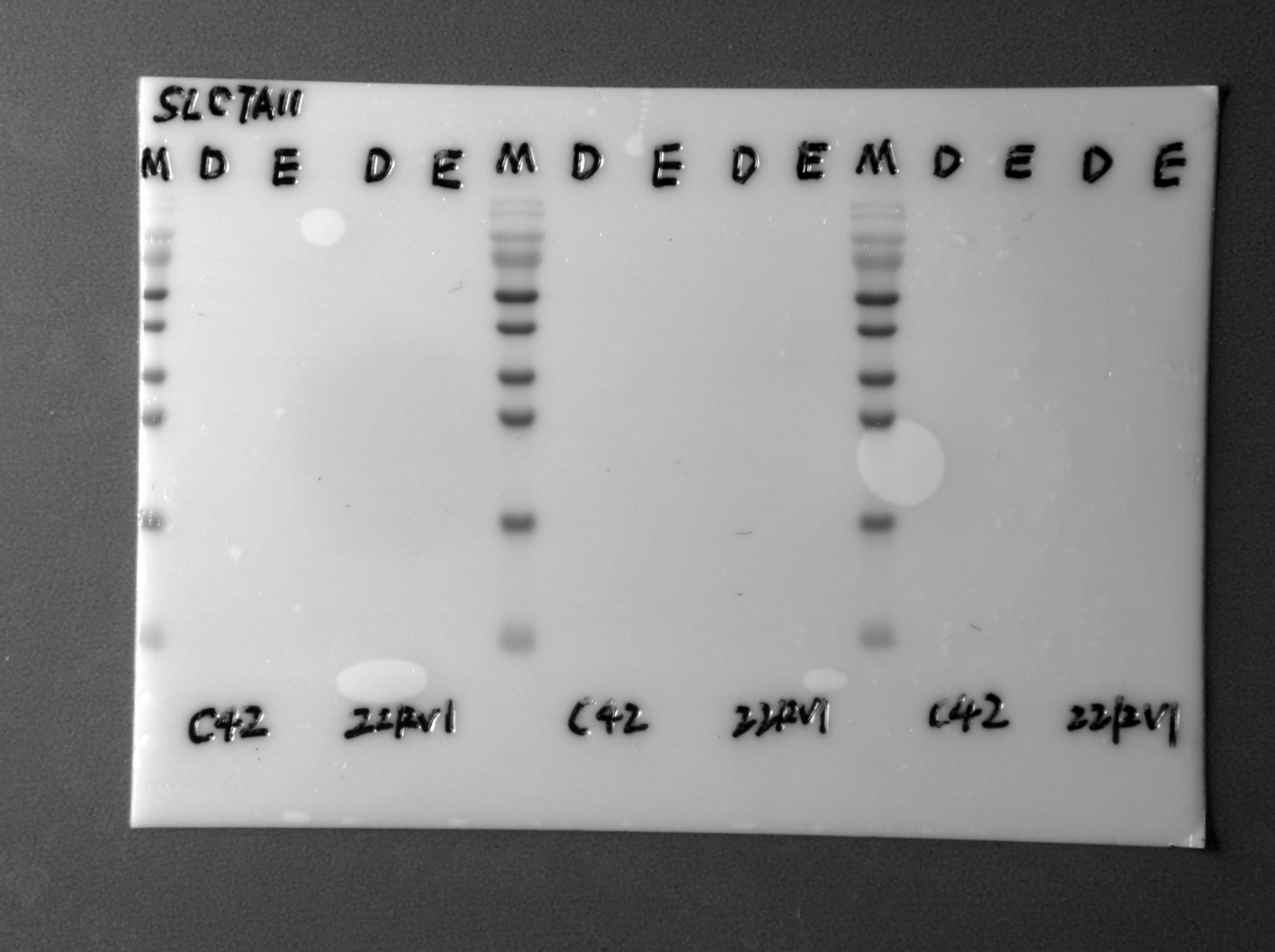

Supplement: Supplementary file 1 — Full and uncropped western blots [file 41419_2025_7809_MOESM1_ESM.zip › Full and uncropped western blots/Fig2A/SLC7A11/SLC7A11-picture of film.tif]

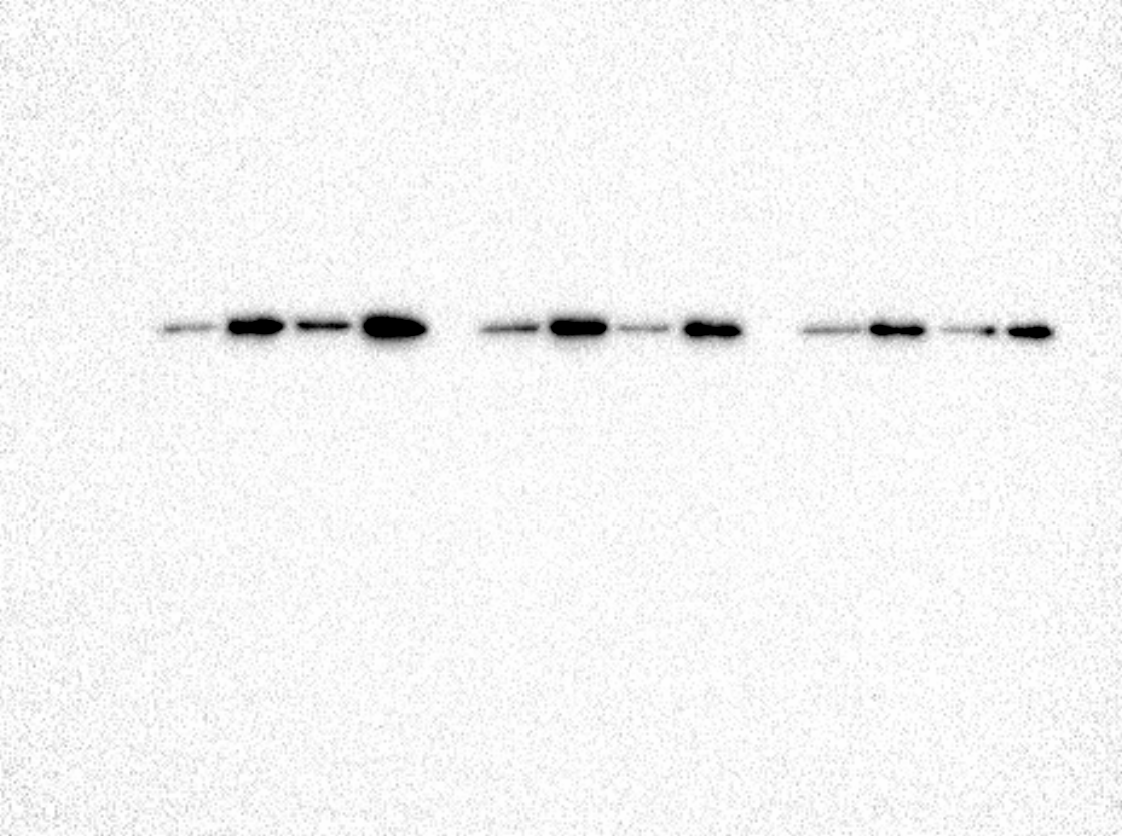

Supplement: Supplementary file 1 — Full and uncropped western blots [file 41419_2025_7809_MOESM1_ESM.zip › Full and uncropped western blots/Fig2A/SLC7A11/SLC7A11.tif]

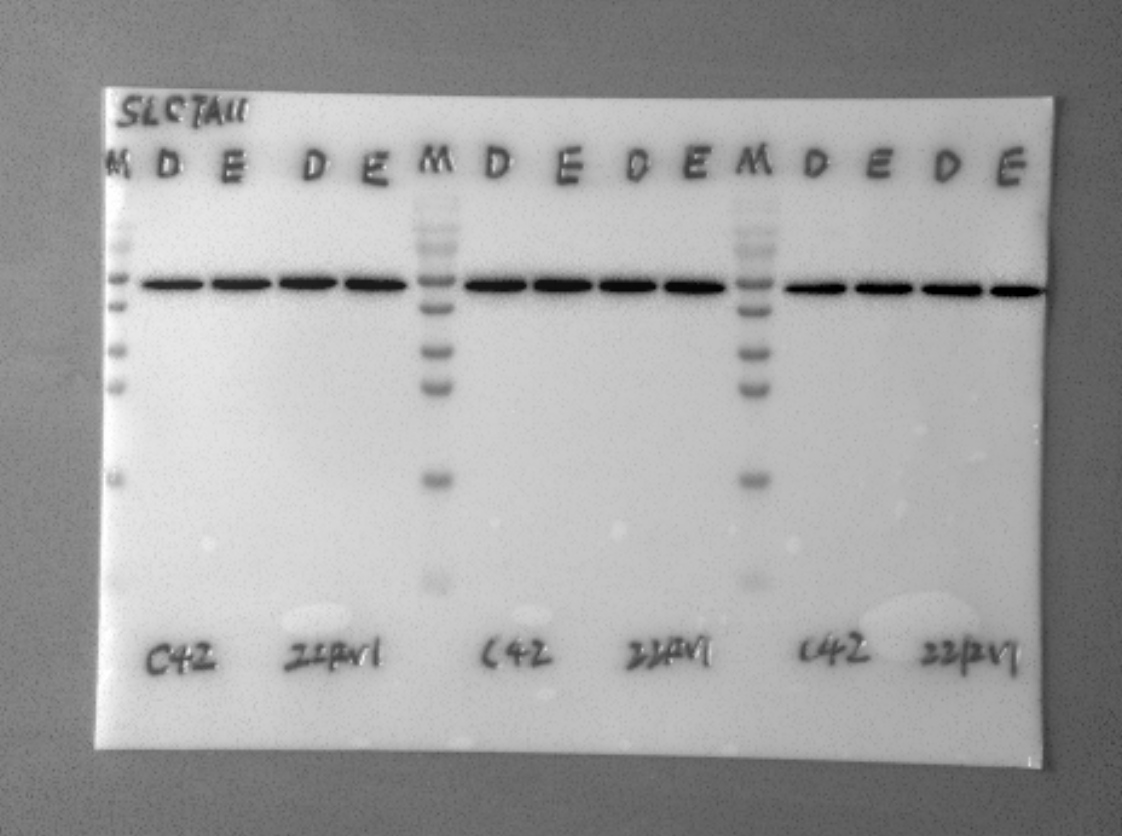

Supplement: Supplementary file 1 — Full and uncropped western blots [file 41419_2025_7809_MOESM1_ESM.zip › Full and uncropped western blots/Fig2A/SLC7A11/Tubulin-Merge.tif]

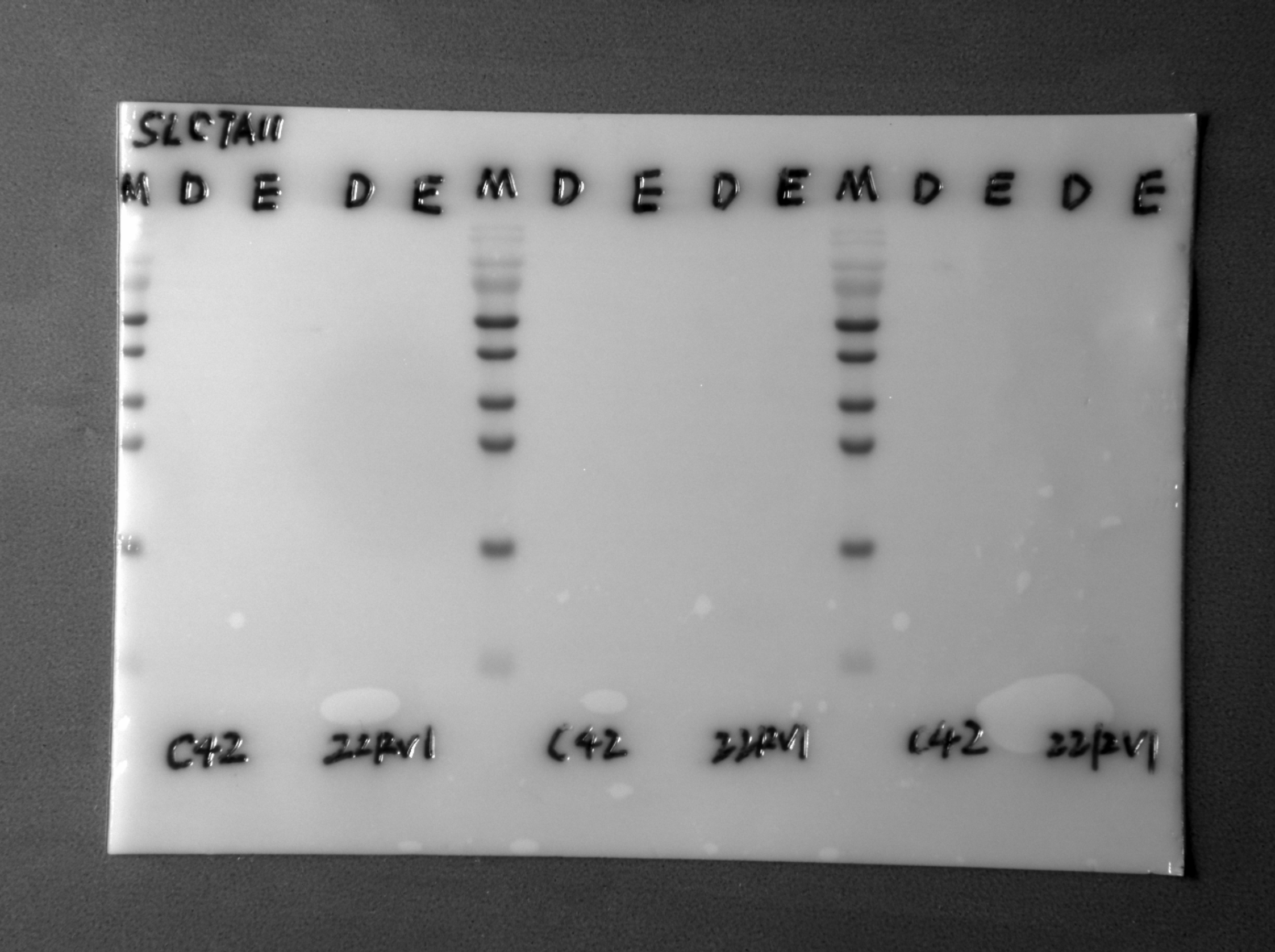

Supplement: Supplementary file 1 — Full and uncropped western blots [file 41419_2025_7809_MOESM1_ESM.zip › Full and uncropped western blots/Fig2A/SLC7A11/Tubulin-picture of film.tif]

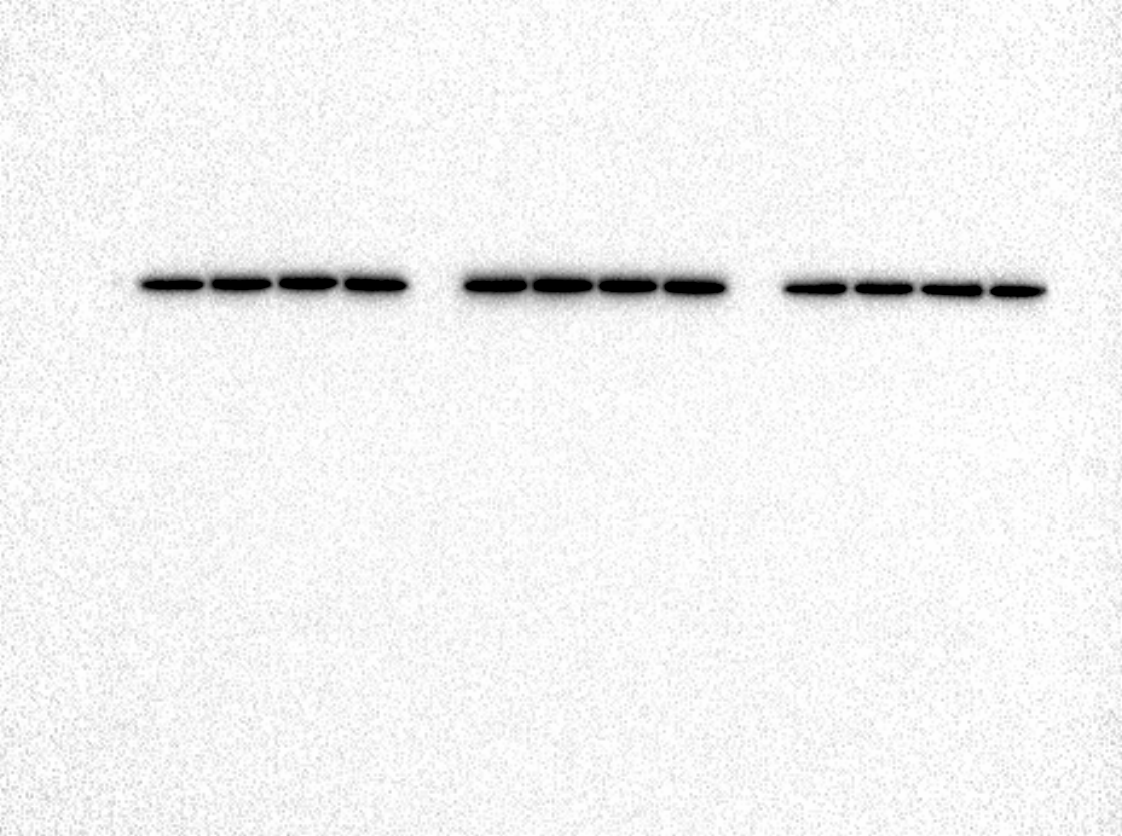

Supplement: Supplementary file 1 — Full and uncropped western blots [file 41419_2025_7809_MOESM1_ESM.zip › Full and uncropped western blots/Fig2A/SLC7A11/Tubulin.tif]

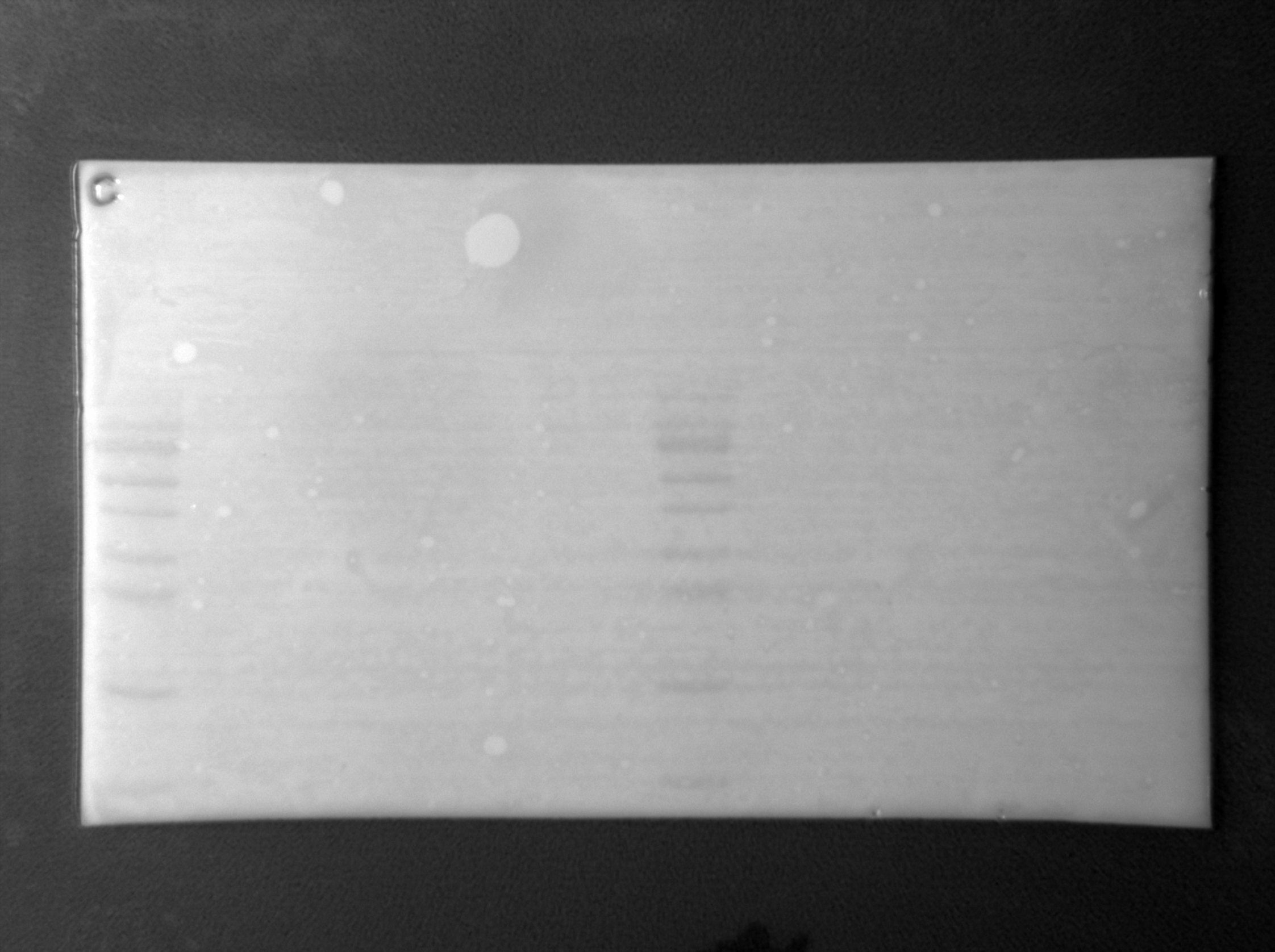

Supplement: Supplementary file 1 — Full and uncropped western blots [file 41419_2025_7809_MOESM1_ESM.zip › Full and uncropped western blots/Fig2B/AR film 1 -picture of film.tif]

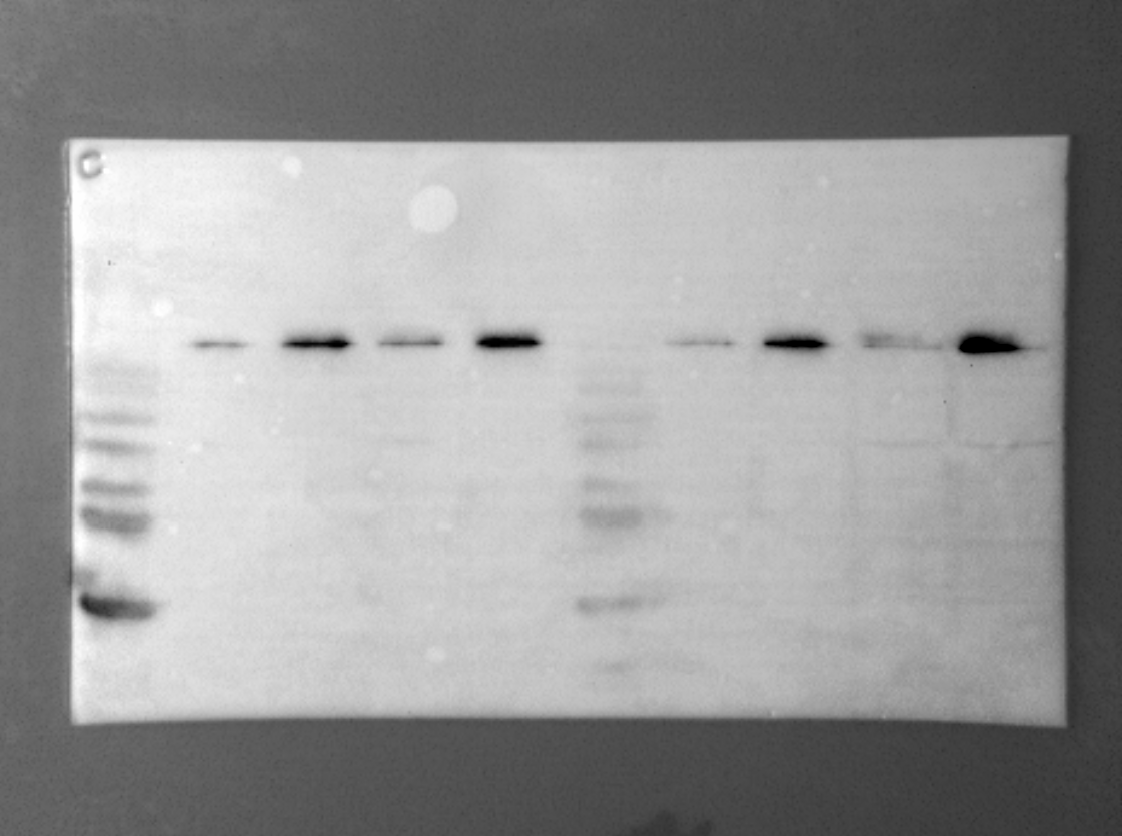

Supplement: Supplementary file 1 — Full and uncropped western blots [file 41419_2025_7809_MOESM1_ESM.zip › Full and uncropped western blots/Fig2B/AR film 1 Merge.tif]

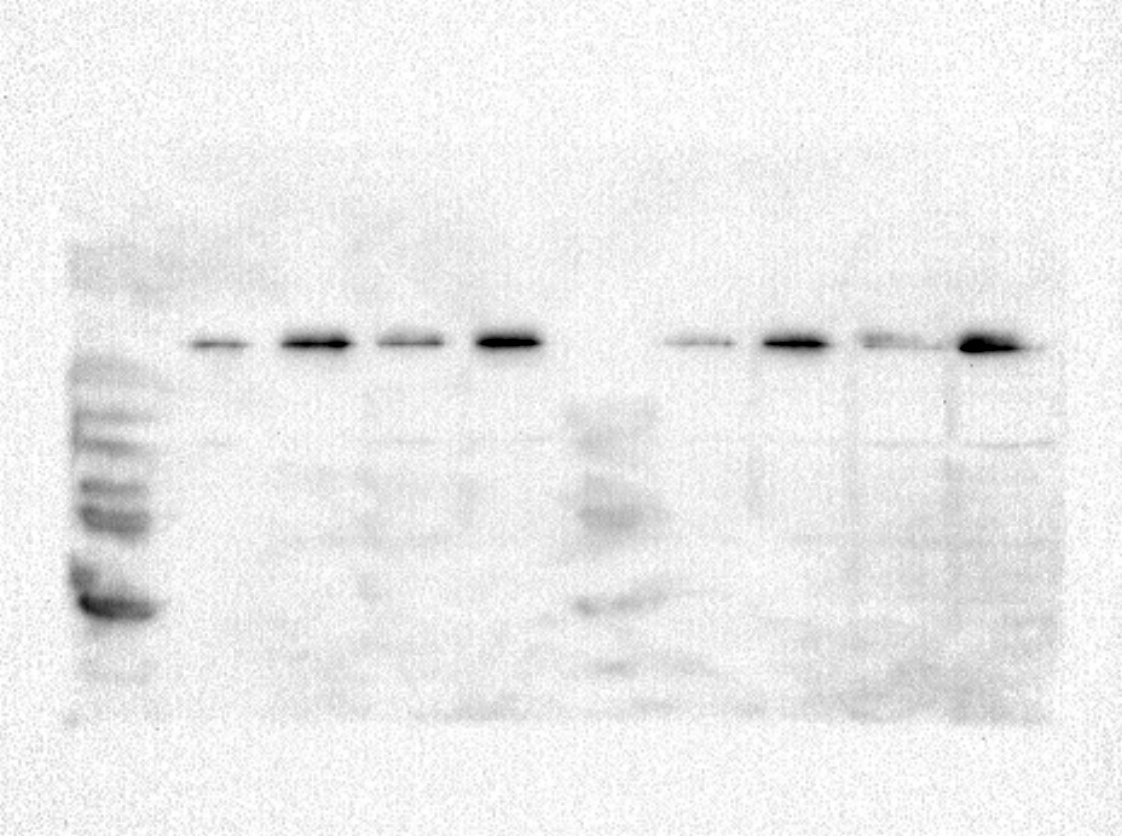

Supplement: Supplementary file 1 — Full and uncropped western blots [file 41419_2025_7809_MOESM1_ESM.zip › Full and uncropped western blots/Fig2B/AR film 1.tif]

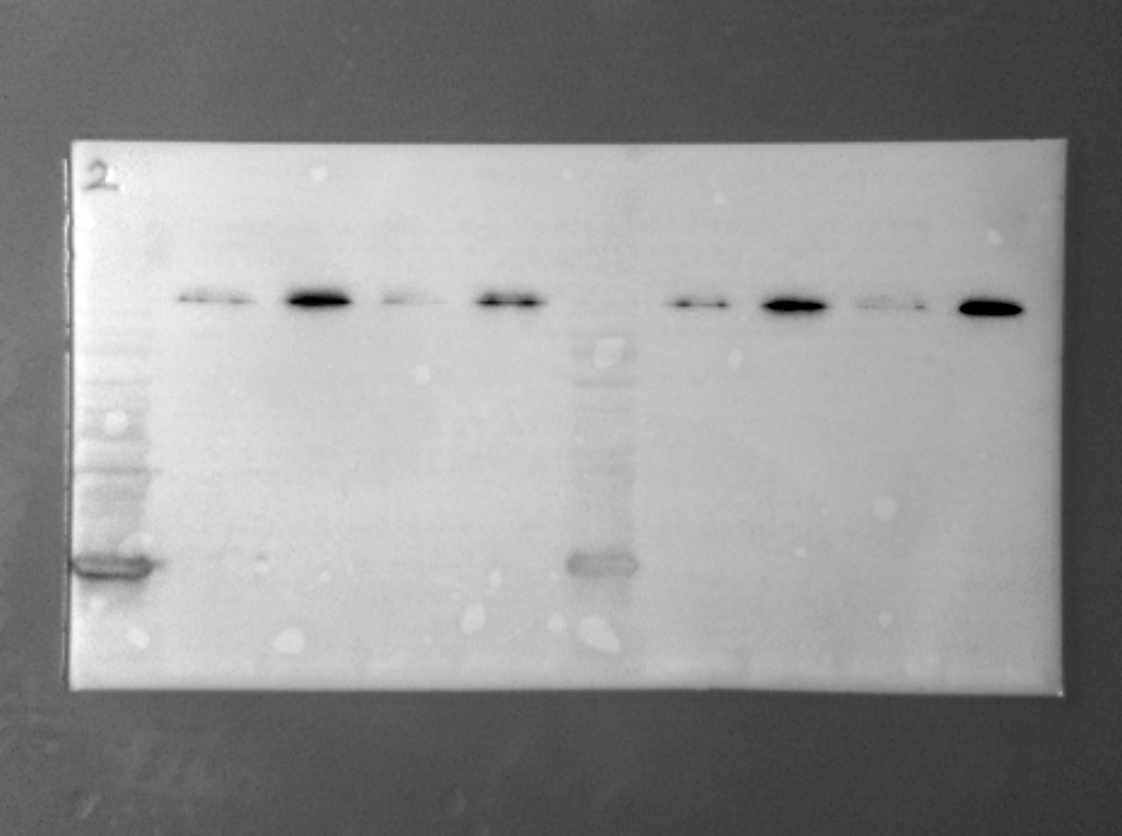

Supplement: Supplementary file 1 — Full and uncropped western blots [file 41419_2025_7809_MOESM1_ESM.zip › Full and uncropped western blots/Fig2B/AR film 2 Merge.tif]

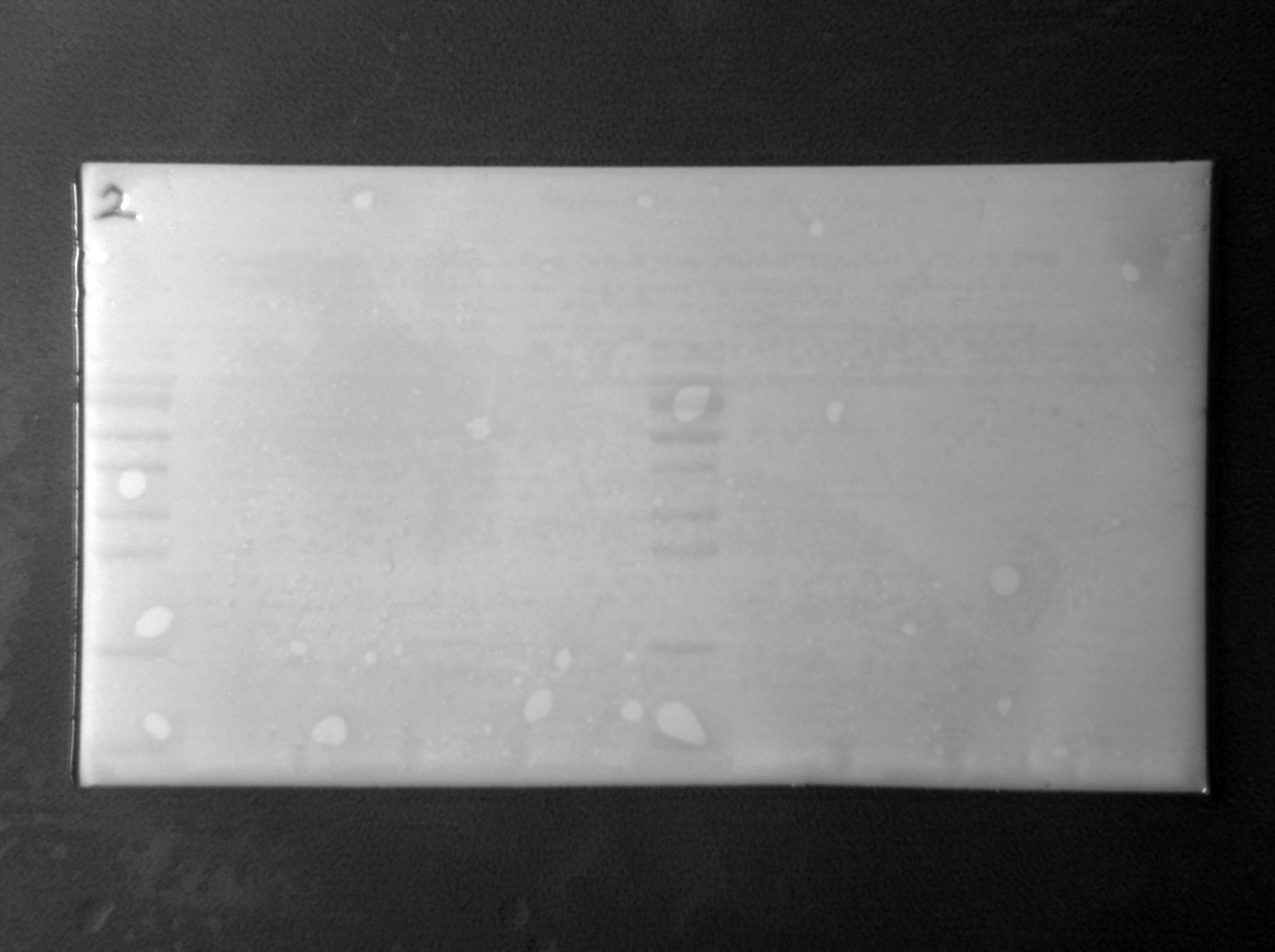

Supplement: Supplementary file 1 — Full and uncropped western blots [file 41419_2025_7809_MOESM1_ESM.zip › Full and uncropped western blots/Fig2B/AR film 2-picture of film.tif]

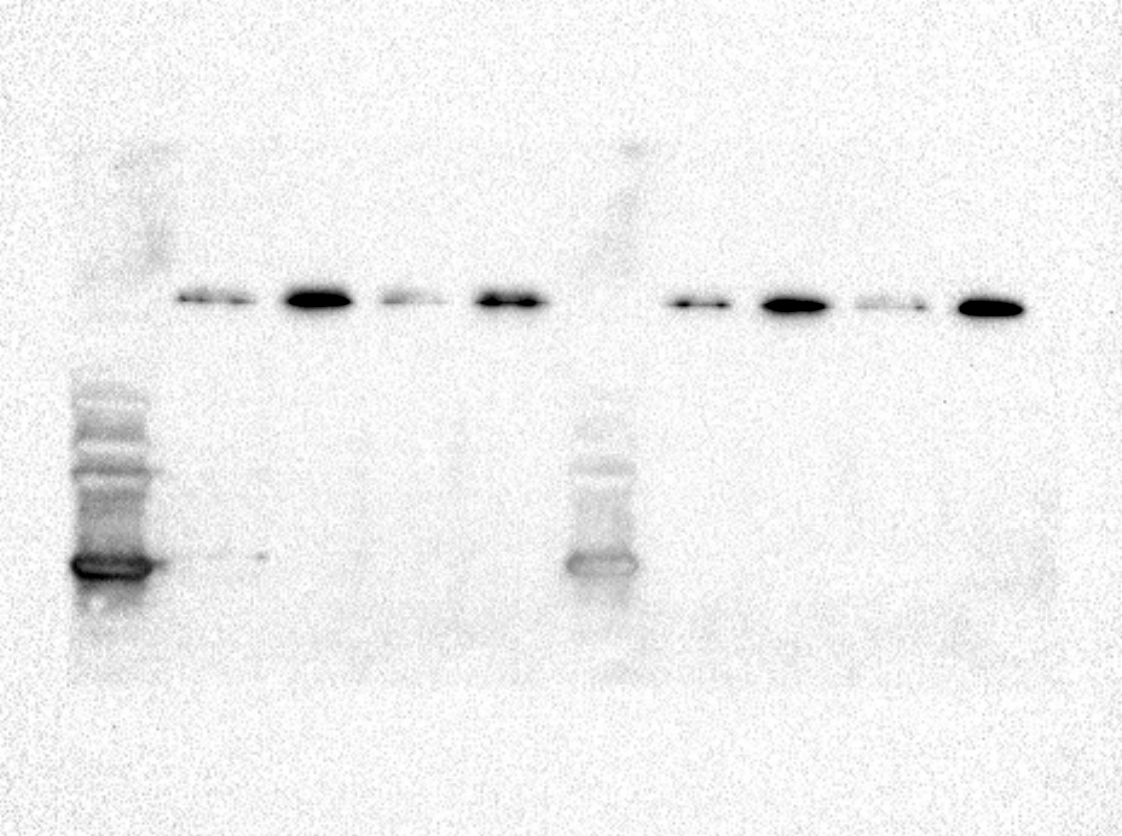

Supplement: Supplementary file 1 — Full and uncropped western blots [file 41419_2025_7809_MOESM1_ESM.zip › Full and uncropped western blots/Fig2B/AR film 2.tif]

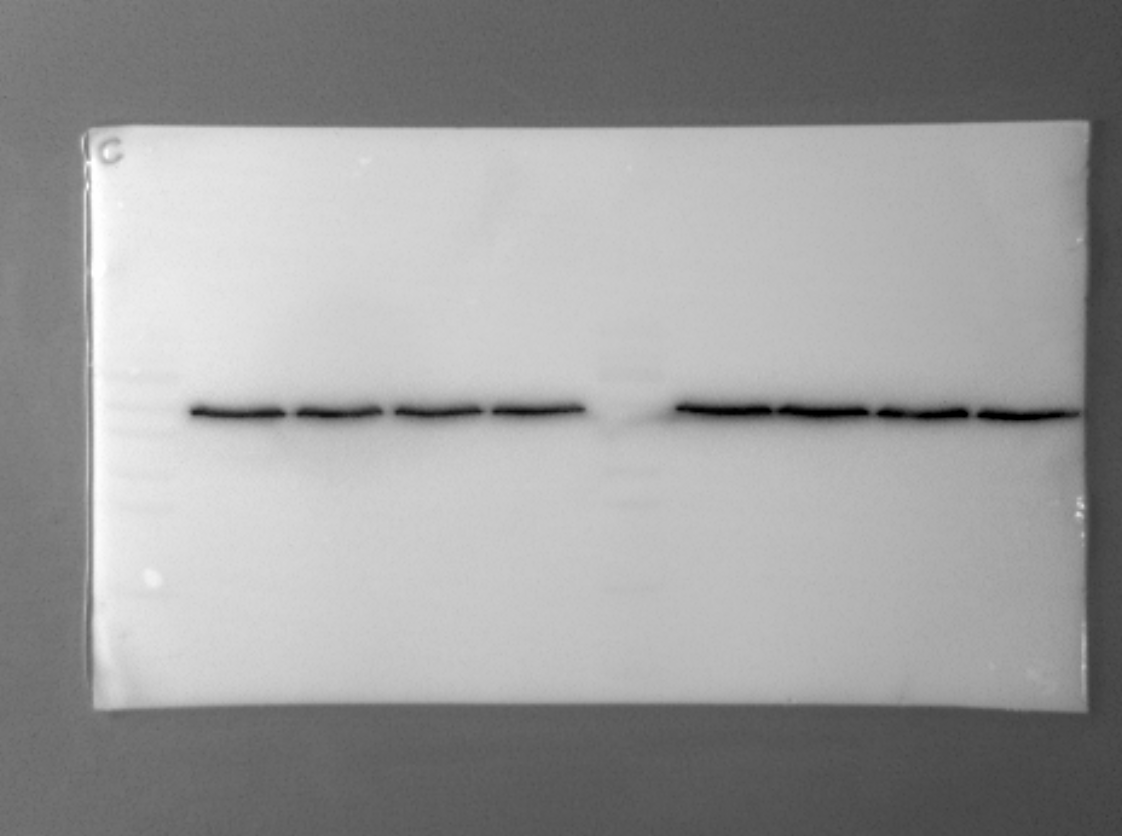

Supplement: Supplementary file 1 — Full and uncropped western blots [file 41419_2025_7809_MOESM1_ESM.zip › Full and uncropped western blots/Fig2B/Tubulin film 1 Merge.tif]

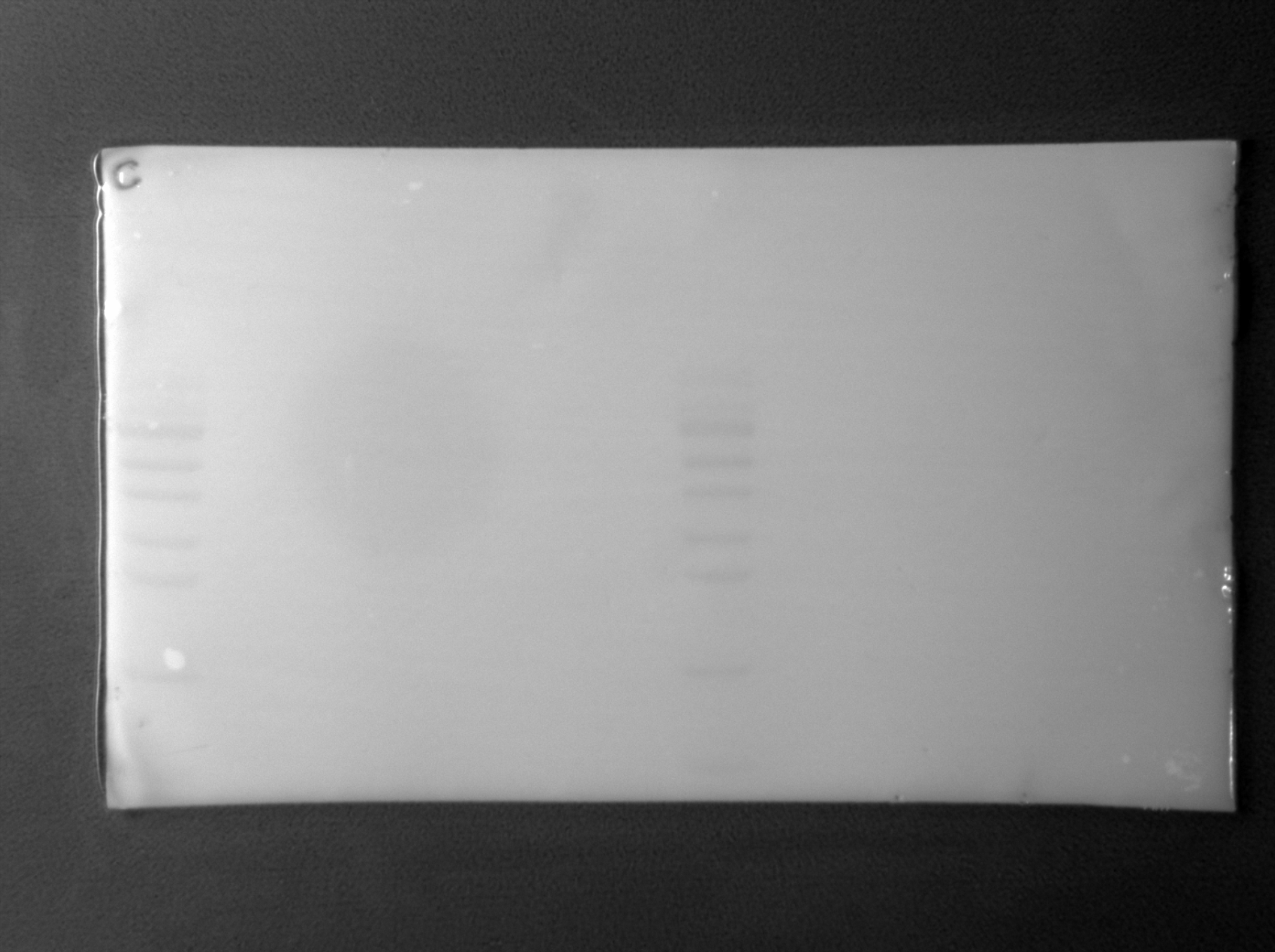

Supplement: Supplementary file 1 — Full and uncropped western blots [file 41419_2025_7809_MOESM1_ESM.zip › Full and uncropped western blots/Fig2B/Tubulin film 1-picture of film.tif]

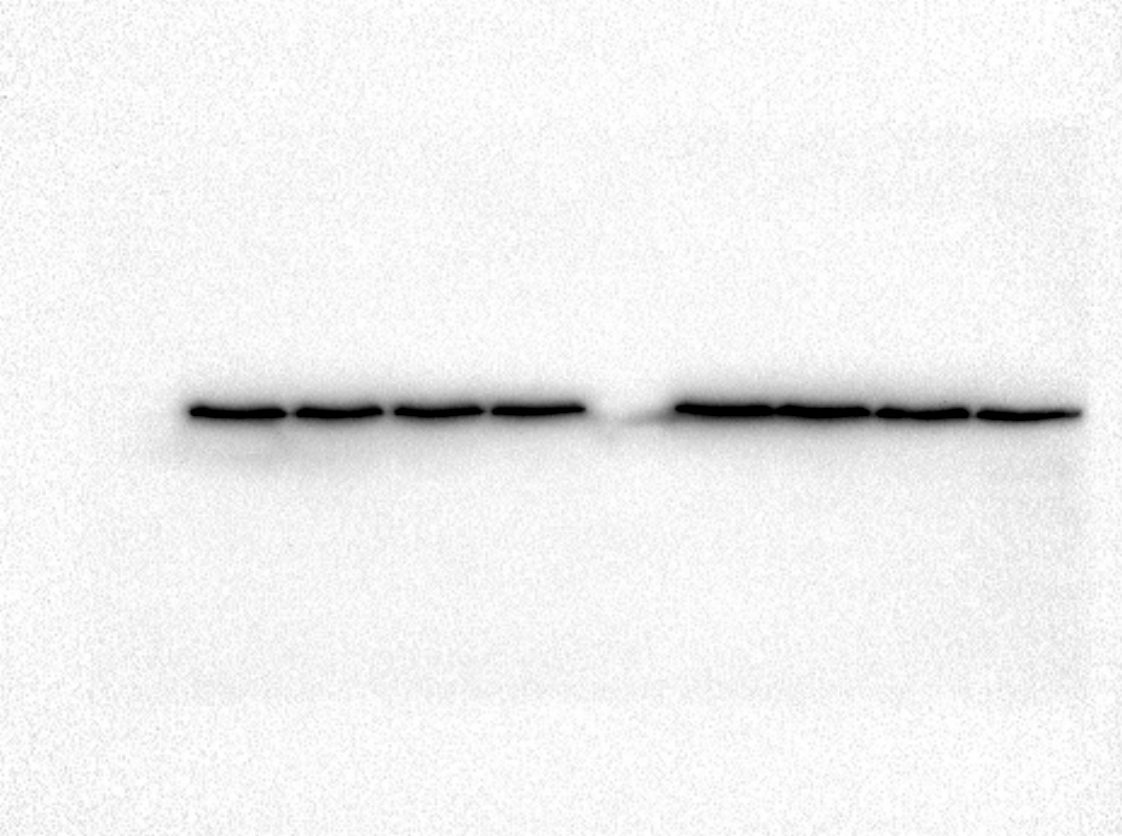

Supplement: Supplementary file 1 — Full and uncropped western blots [file 41419_2025_7809_MOESM1_ESM.zip › Full and uncropped western blots/Fig2B/Tubulin film 1.tif]

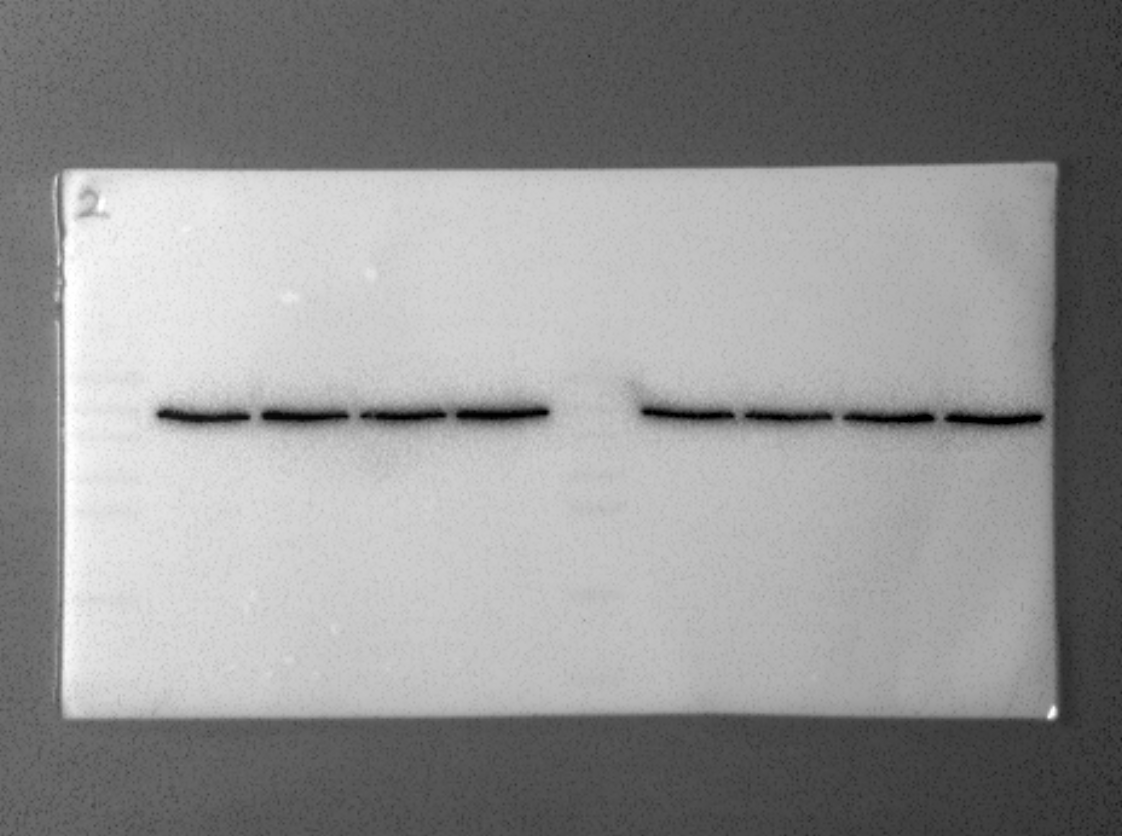

Supplement: Supplementary file 1 — Full and uncropped western blots [file 41419_2025_7809_MOESM1_ESM.zip › Full and uncropped western blots/Fig2B/Tubulin film 2 Merge.tif]

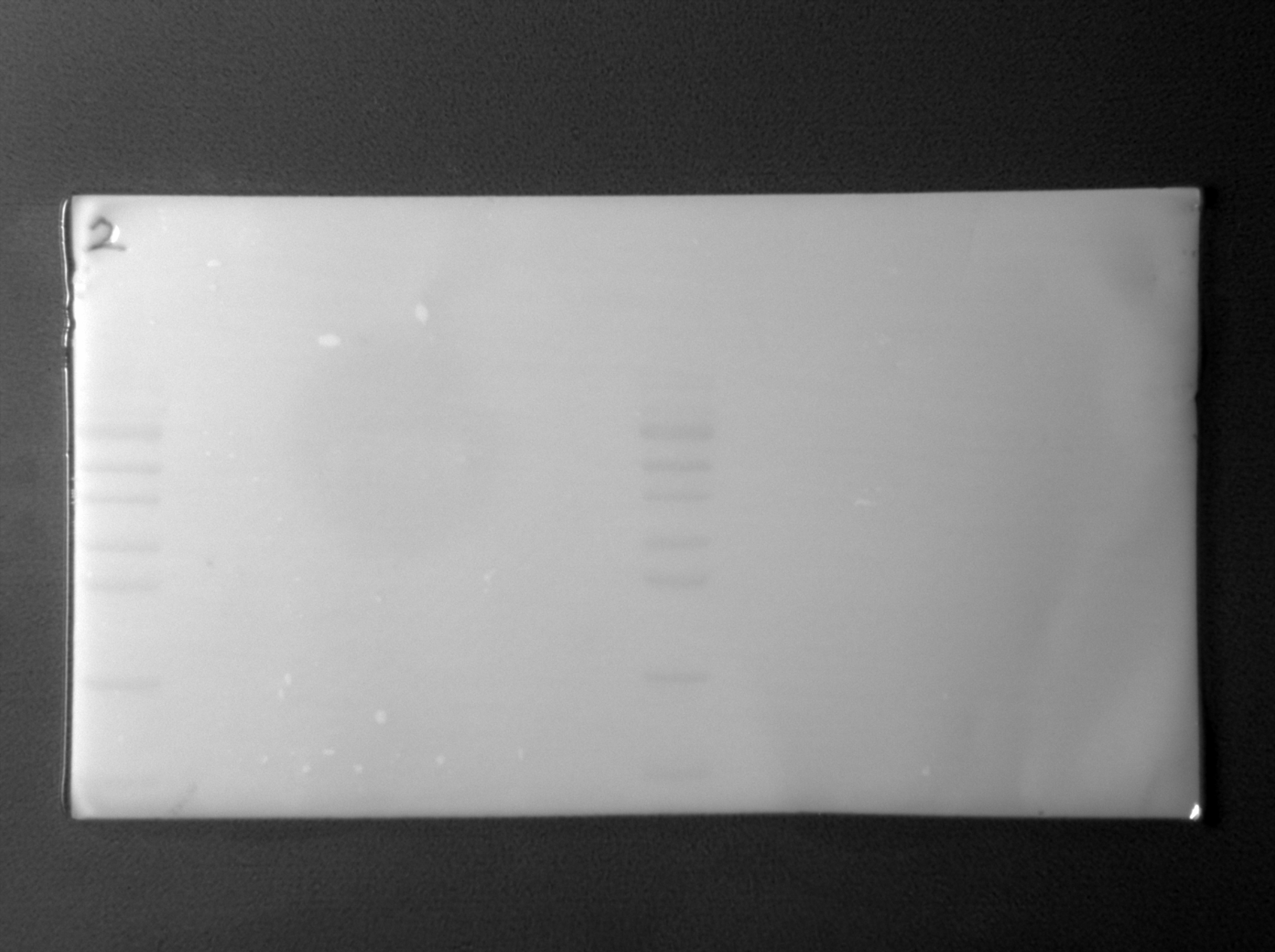

Supplement: Supplementary file 1 — Full and uncropped western blots [file 41419_2025_7809_MOESM1_ESM.zip › Full and uncropped western blots/Fig2B/Tubulin film 2-picture of film.tif]

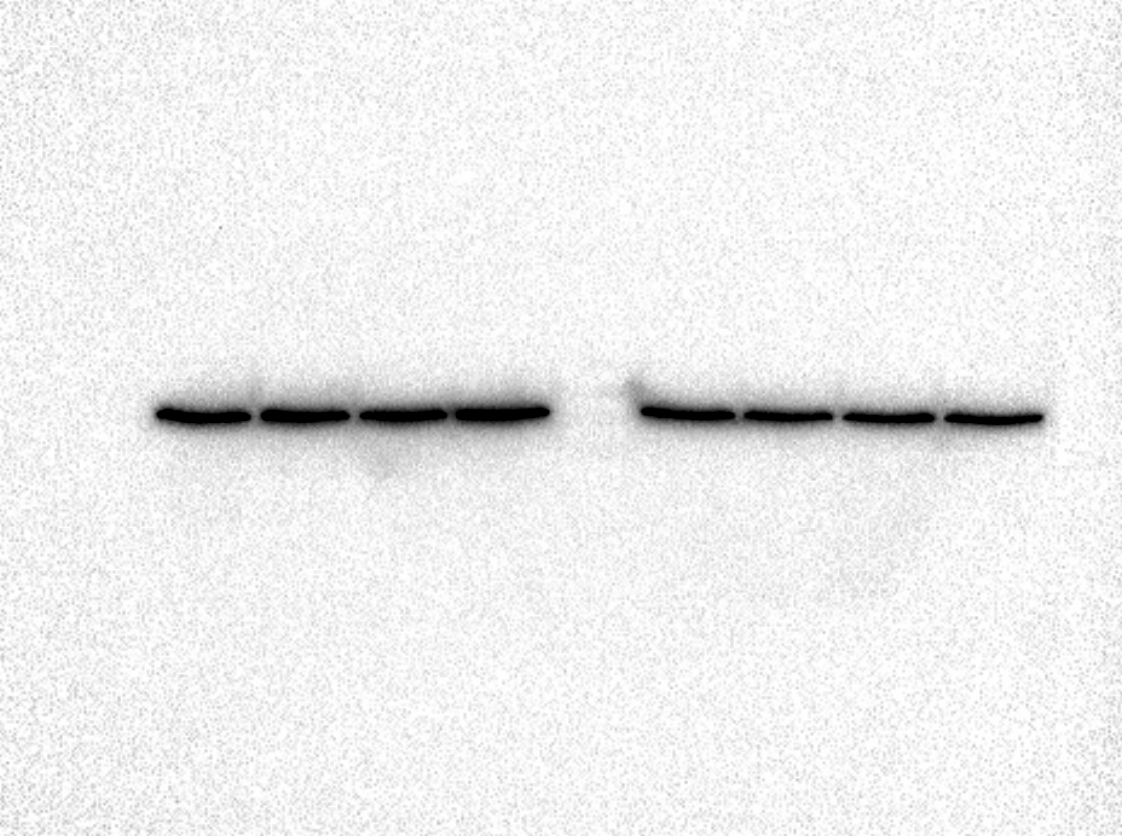

Supplement: Supplementary file 1 — Full and uncropped western blots [file 41419_2025_7809_MOESM1_ESM.zip › Full and uncropped western blots/Fig2B/Tubulin film 2.tif]

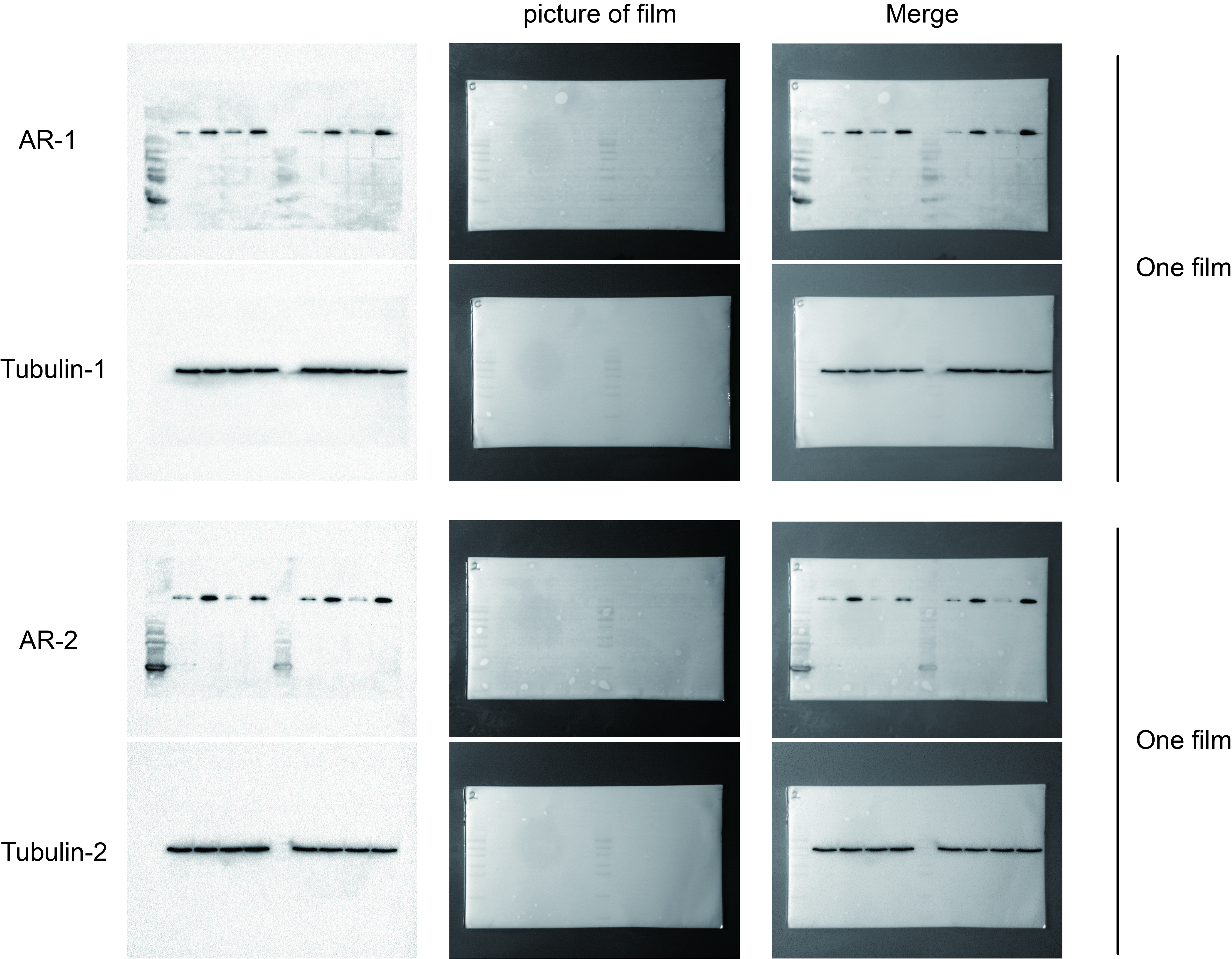

Supplement: Supplementary file 1 — Full and uncropped western blots [file 41419_2025_7809_MOESM1_ESM.zip › Full and uncropped western blots/Fig2B.tif]

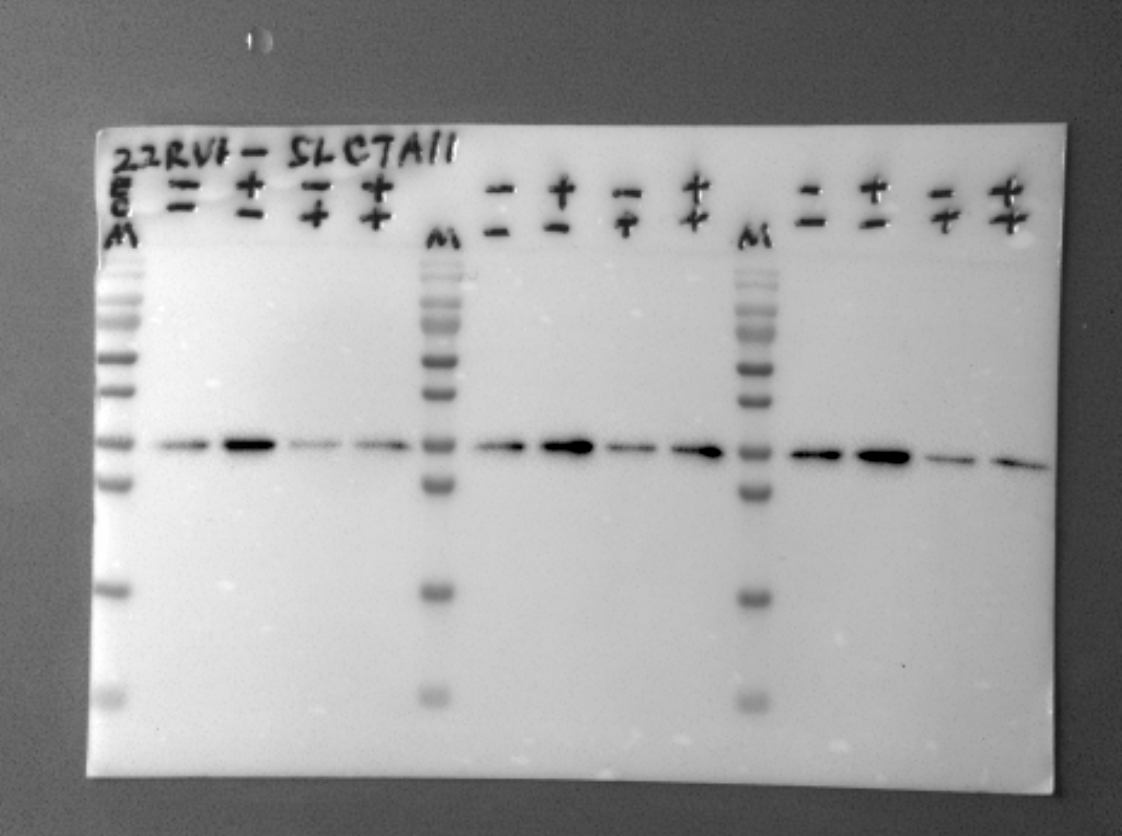

Supplement: Supplementary file 1 — Full and uncropped western blots [file 41419_2025_7809_MOESM1_ESM.zip › Full and uncropped western blots/Fig2C/22RV1/SLC7A11-Merge.tif]

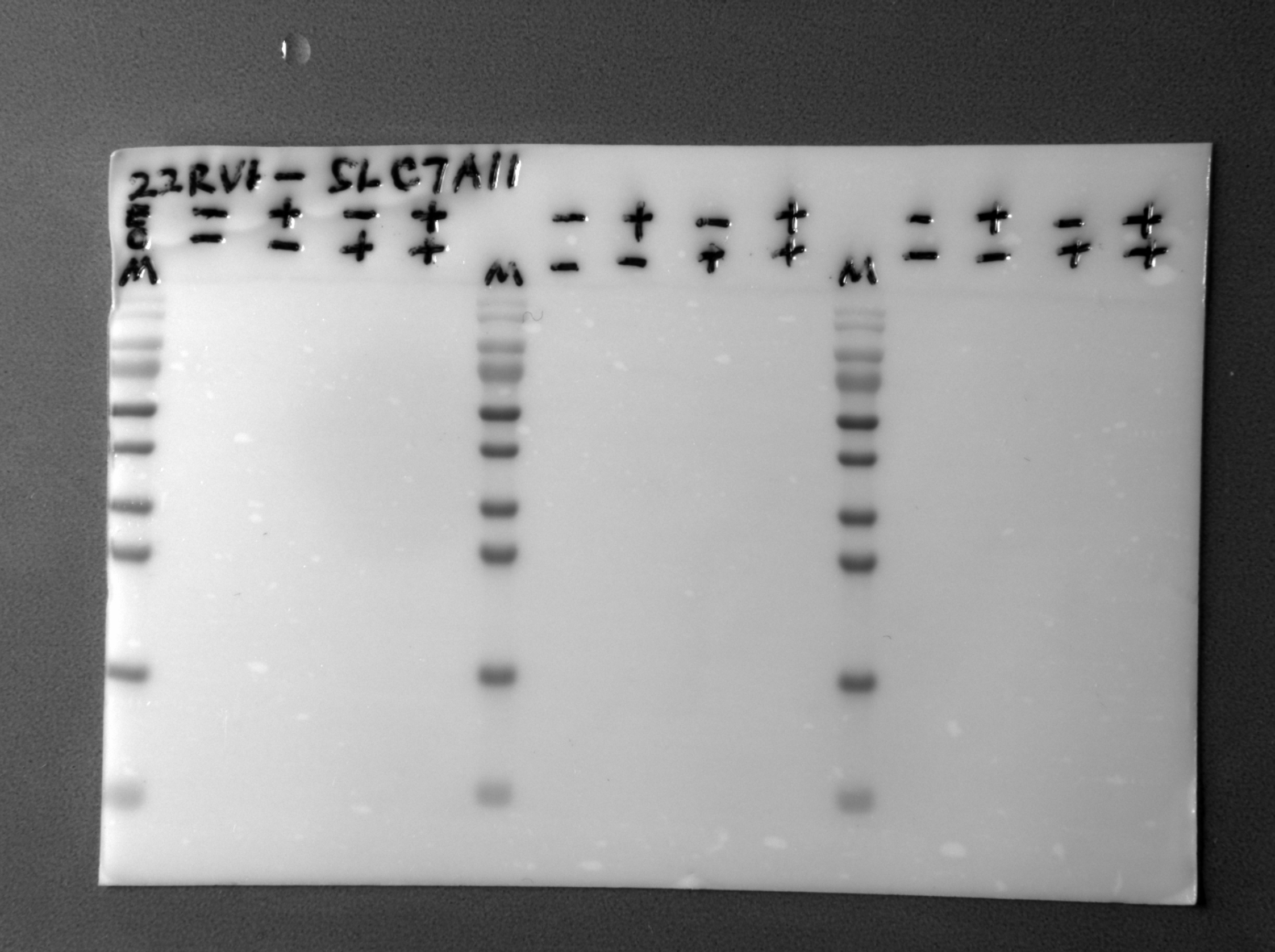

Supplement: Supplementary file 1 — Full and uncropped western blots [file 41419_2025_7809_MOESM1_ESM.zip › Full and uncropped western blots/Fig2C/22RV1/SLC7A11-picture of film.tif]

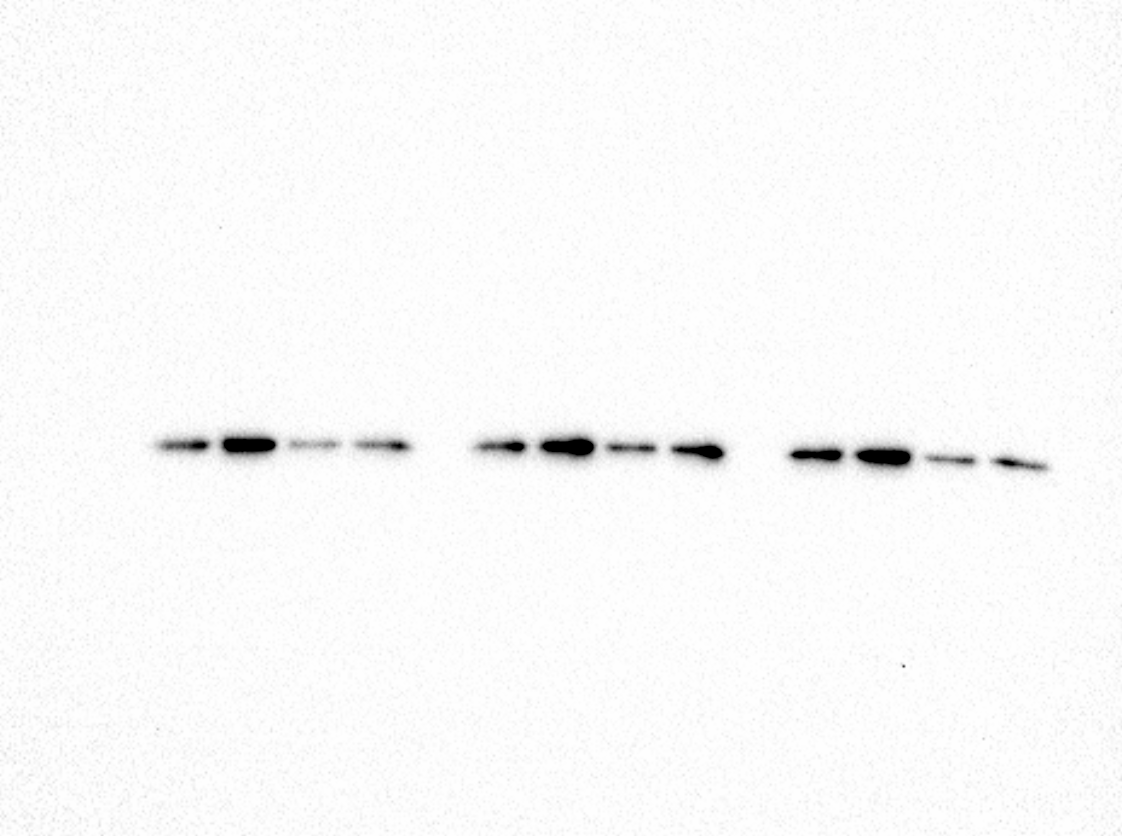

Supplement: Supplementary file 1 — Full and uncropped western blots [file 41419_2025_7809_MOESM1_ESM.zip › Full and uncropped western blots/Fig2C/22RV1/SLC7A11.tif]

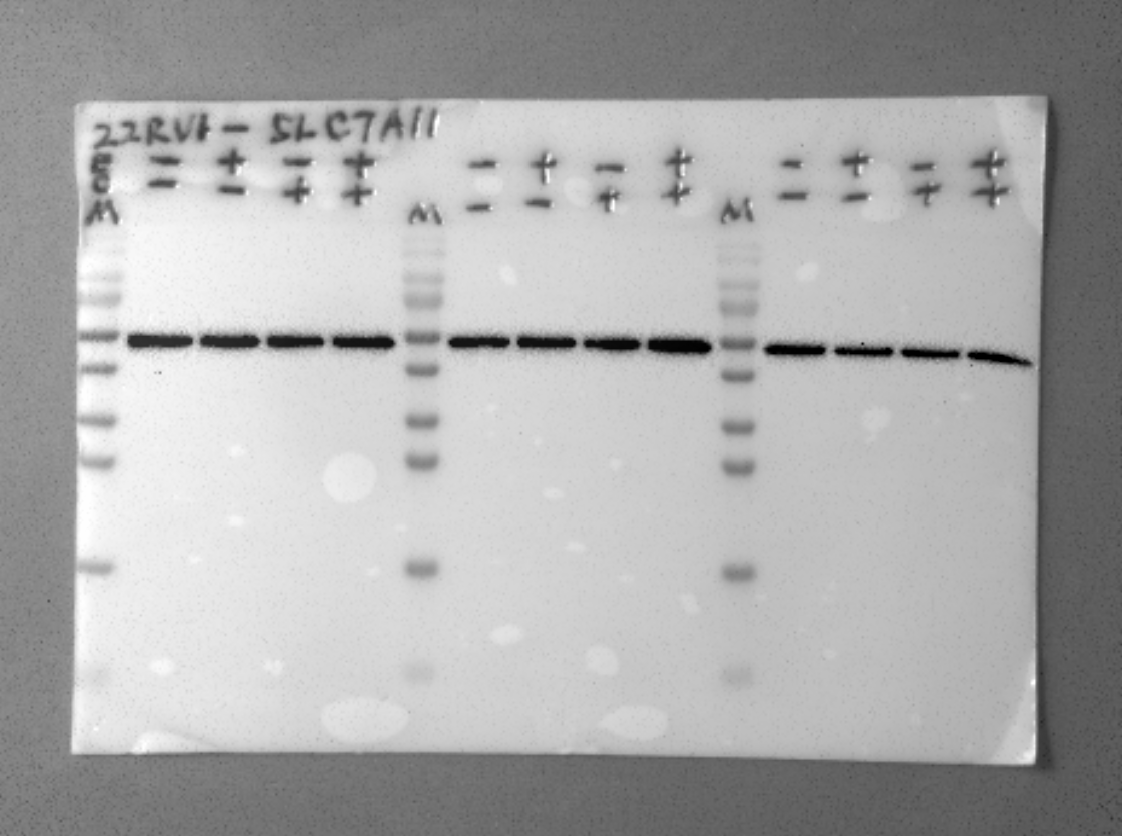

Supplement: Supplementary file 1 — Full and uncropped western blots [file 41419_2025_7809_MOESM1_ESM.zip › Full and uncropped western blots/Fig2C/22RV1/Tubulin-Merge.tif]

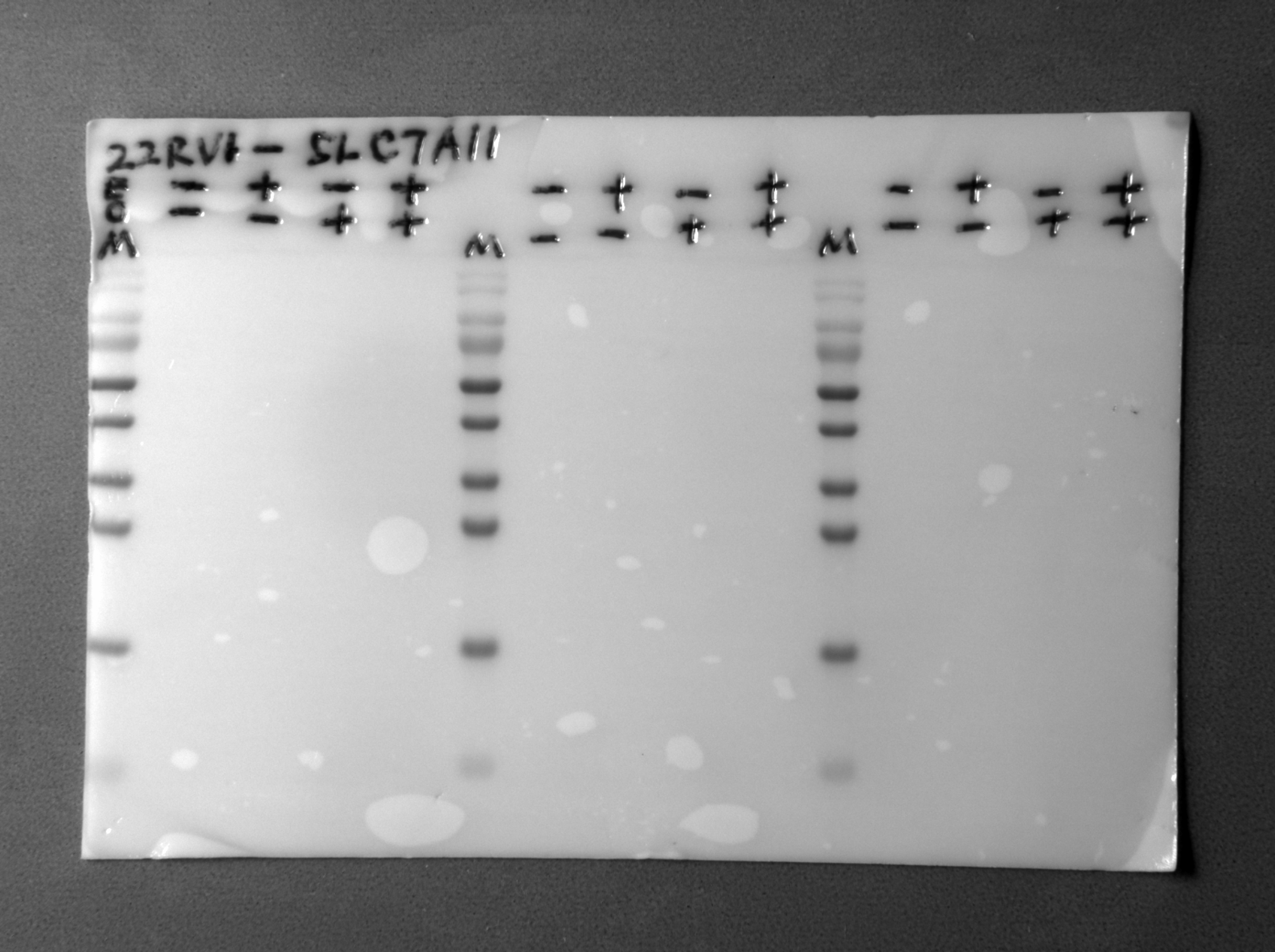

Supplement: Supplementary file 1 — Full and uncropped western blots [file 41419_2025_7809_MOESM1_ESM.zip › Full and uncropped western blots/Fig2C/22RV1/Tubulin-picture of film.tif]

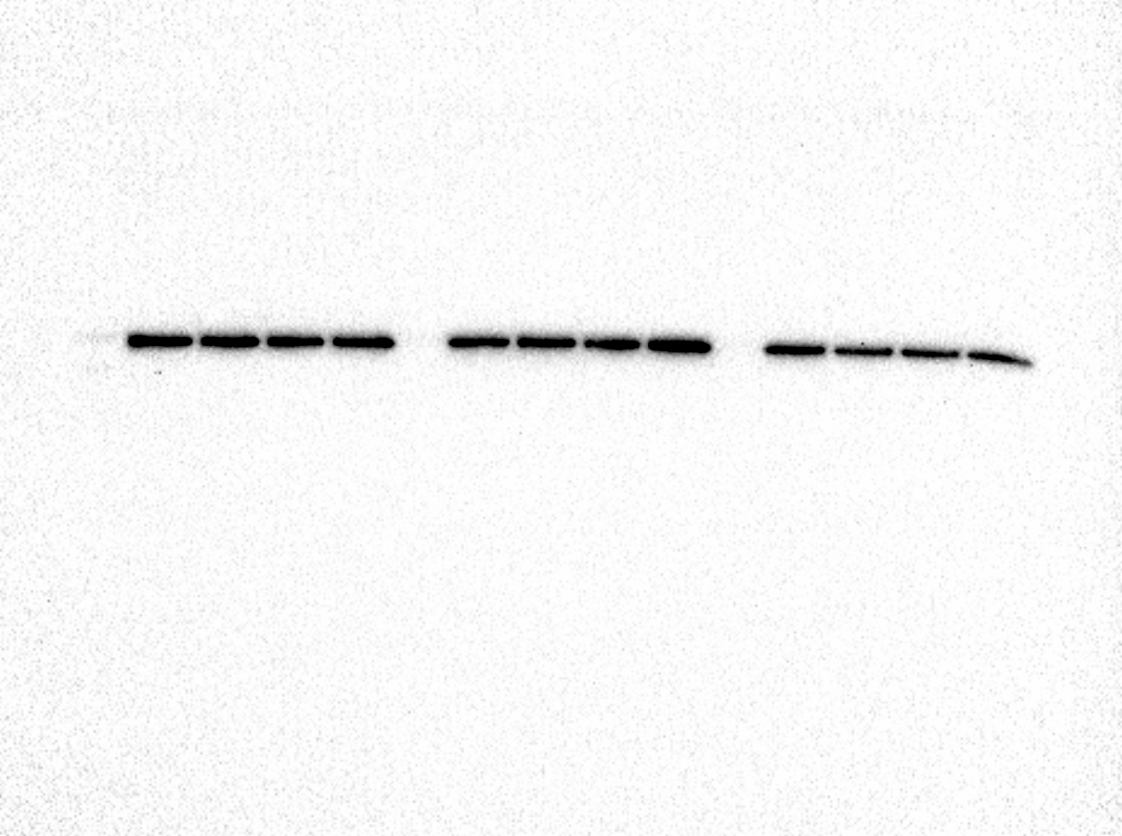

Supplement: Supplementary file 1 — Full and uncropped western blots [file 41419_2025_7809_MOESM1_ESM.zip › Full and uncropped western blots/Fig2C/22RV1/Tubulin.tif]

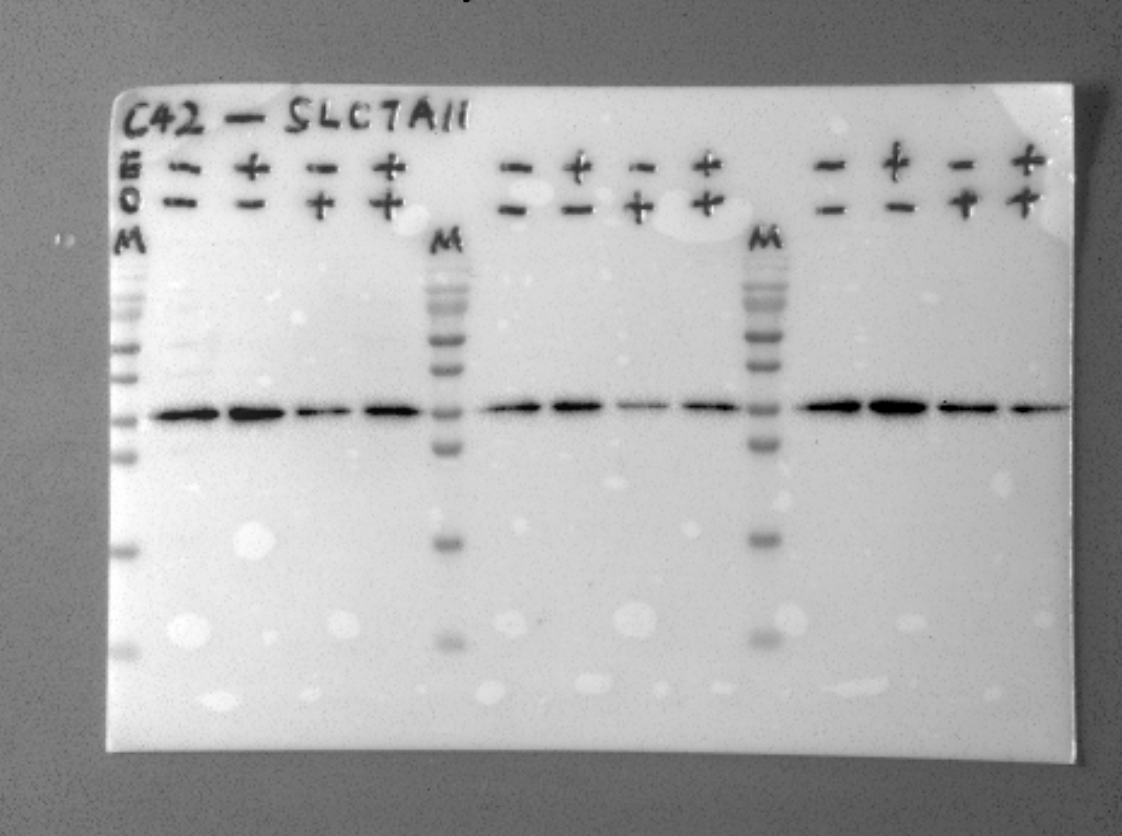

Supplement: Supplementary file 1 — Full and uncropped western blots [file 41419_2025_7809_MOESM1_ESM.zip › Full and uncropped western blots/Fig2C/C4-2/SLC7A11-Merge.tif]

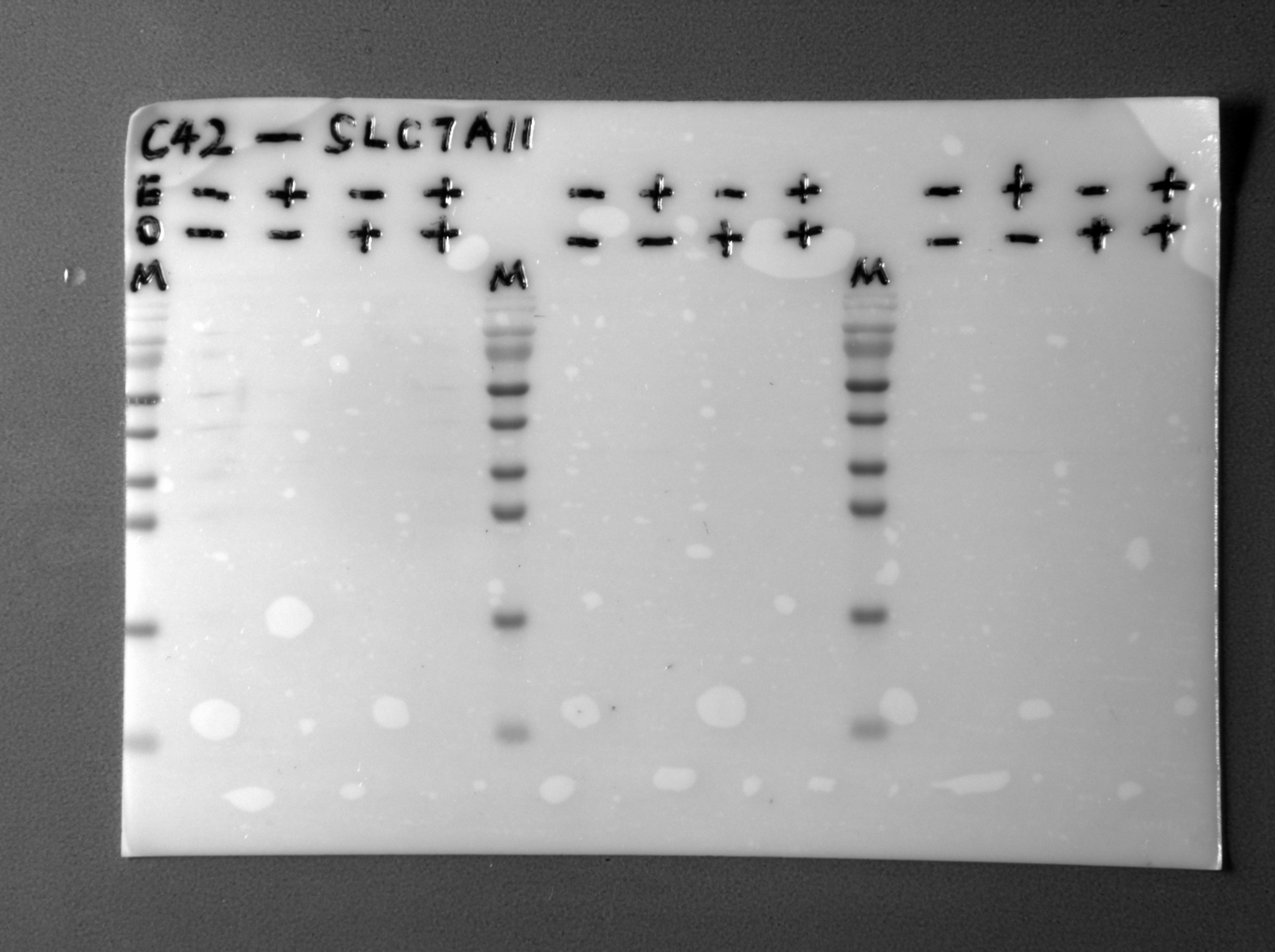

Supplement: Supplementary file 1 — Full and uncropped western blots [file 41419_2025_7809_MOESM1_ESM.zip › Full and uncropped western blots/Fig2C/C4-2/SLC7A11-picture of film.tif]

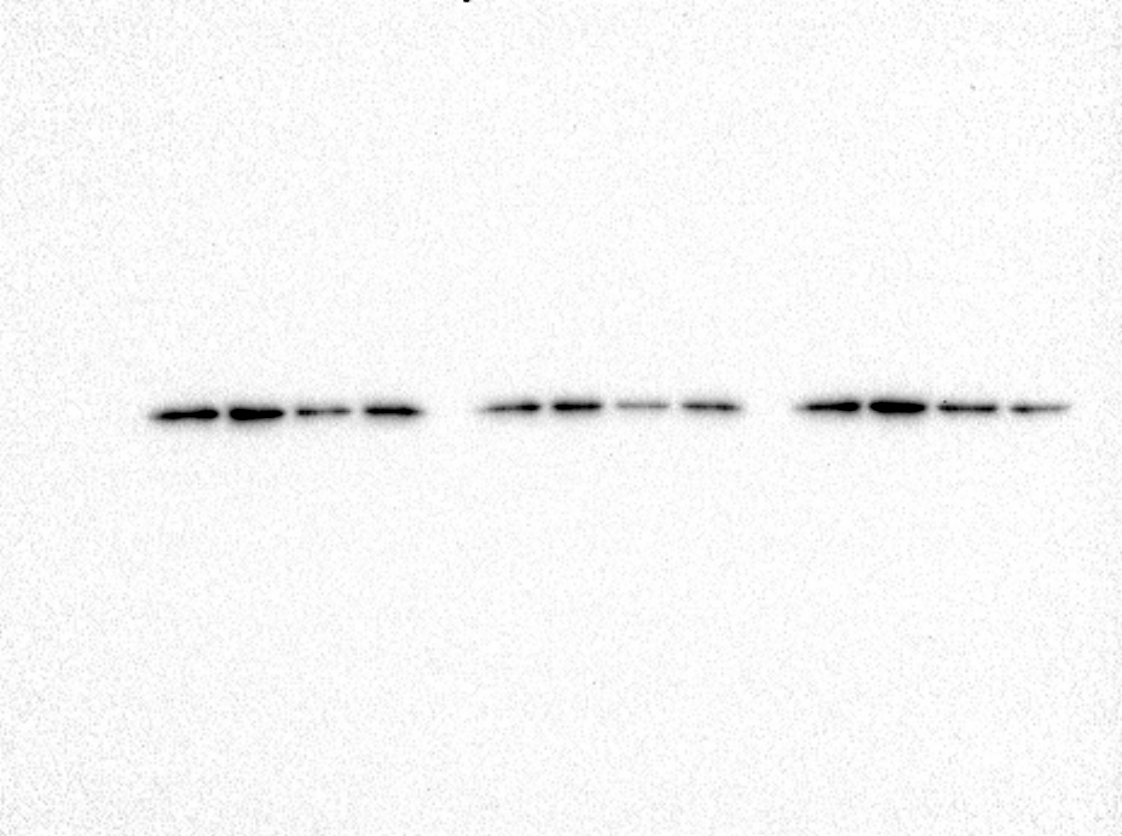

Supplement: Supplementary file 1 — Full and uncropped western blots [file 41419_2025_7809_MOESM1_ESM.zip › Full and uncropped western blots/Fig2C/C4-2/SLC7A11.tif]

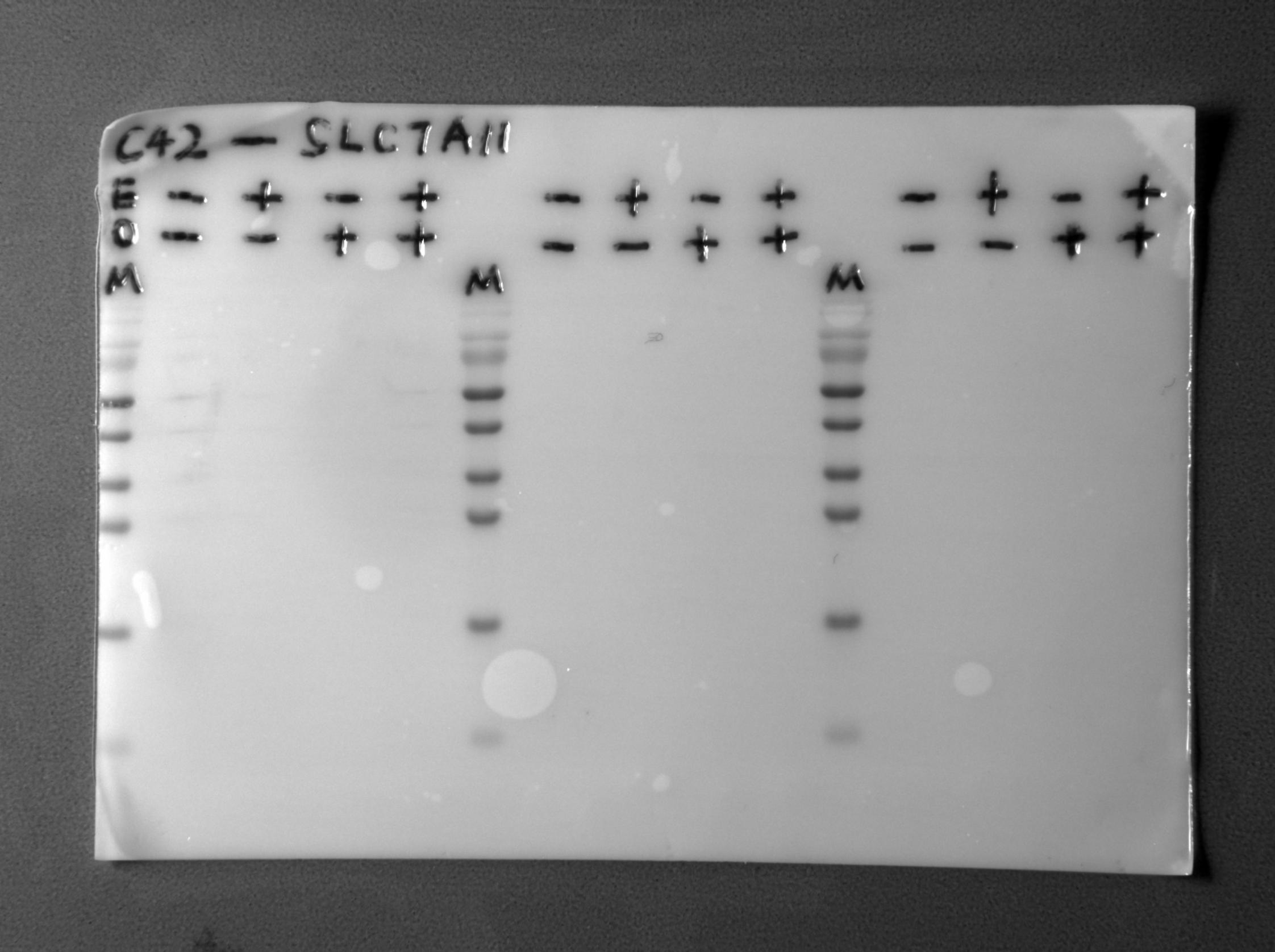

Supplement: Supplementary file 1 — Full and uncropped western blots [file 41419_2025_7809_MOESM1_ESM.zip › Full and uncropped western blots/Fig2C/C4-2/Tubulin-Merge.tif]

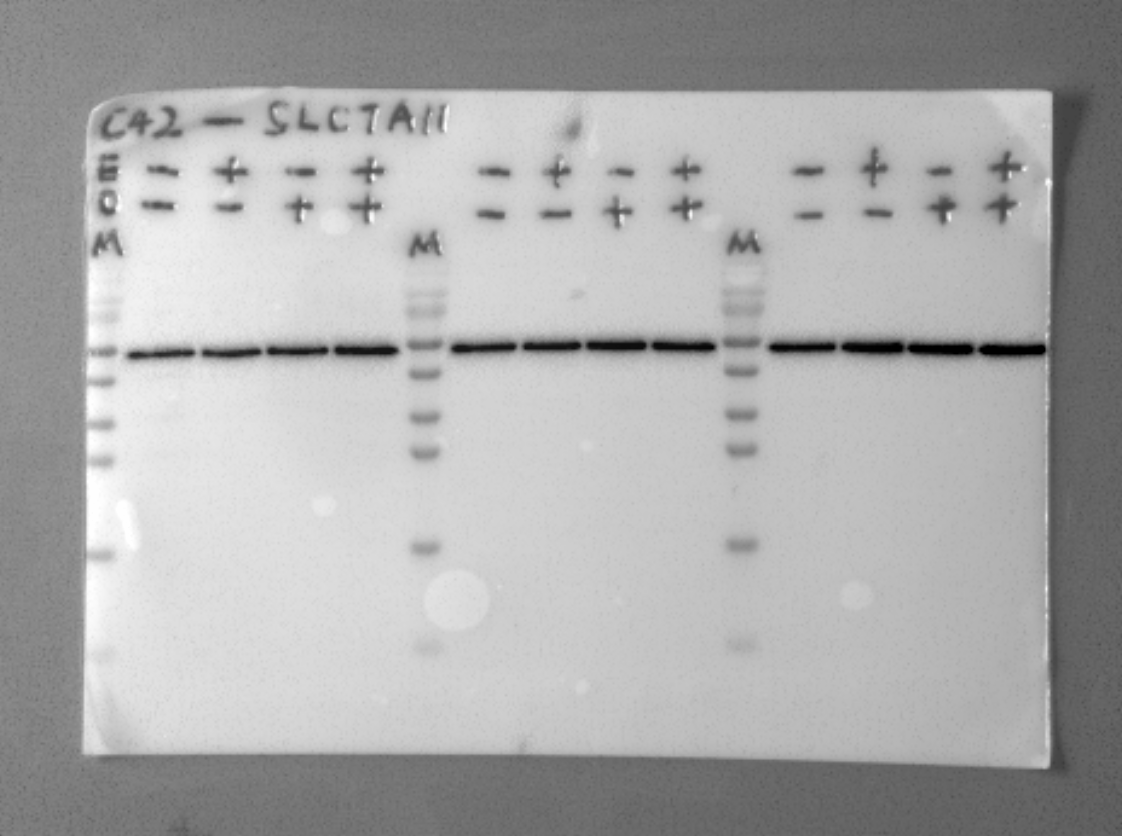

Supplement: Supplementary file 1 — Full and uncropped western blots [file 41419_2025_7809_MOESM1_ESM.zip › Full and uncropped western blots/Fig2C/C4-2/Tubulin-picture of film.tif]

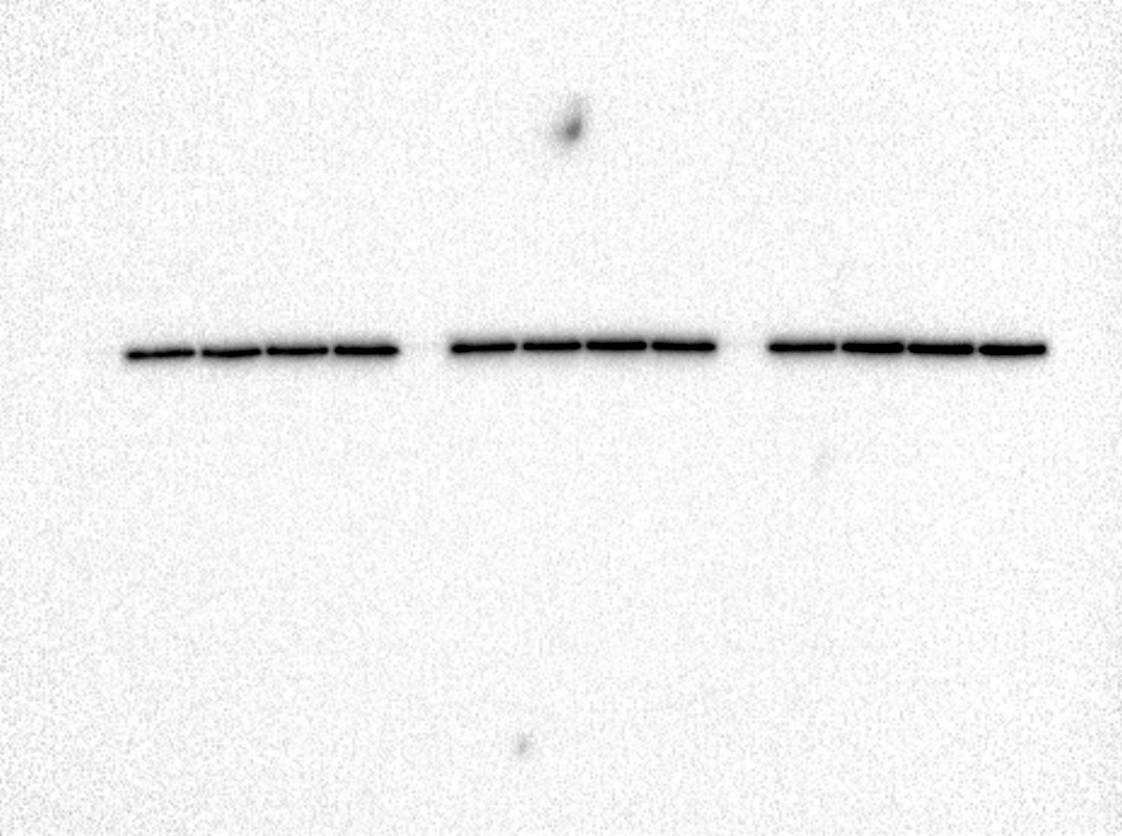

Supplement: Supplementary file 1 — Full and uncropped western blots [file 41419_2025_7809_MOESM1_ESM.zip › Full and uncropped western blots/Fig2C/C4-2/Tubulin.tif]

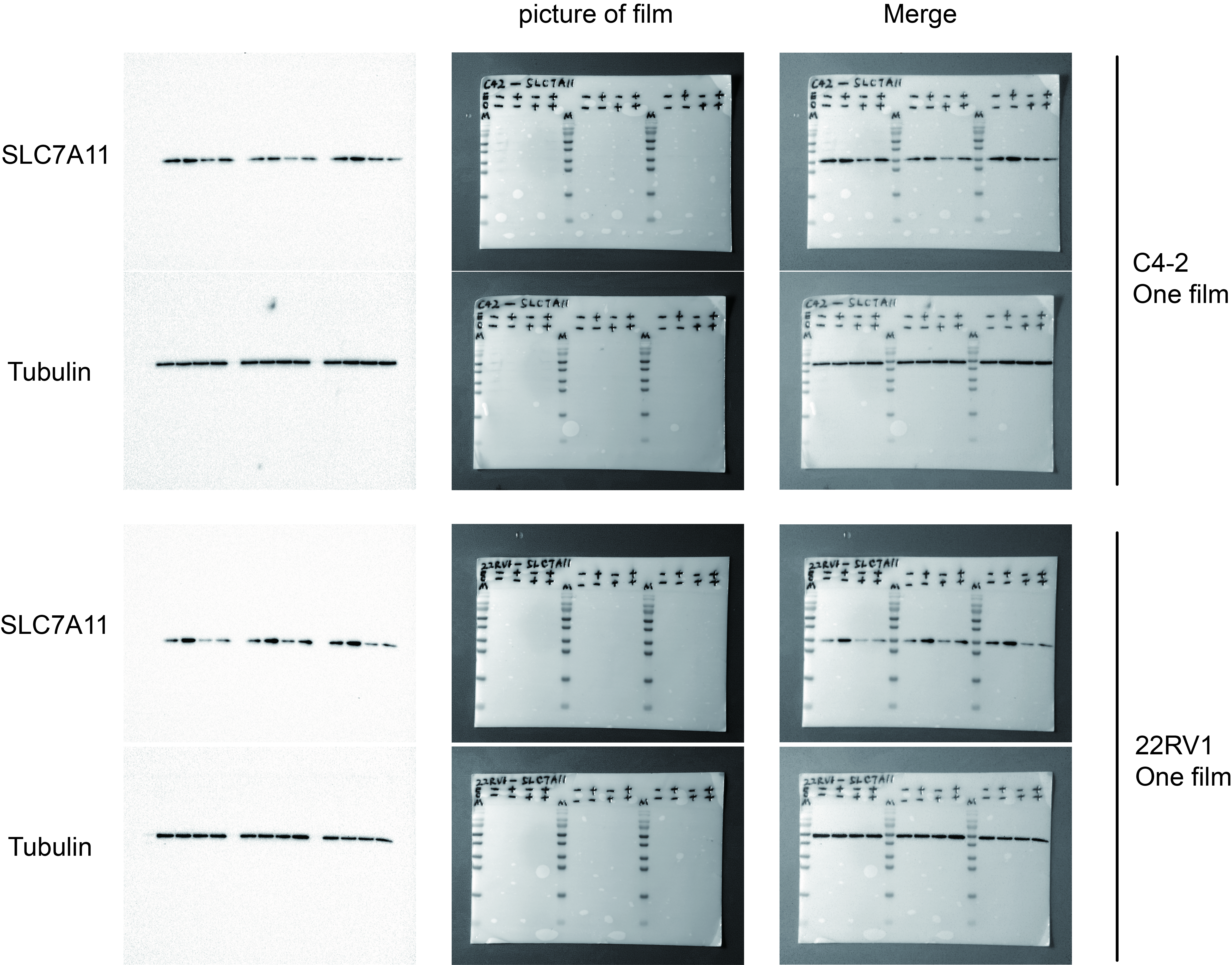

Supplement: Supplementary file 1 — Full and uncropped western blots [file 41419_2025_7809_MOESM1_ESM.zip › Full and uncropped western blots/Fig2C.tif]

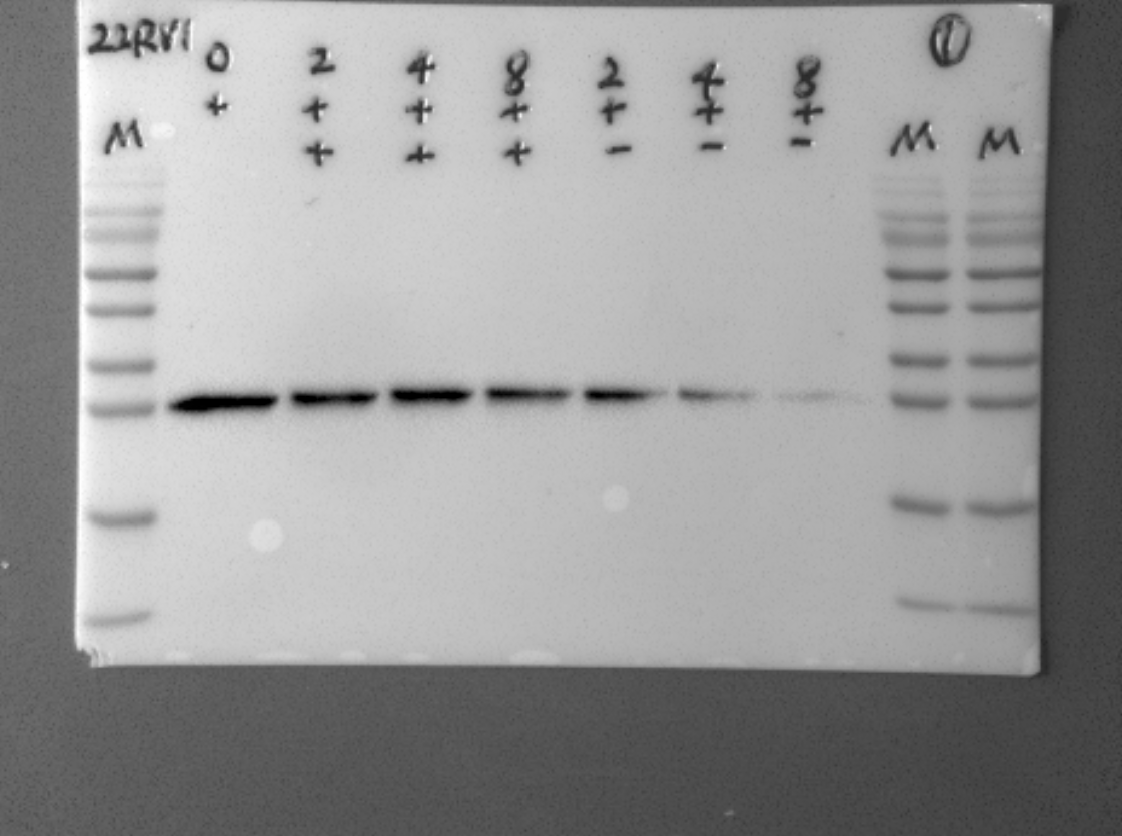

Supplement: Supplementary file 1 — Full and uncropped western blots [file 41419_2025_7809_MOESM1_ESM.zip › Full and uncropped western blots/Fig5C/22RV1/film1/SLC7A11-Merge.tif]

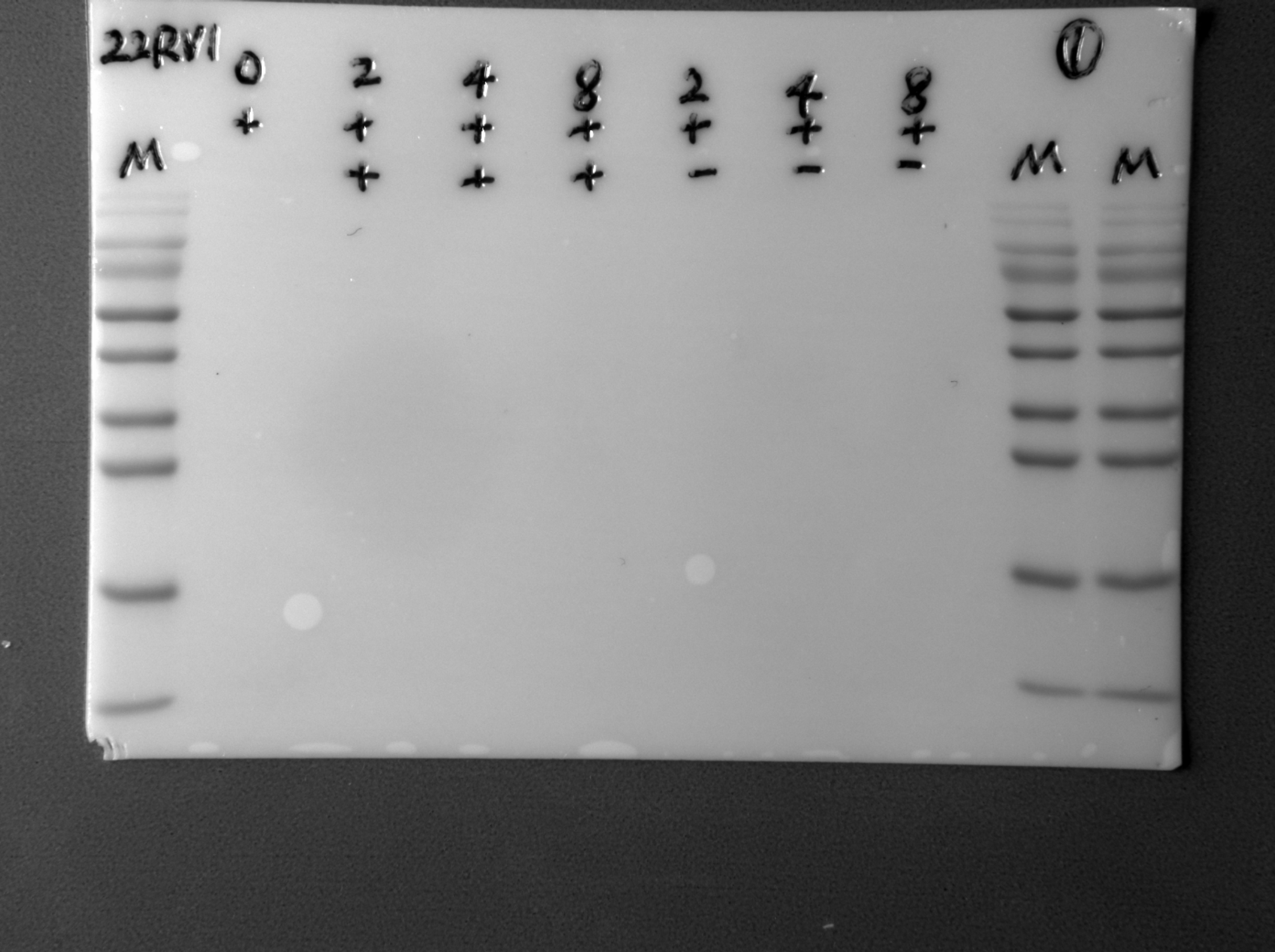

Supplement: Supplementary file 1 — Full and uncropped western blots [file 41419_2025_7809_MOESM1_ESM.zip › Full and uncropped western blots/Fig5C/22RV1/film1/SLC7A11-picture of film.tif]

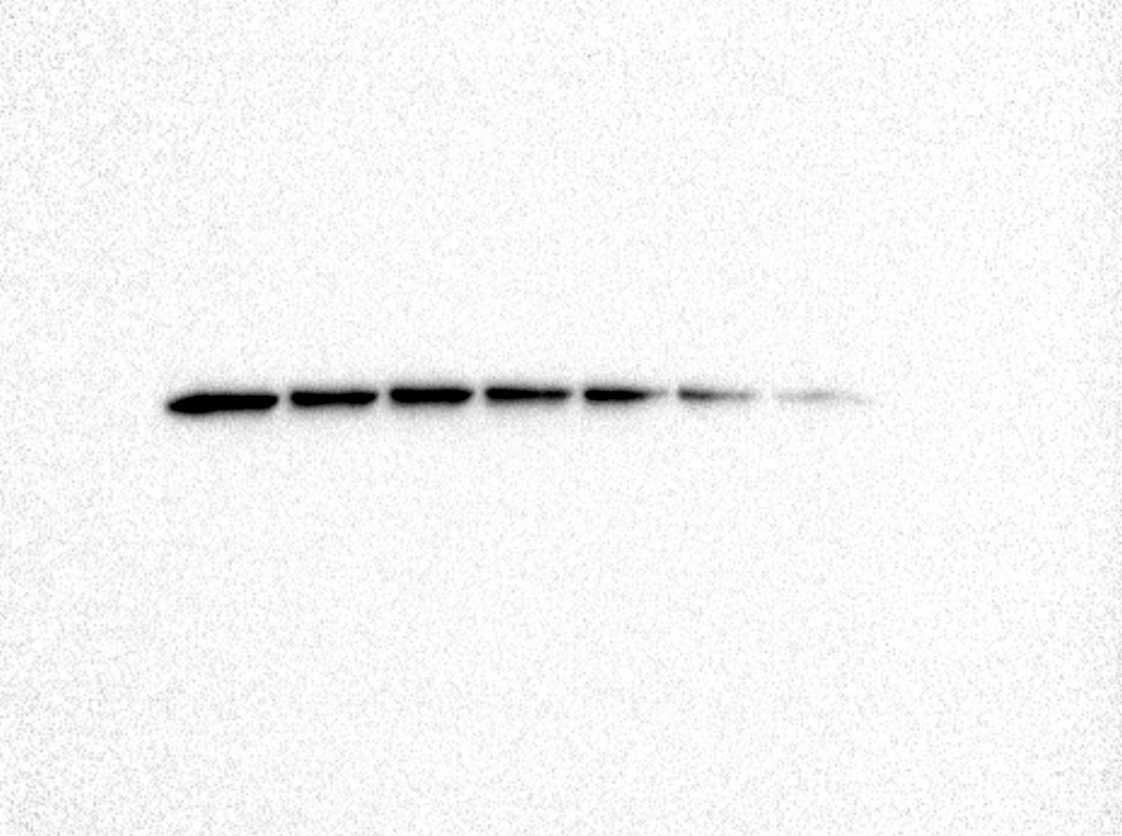

Supplement: Supplementary file 1 — Full and uncropped western blots [file 41419_2025_7809_MOESM1_ESM.zip › Full and uncropped western blots/Fig5C/22RV1/film1/SLC7A11.tif]

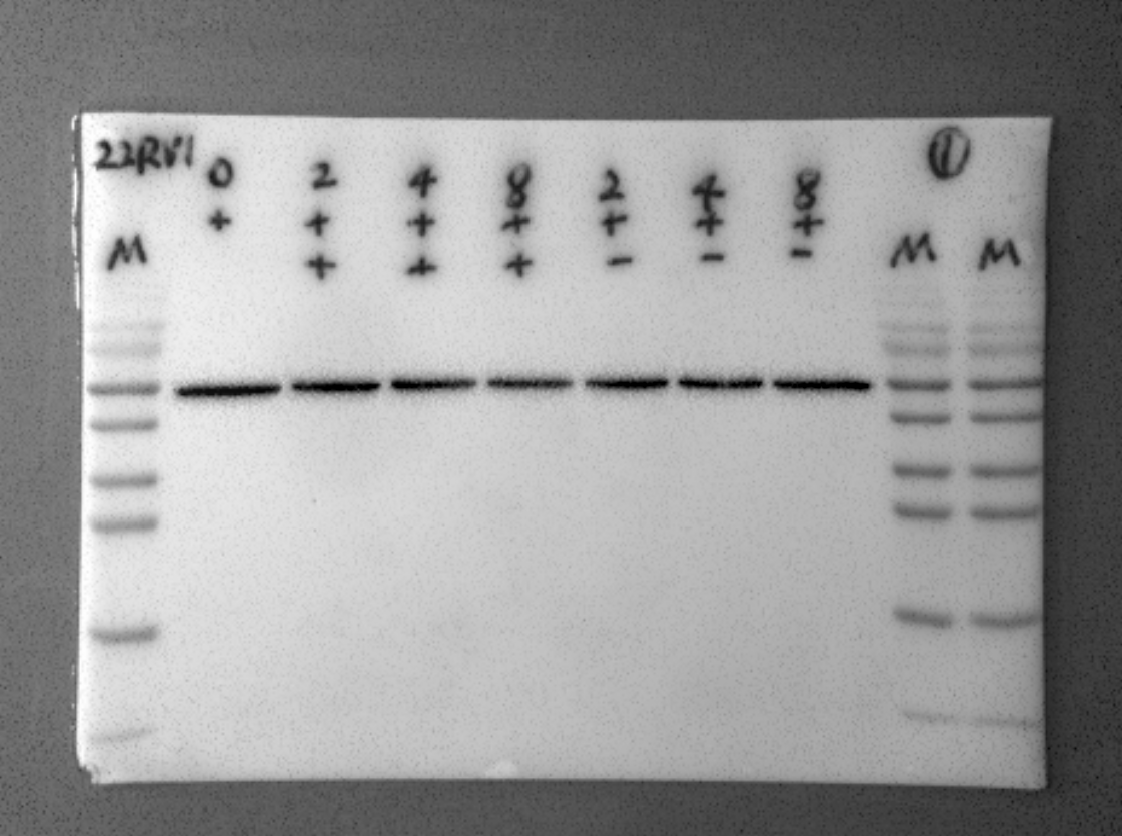

Supplement: Supplementary file 1 — Full and uncropped western blots [file 41419_2025_7809_MOESM1_ESM.zip › Full and uncropped western blots/Fig5C/22RV1/film1/Tubulin-Merge.tif]

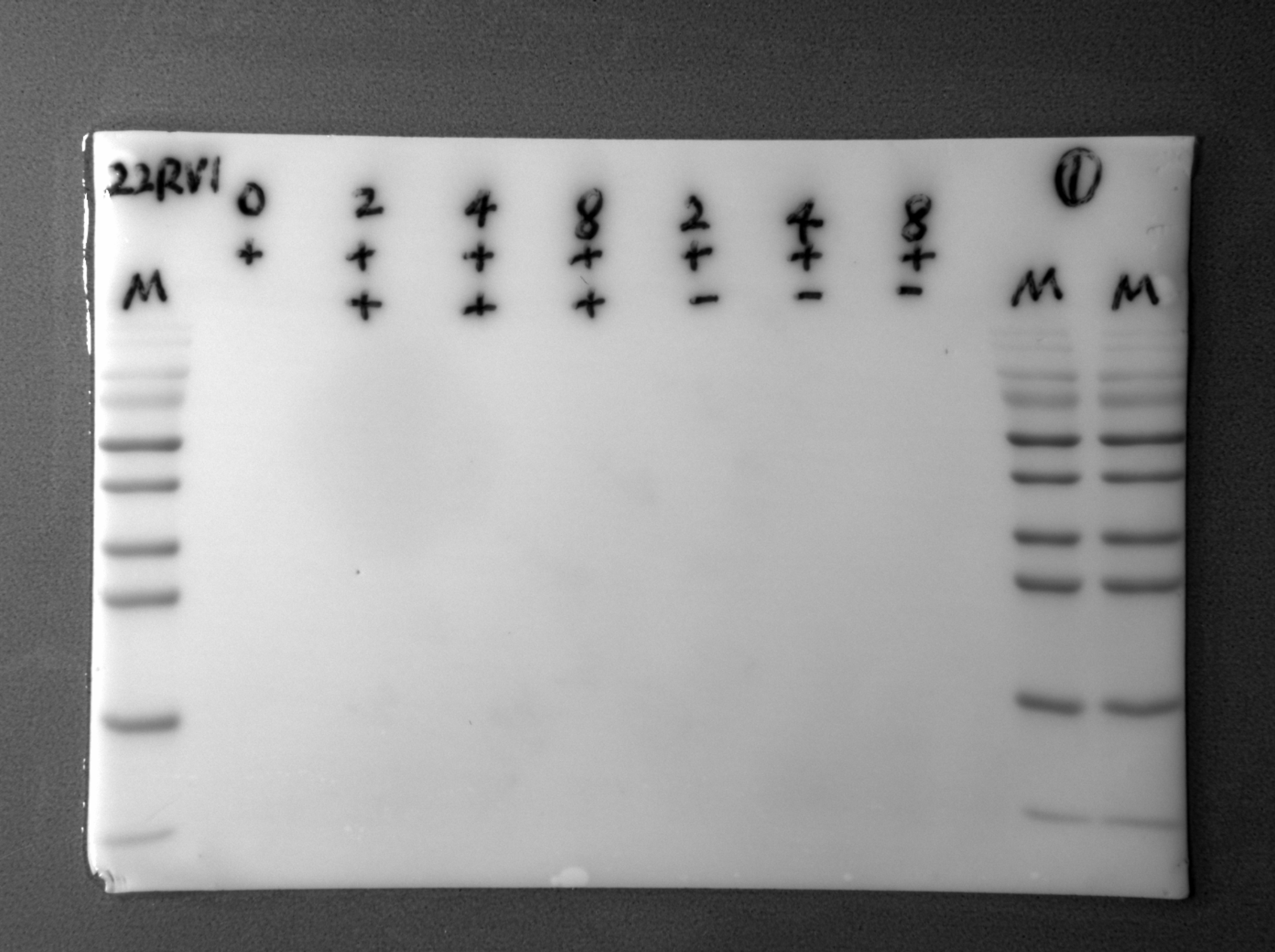

Supplement: Supplementary file 1 — Full and uncropped western blots [file 41419_2025_7809_MOESM1_ESM.zip › Full and uncropped western blots/Fig5C/22RV1/film1/Tubulin-picture of film.tif]

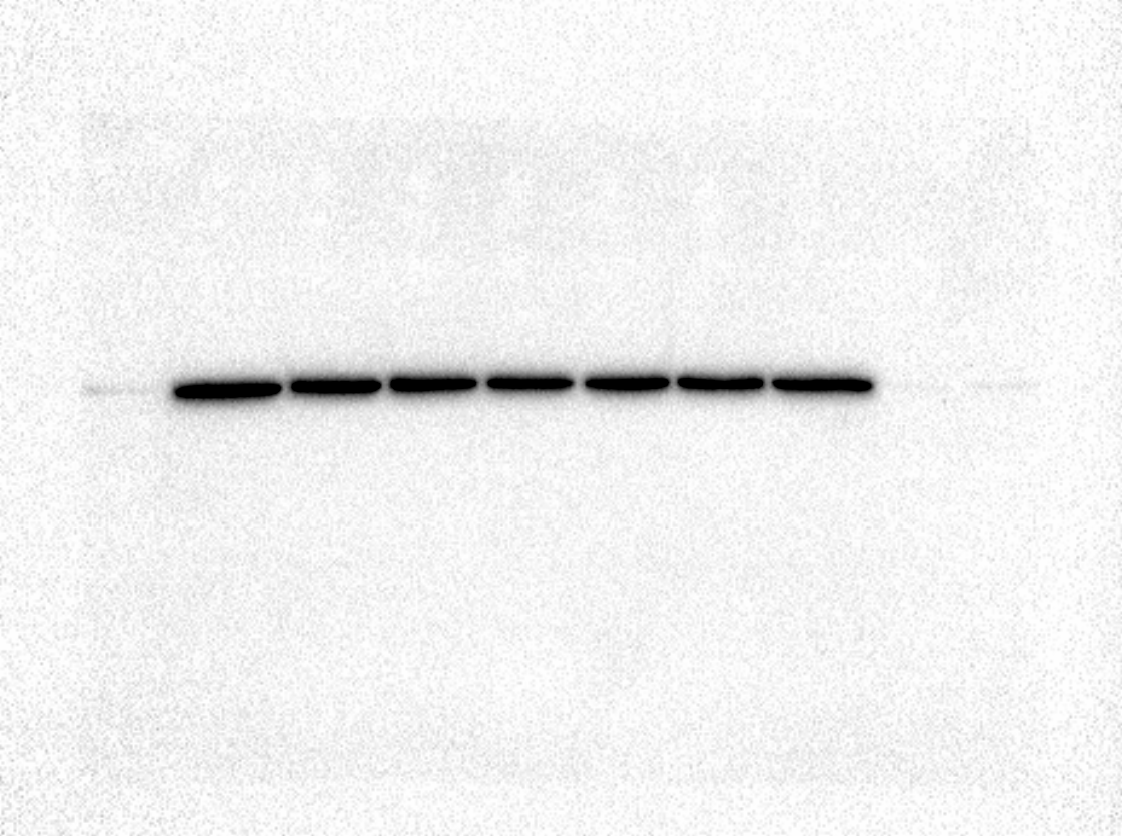

Supplement: Supplementary file 1 — Full and uncropped western blots [file 41419_2025_7809_MOESM1_ESM.zip › Full and uncropped western blots/Fig5C/22RV1/film1/Tubulin.tif]

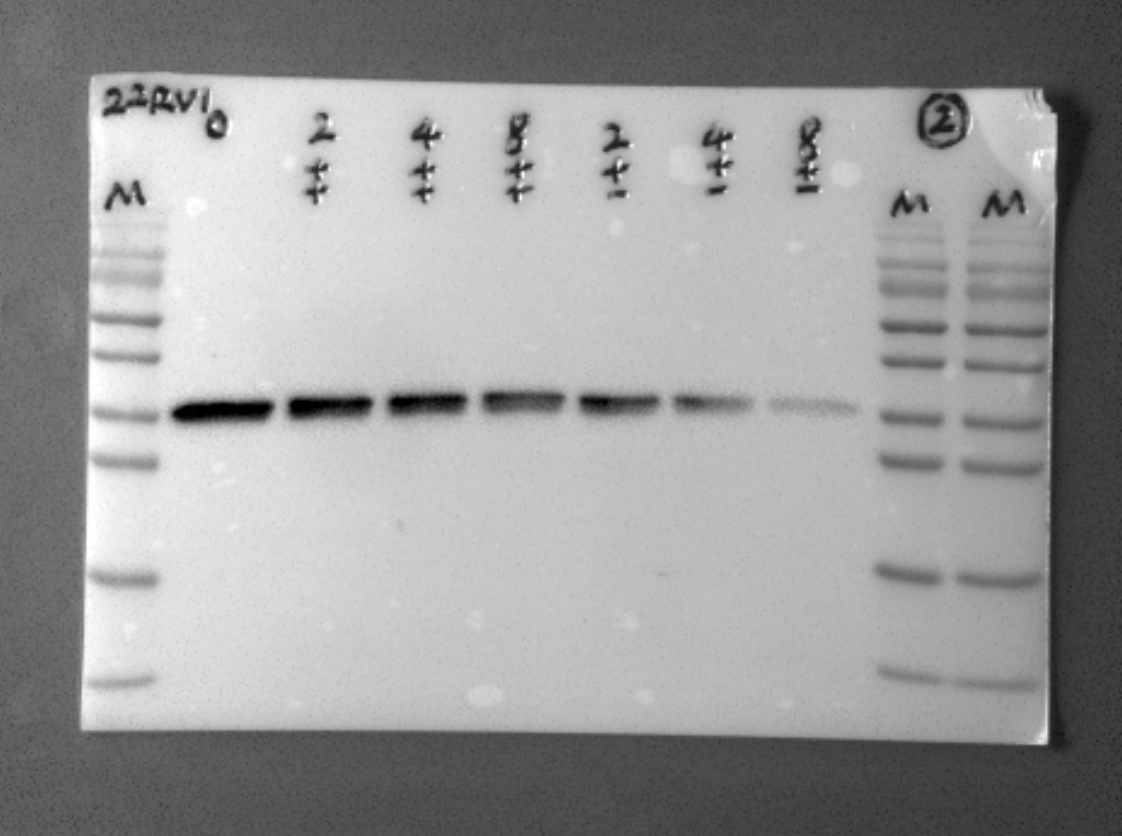

Supplement: Supplementary file 1 — Full and uncropped western blots [file 41419_2025_7809_MOESM1_ESM.zip › Full and uncropped western blots/Fig5C/22RV1/film2/SLC7A11-Merge.tif]

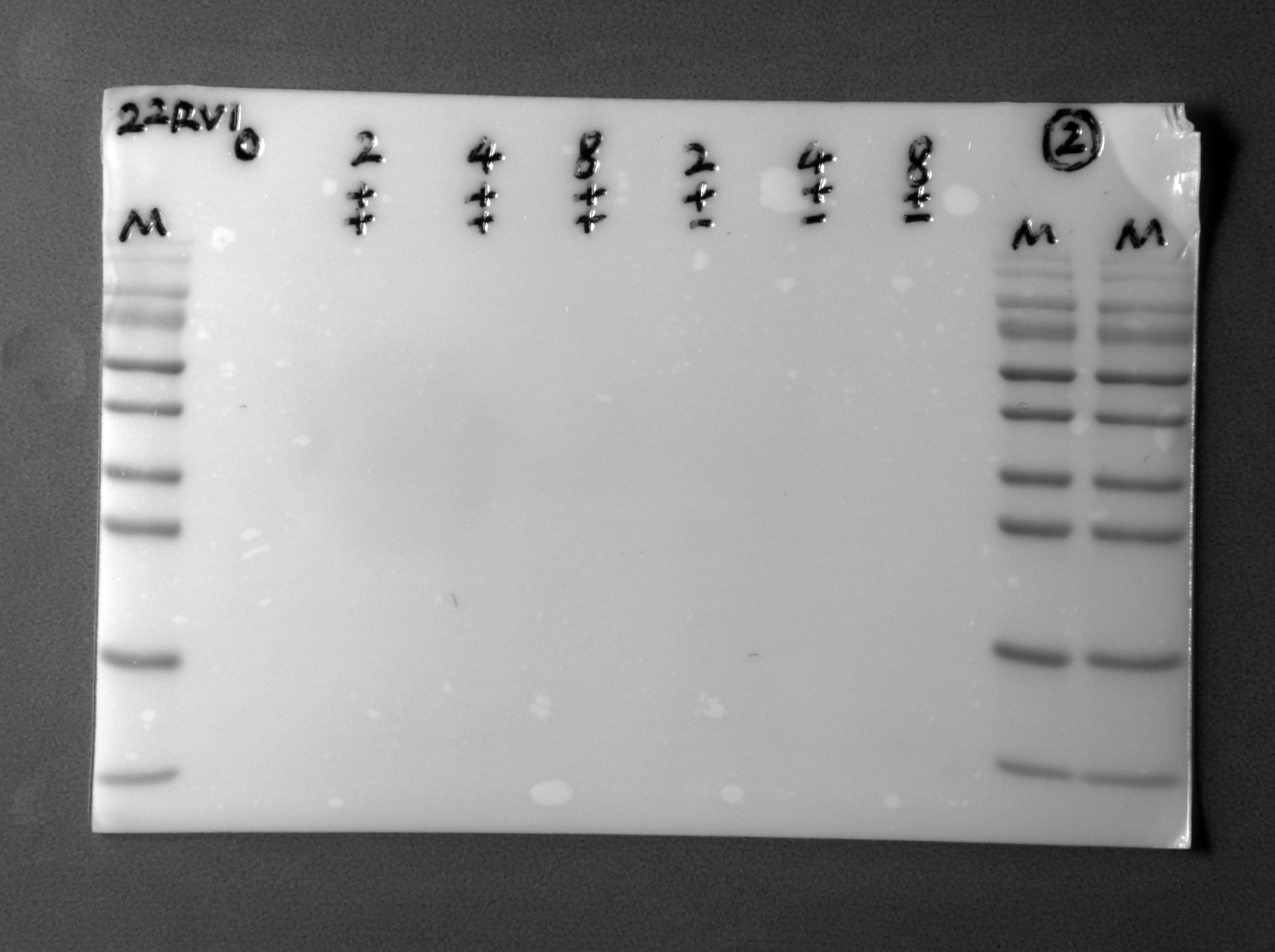

Supplement: Supplementary file 1 — Full and uncropped western blots [file 41419_2025_7809_MOESM1_ESM.zip › Full and uncropped western blots/Fig5C/22RV1/film2/SLC7A11-picture of film.tif]

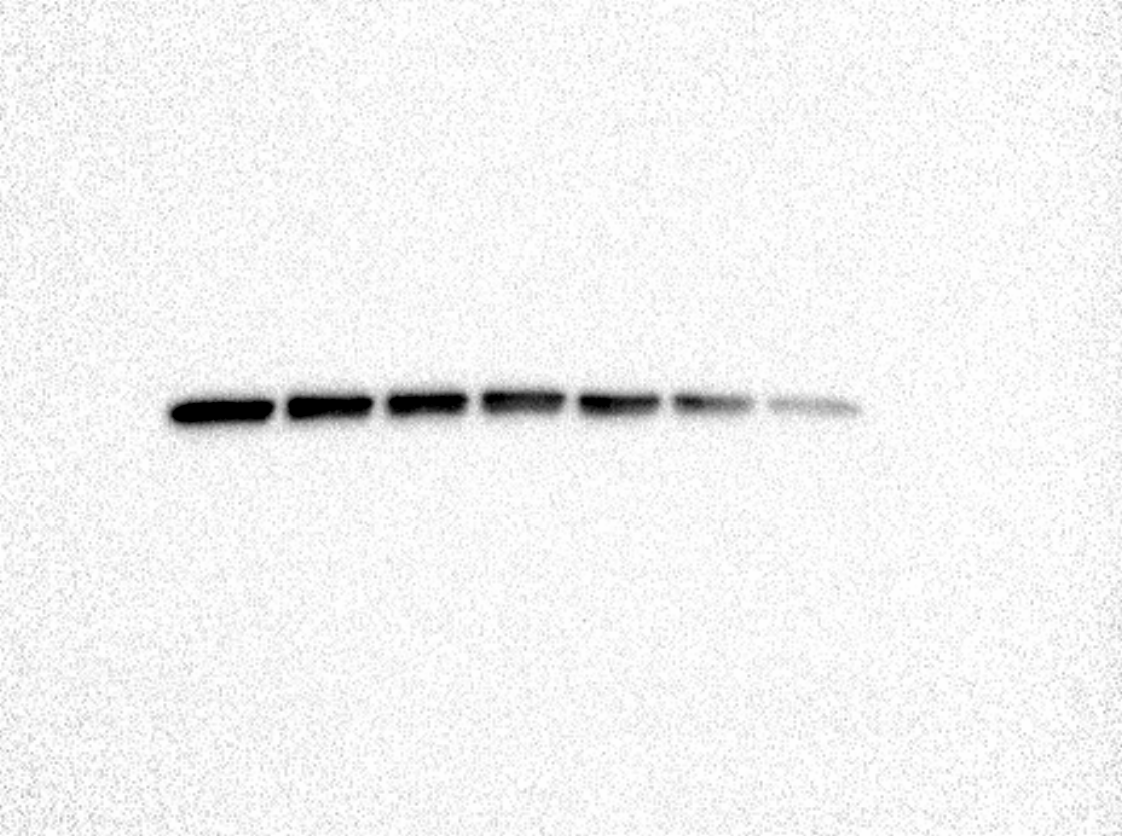

Supplement: Supplementary file 1 — Full and uncropped western blots [file 41419_2025_7809_MOESM1_ESM.zip › Full and uncropped western blots/Fig5C/22RV1/film2/SLC7A11.tif]

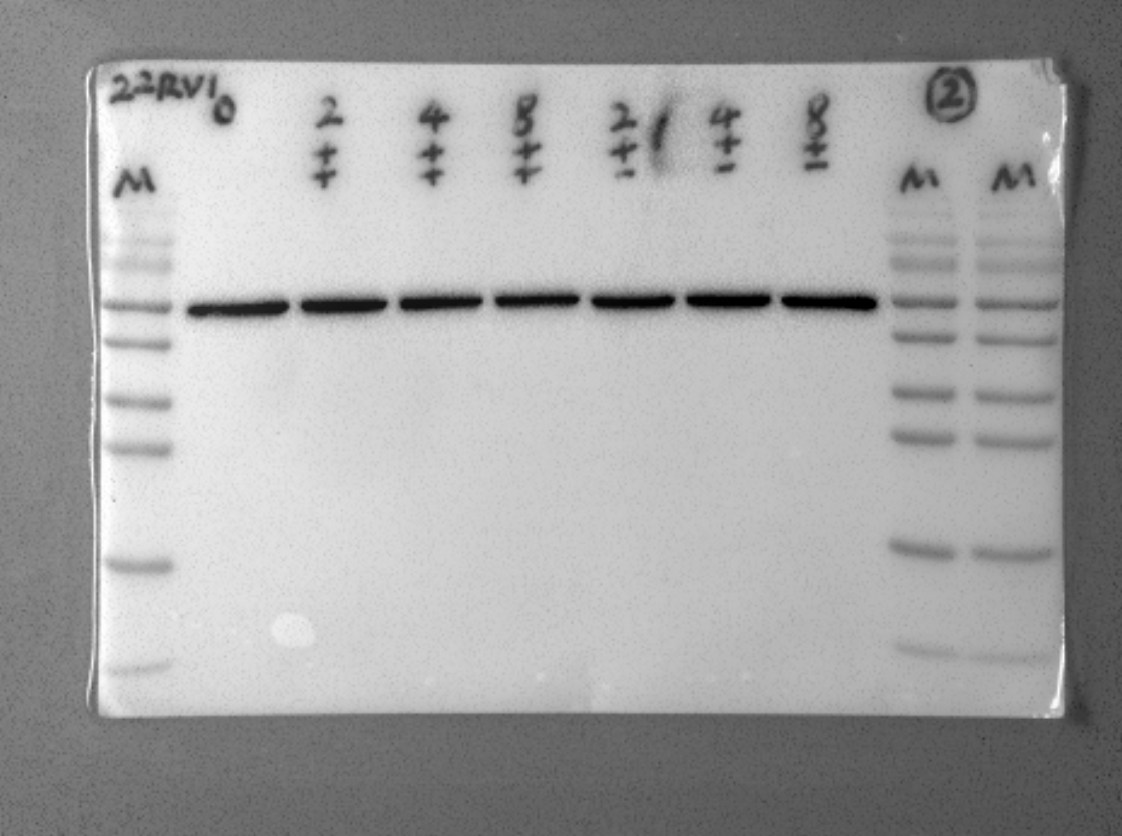

Supplement: Supplementary file 1 — Full and uncropped western blots [file 41419_2025_7809_MOESM1_ESM.zip › Full and uncropped western blots/Fig5C/22RV1/film2/Tubulin-Merge.tif]

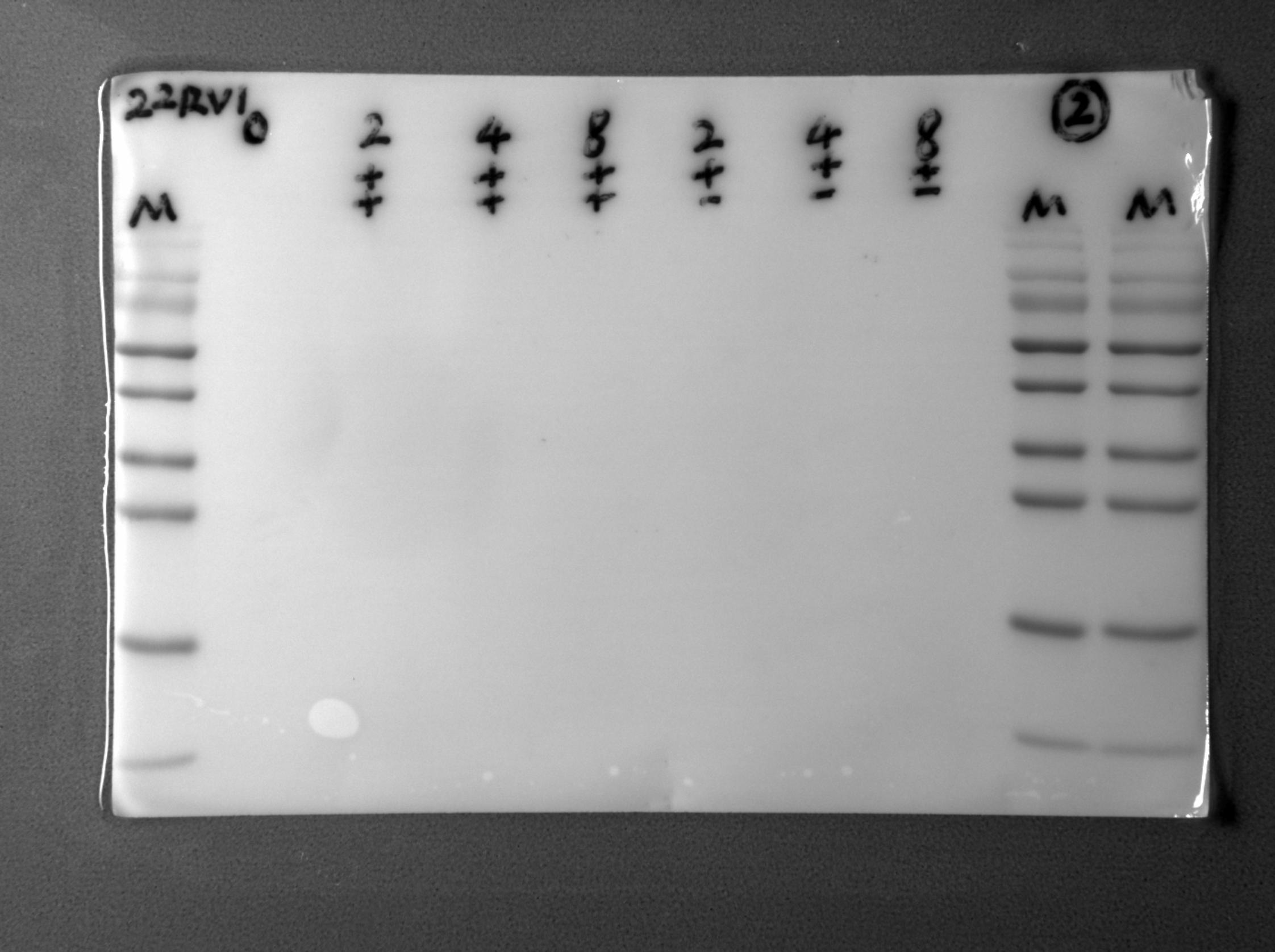

Supplement: Supplementary file 1 — Full and uncropped western blots [file 41419_2025_7809_MOESM1_ESM.zip › Full and uncropped western blots/Fig5C/22RV1/film2/Tubulin-picture of film.tif]

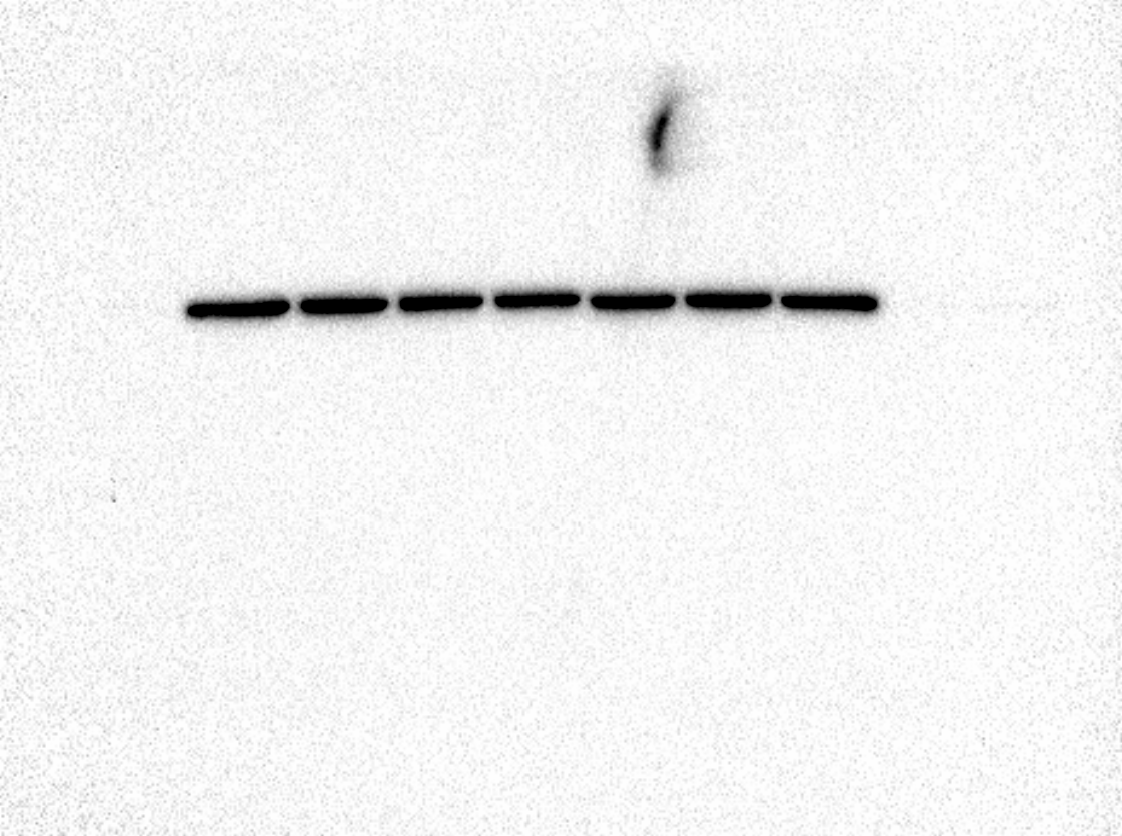

Supplement: Supplementary file 1 — Full and uncropped western blots [file 41419_2025_7809_MOESM1_ESM.zip › Full and uncropped western blots/Fig5C/22RV1/film2/Tubulin.tif]

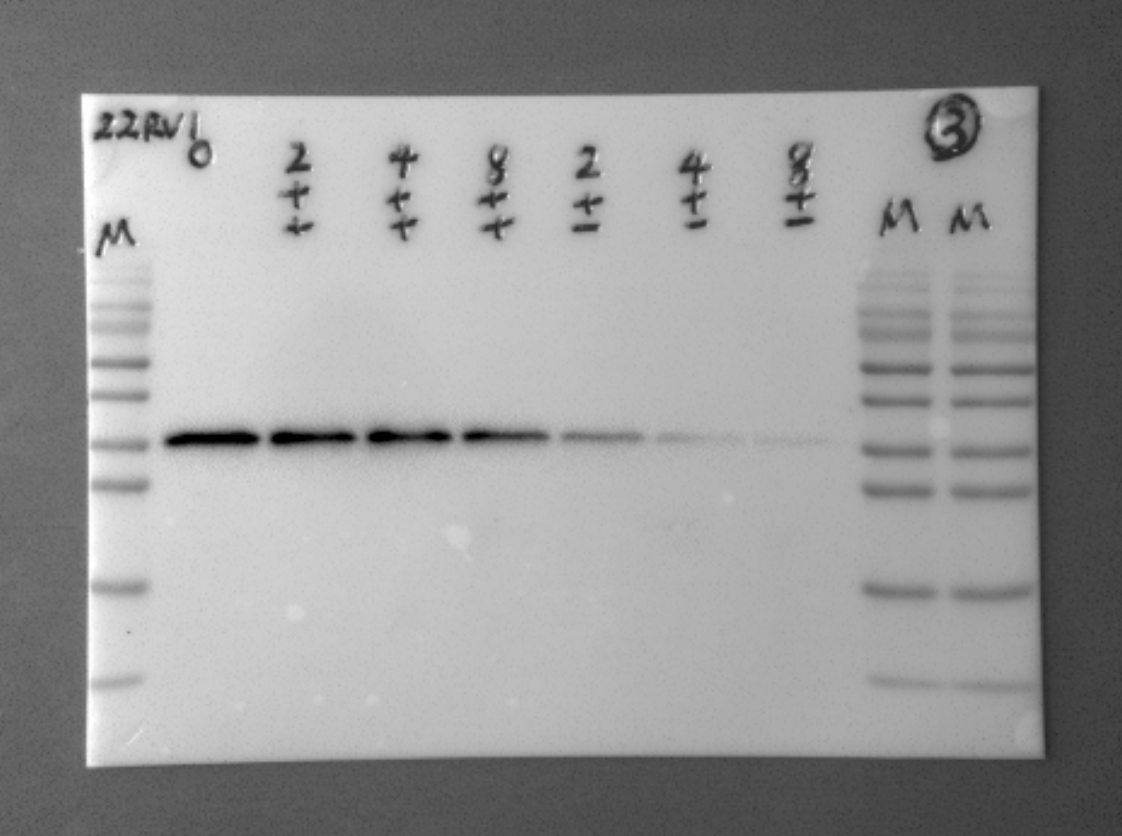

Supplement: Supplementary file 1 — Full and uncropped western blots [file 41419_2025_7809_MOESM1_ESM.zip › Full and uncropped western blots/Fig5C/22RV1/film3/SLC7A11-Merge.tif]

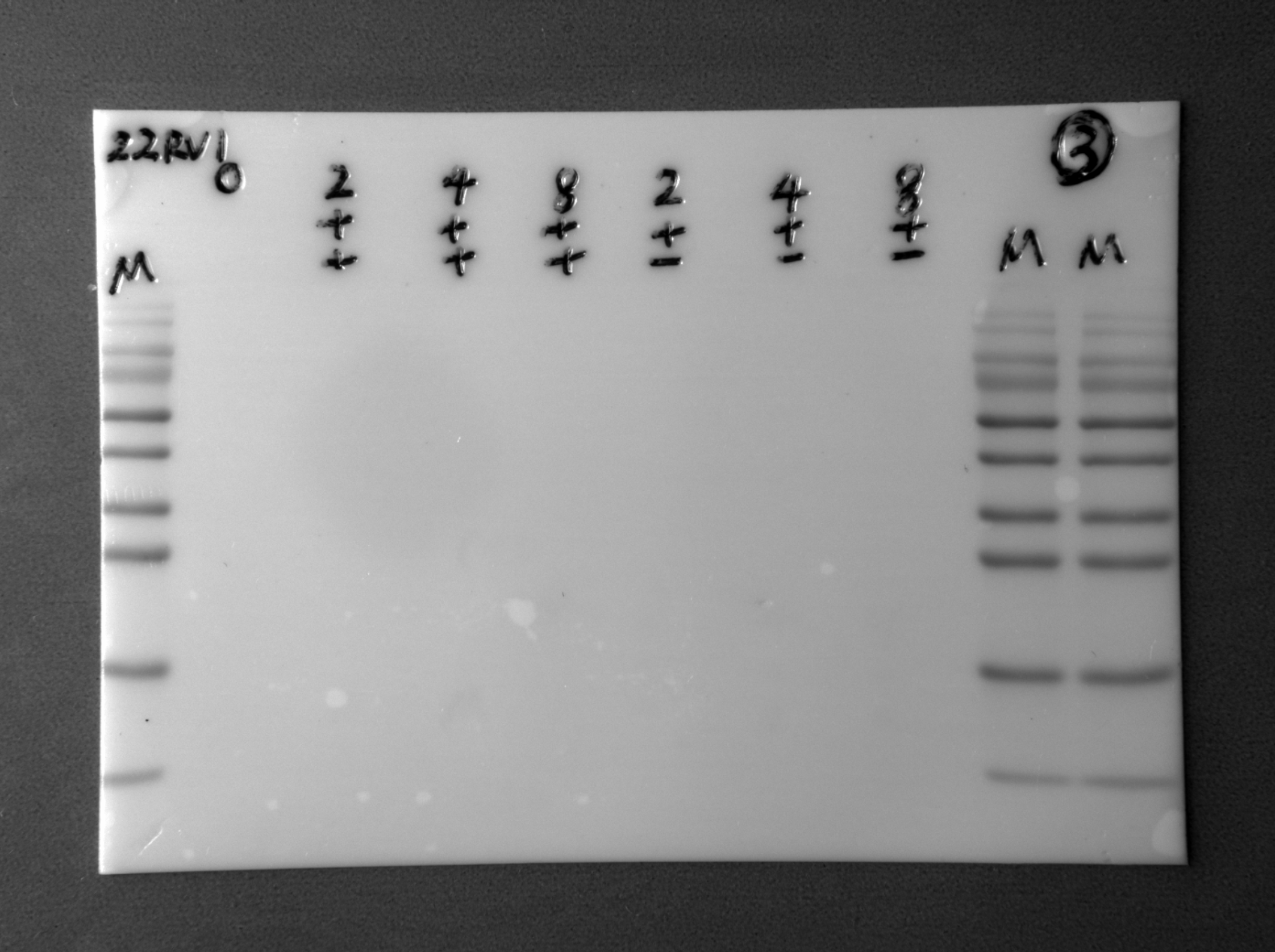

Supplement: Supplementary file 1 — Full and uncropped western blots [file 41419_2025_7809_MOESM1_ESM.zip › Full and uncropped western blots/Fig5C/22RV1/film3/SLC7A11-picture of film.tif]

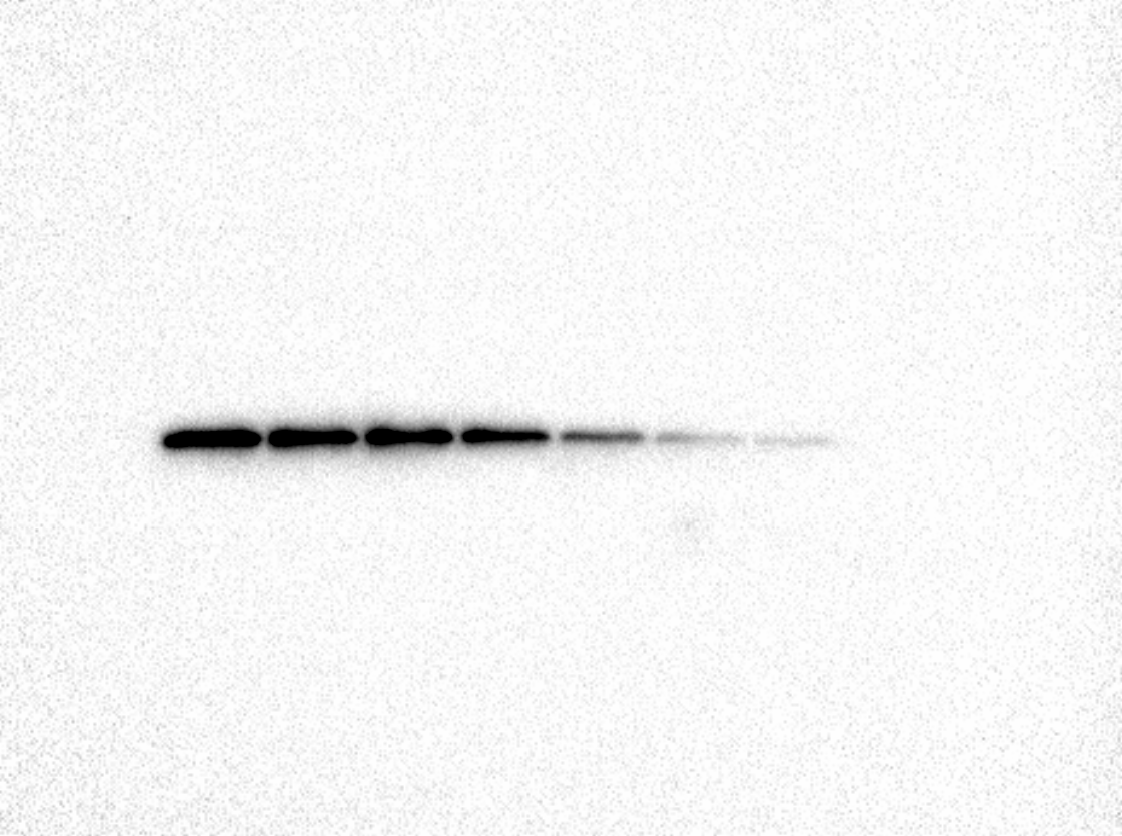

Supplement: Supplementary file 1 — Full and uncropped western blots [file 41419_2025_7809_MOESM1_ESM.zip › Full and uncropped western blots/Fig5C/22RV1/film3/SLC7A11.tif]

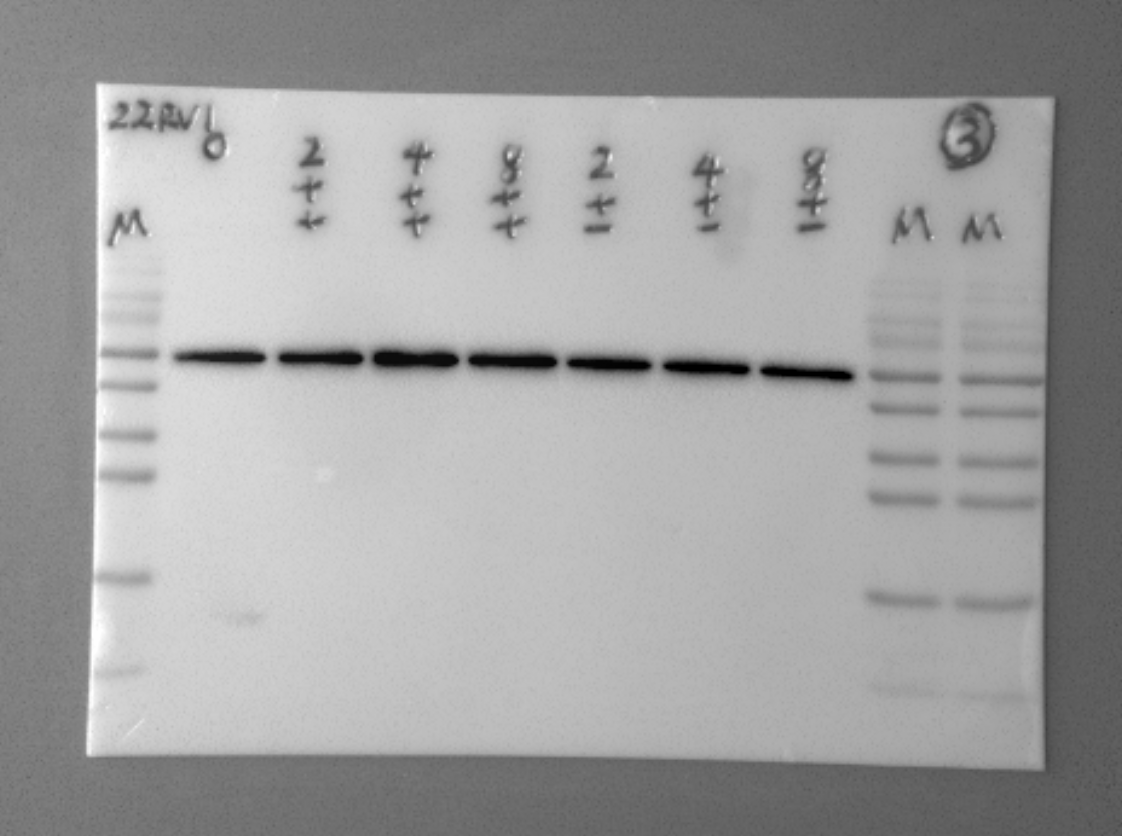

Supplement: Supplementary file 1 — Full and uncropped western blots [file 41419_2025_7809_MOESM1_ESM.zip › Full and uncropped western blots/Fig5C/22RV1/film3/Tubulin-Merge.tif]

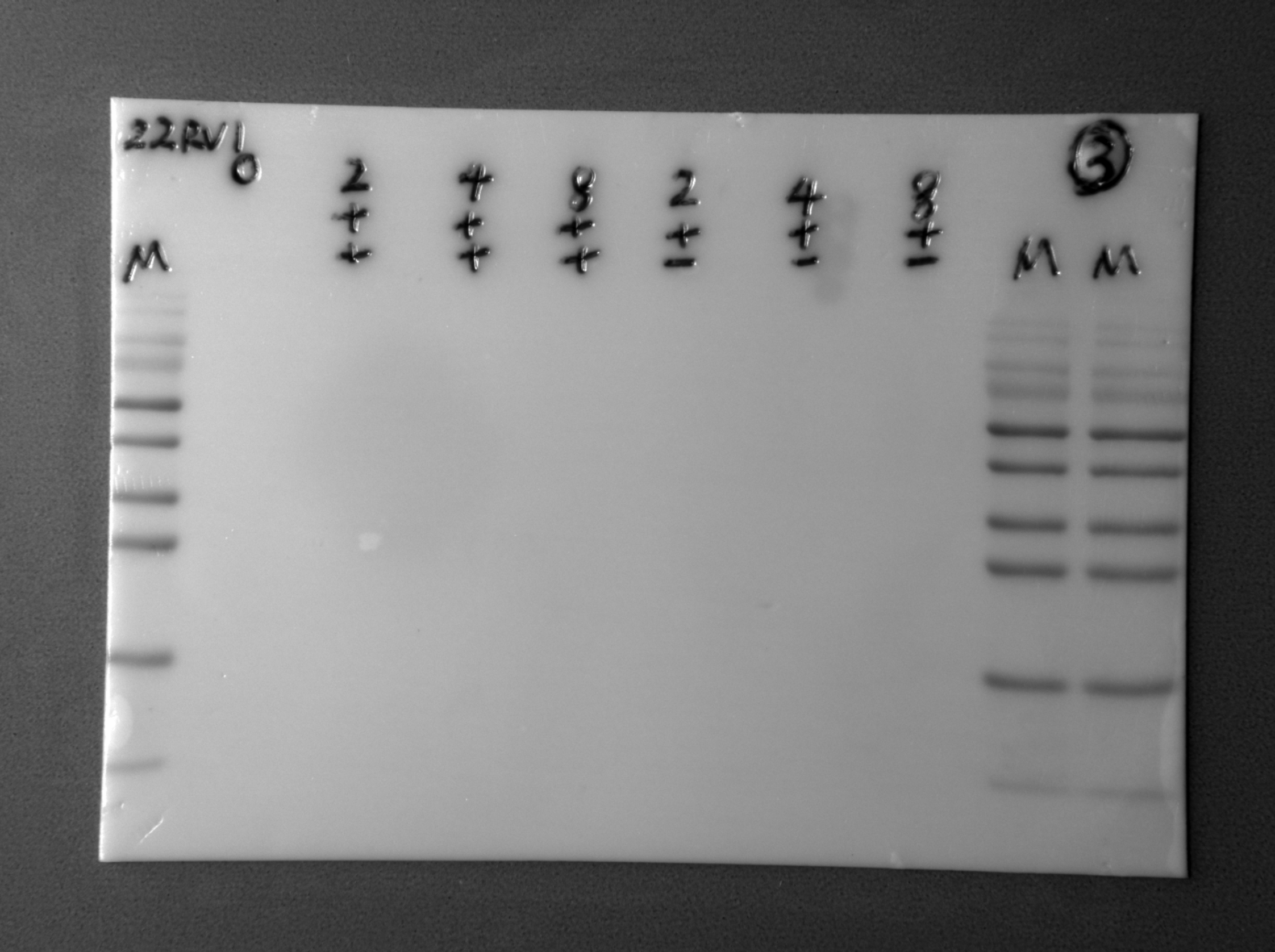

Supplement: Supplementary file 1 — Full and uncropped western blots [file 41419_2025_7809_MOESM1_ESM.zip › Full and uncropped western blots/Fig5C/22RV1/film3/Tubulin-picture of film.tif]

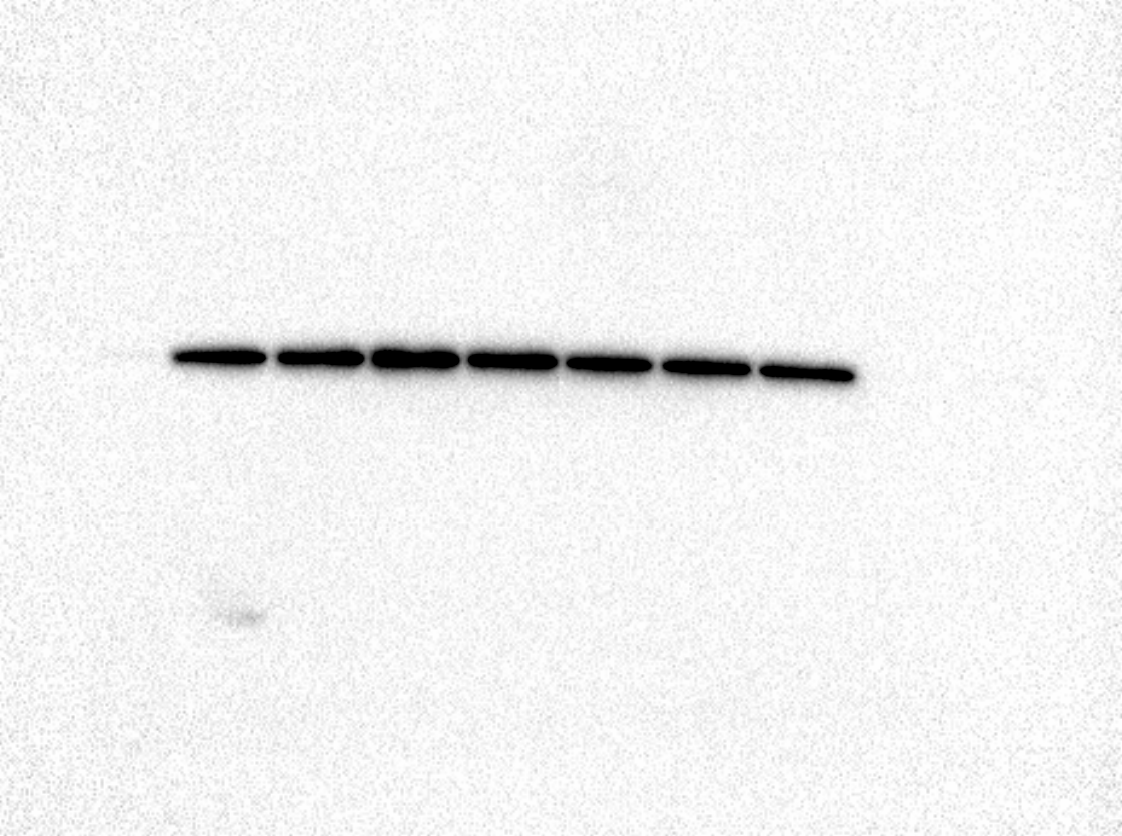

Supplement: Supplementary file 1 — Full and uncropped western blots [file 41419_2025_7809_MOESM1_ESM.zip › Full and uncropped western blots/Fig5C/22RV1/film3/Tubulin.tif]

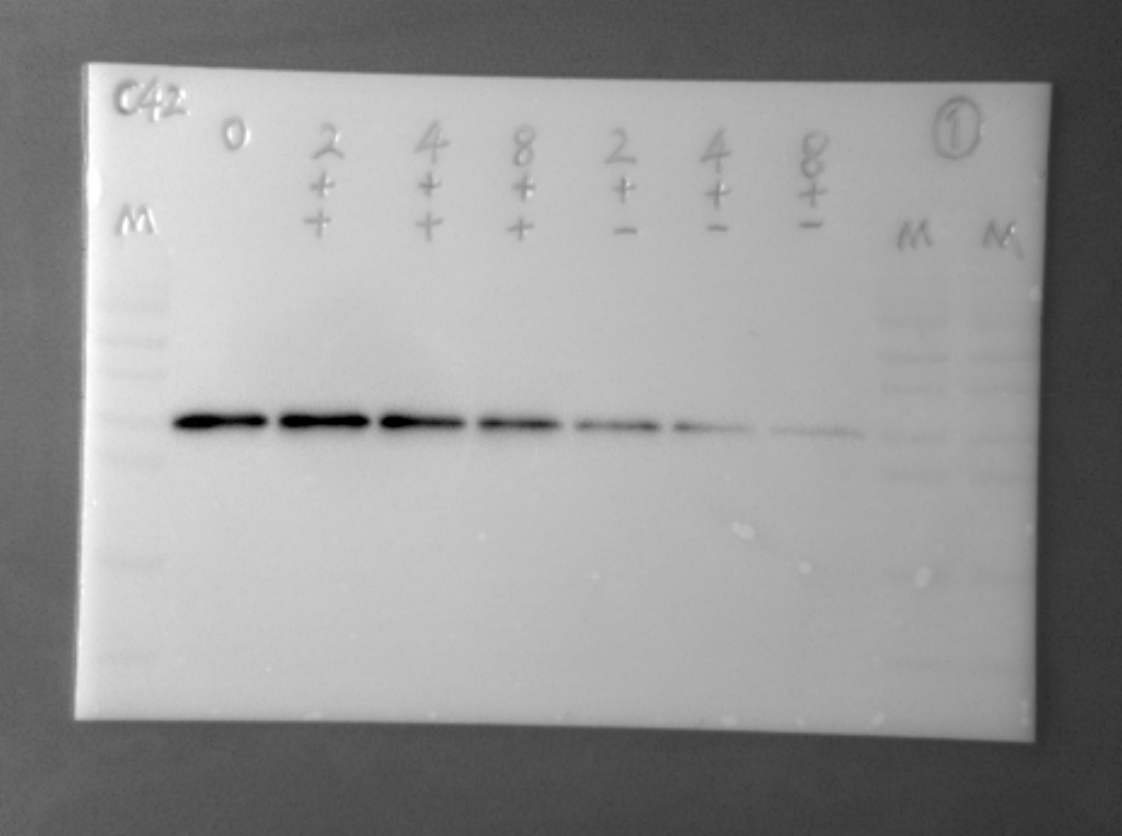

Supplement: Supplementary file 1 — Full and uncropped western blots [file 41419_2025_7809_MOESM1_ESM.zip › Full and uncropped western blots/Fig5C/C4-2/film1/SLC7A11-Merge.tif]

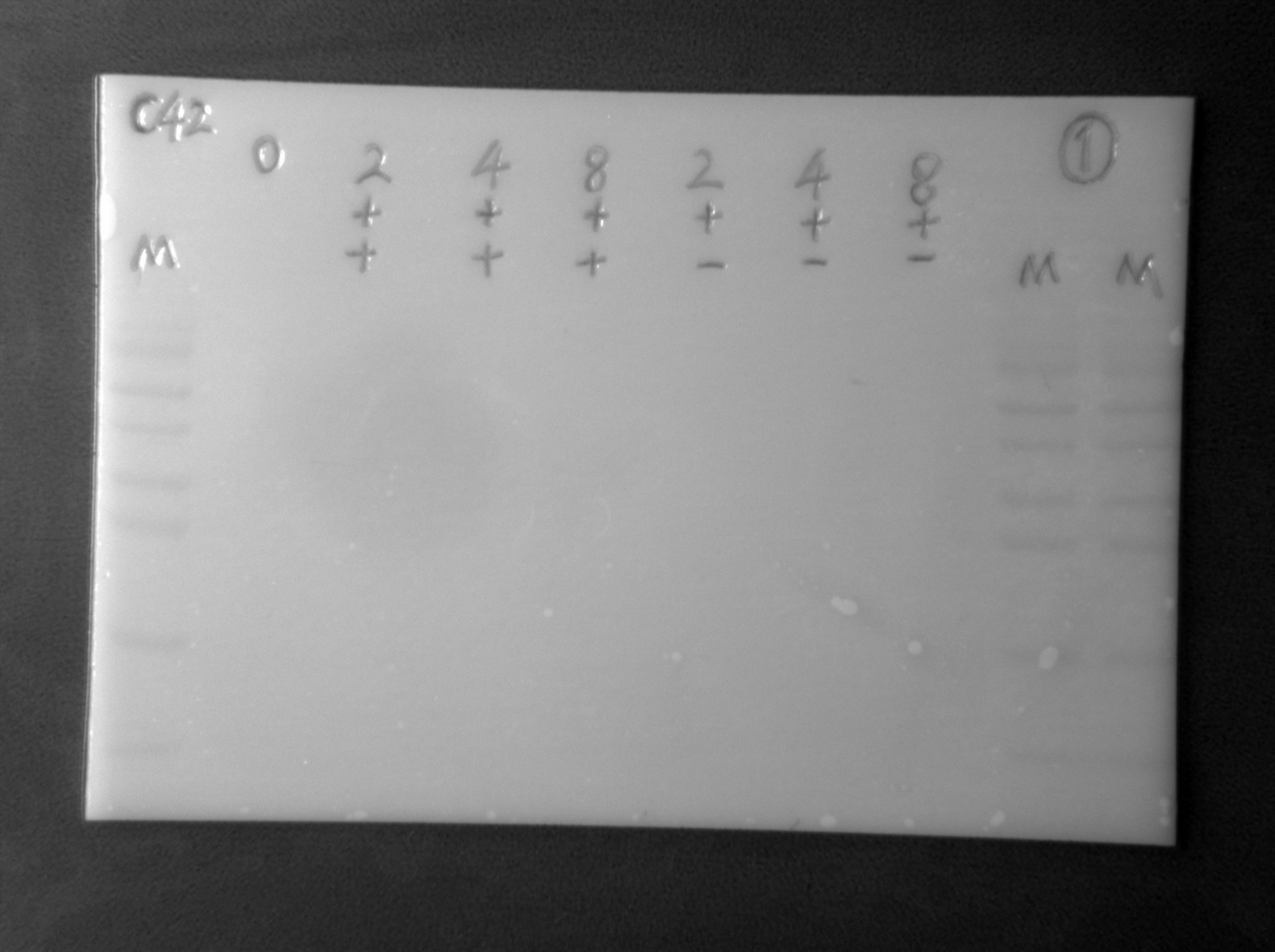

Supplement: Supplementary file 1 — Full and uncropped western blots [file 41419_2025_7809_MOESM1_ESM.zip › Full and uncropped western blots/Fig5C/C4-2/film1/SLC7A11-picture of film.tif]

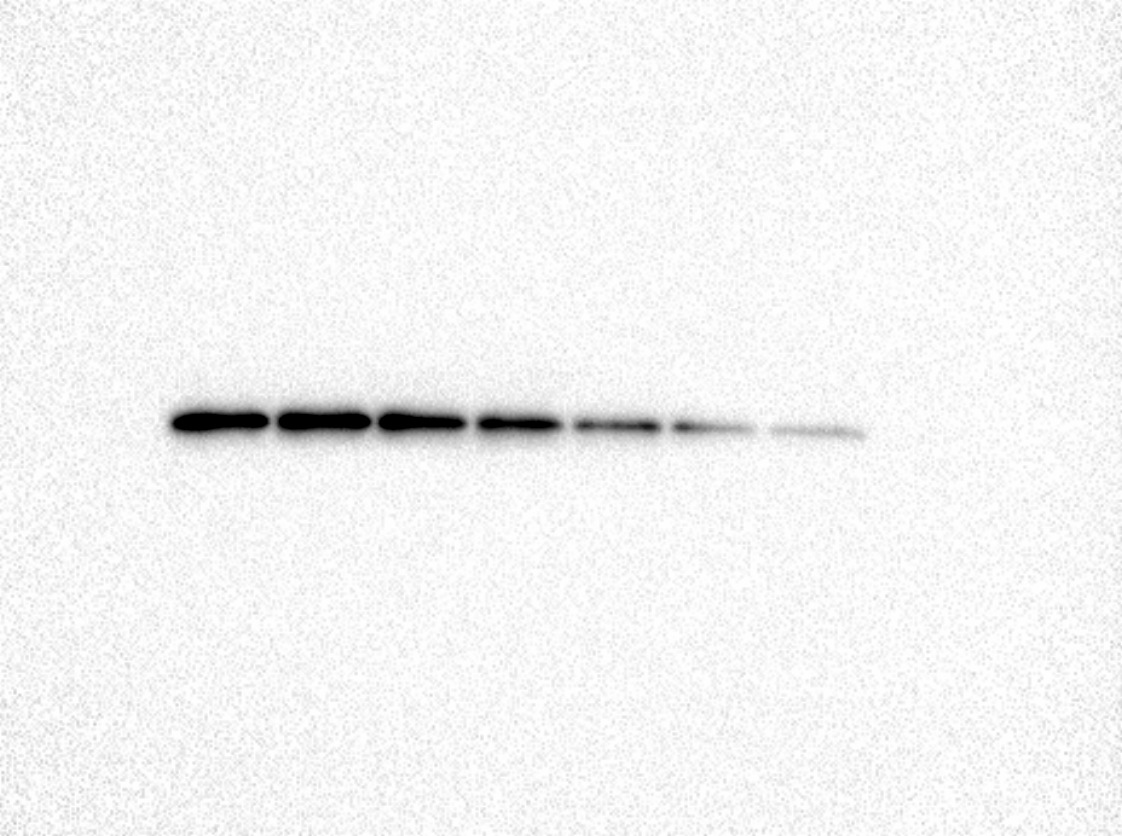

Supplement: Supplementary file 1 — Full and uncropped western blots [file 41419_2025_7809_MOESM1_ESM.zip › Full and uncropped western blots/Fig5C/C4-2/film1/SLC7A11.tif]

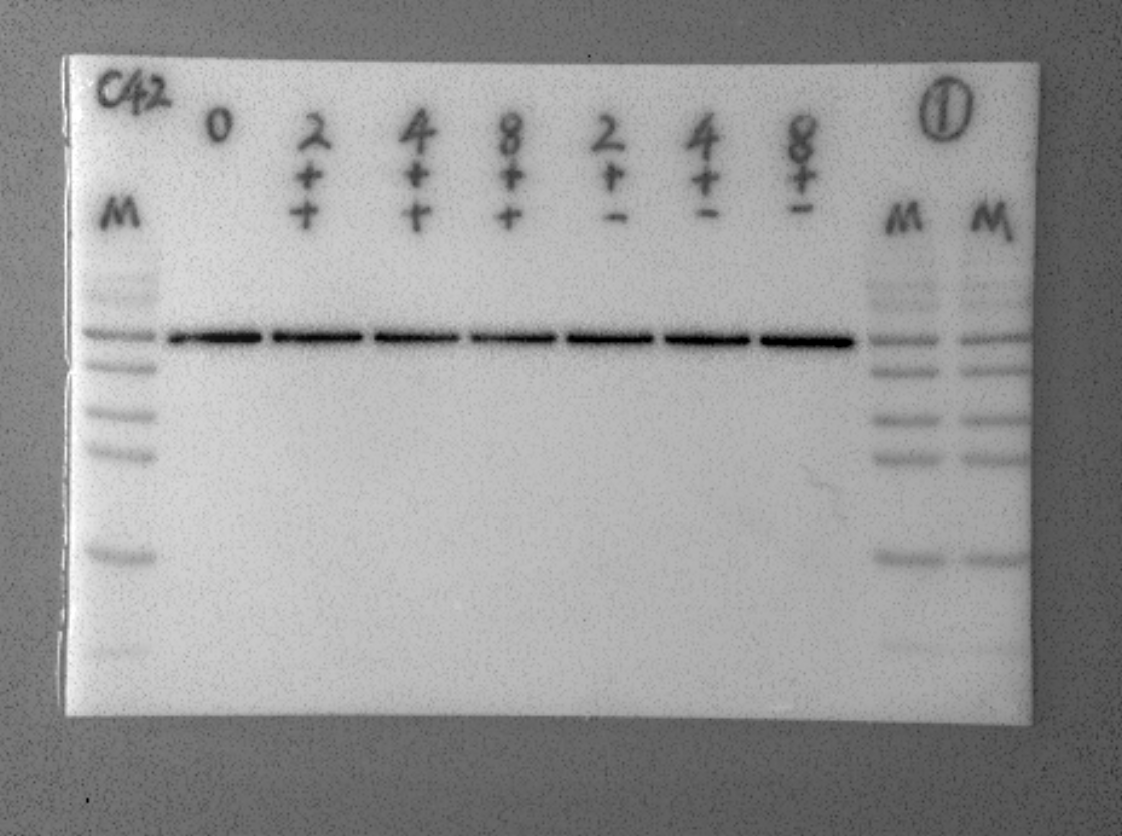

Supplement: Supplementary file 1 — Full and uncropped western blots [file 41419_2025_7809_MOESM1_ESM.zip › Full and uncropped western blots/Fig5C/C4-2/film1/Tubulin-Merge.tif]

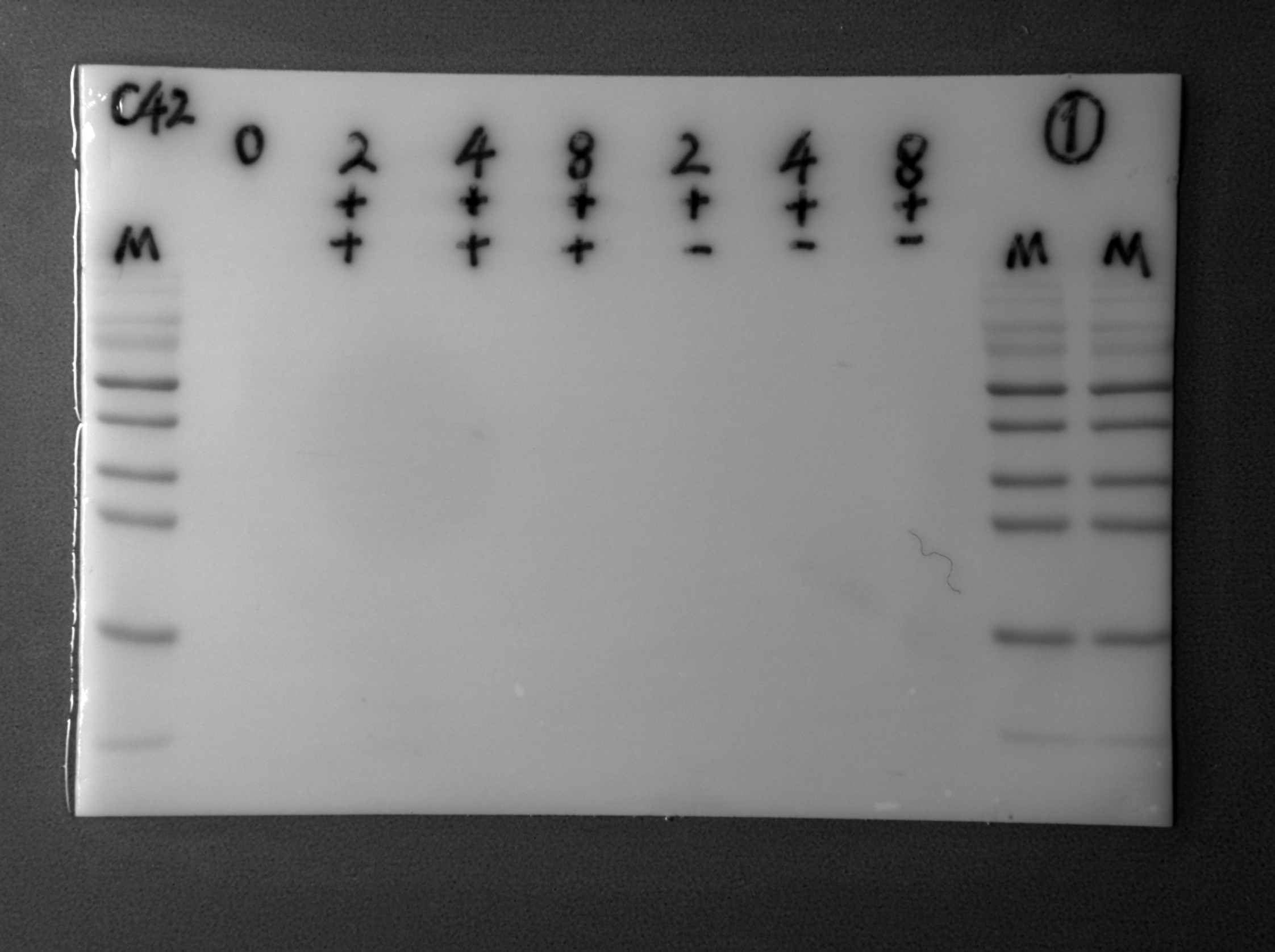

Supplement: Supplementary file 1 — Full and uncropped western blots [file 41419_2025_7809_MOESM1_ESM.zip › Full and uncropped western blots/Fig5C/C4-2/film1/Tubulin-picture of film.tif]

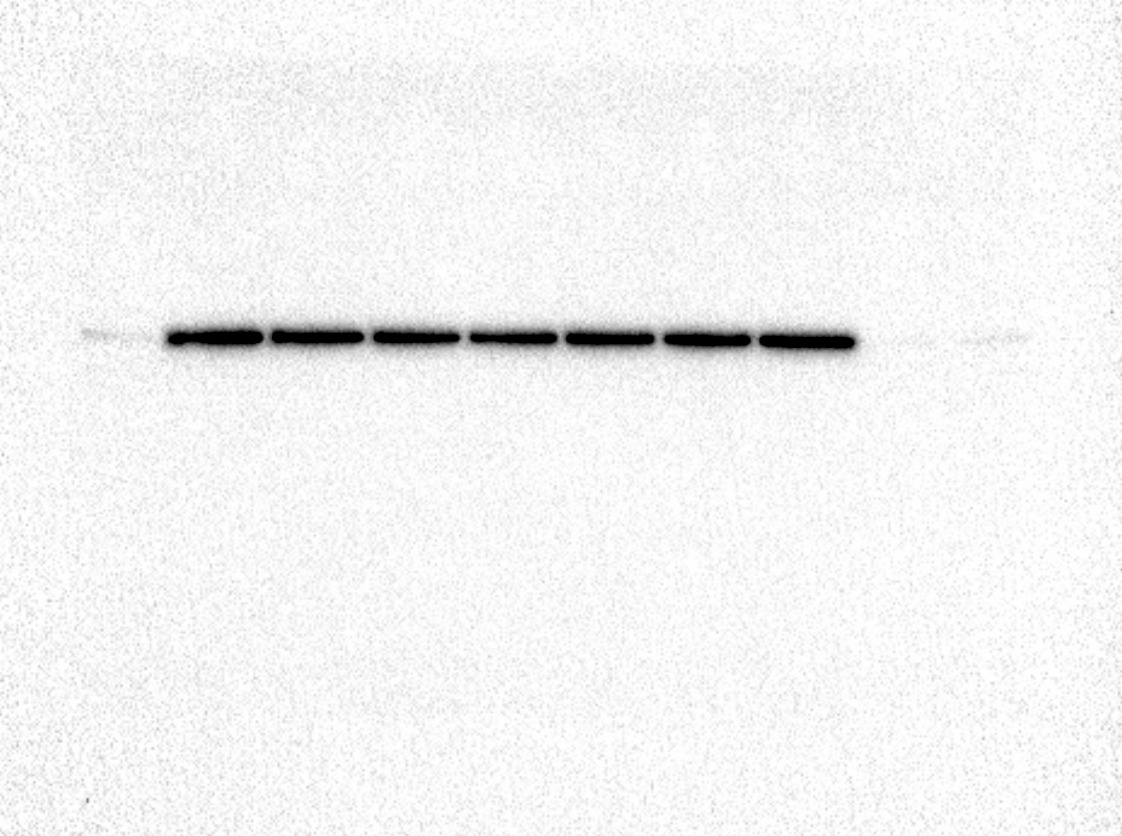

Supplement: Supplementary file 1 — Full and uncropped western blots [file 41419_2025_7809_MOESM1_ESM.zip › Full and uncropped western blots/Fig5C/C4-2/film1/Tubulin.tif]

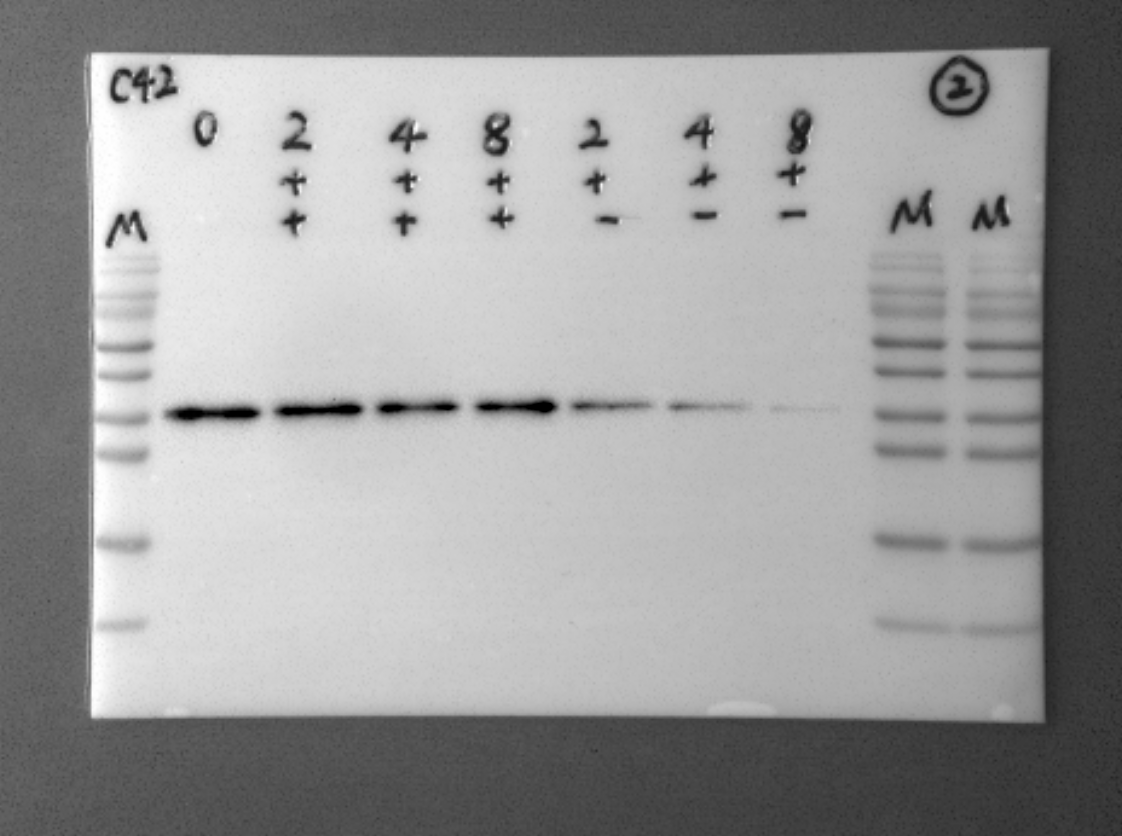

Supplement: Supplementary file 1 — Full and uncropped western blots [file 41419_2025_7809_MOESM1_ESM.zip › Full and uncropped western blots/Fig5C/C4-2/film2/SLC7A11-Merge.tif]

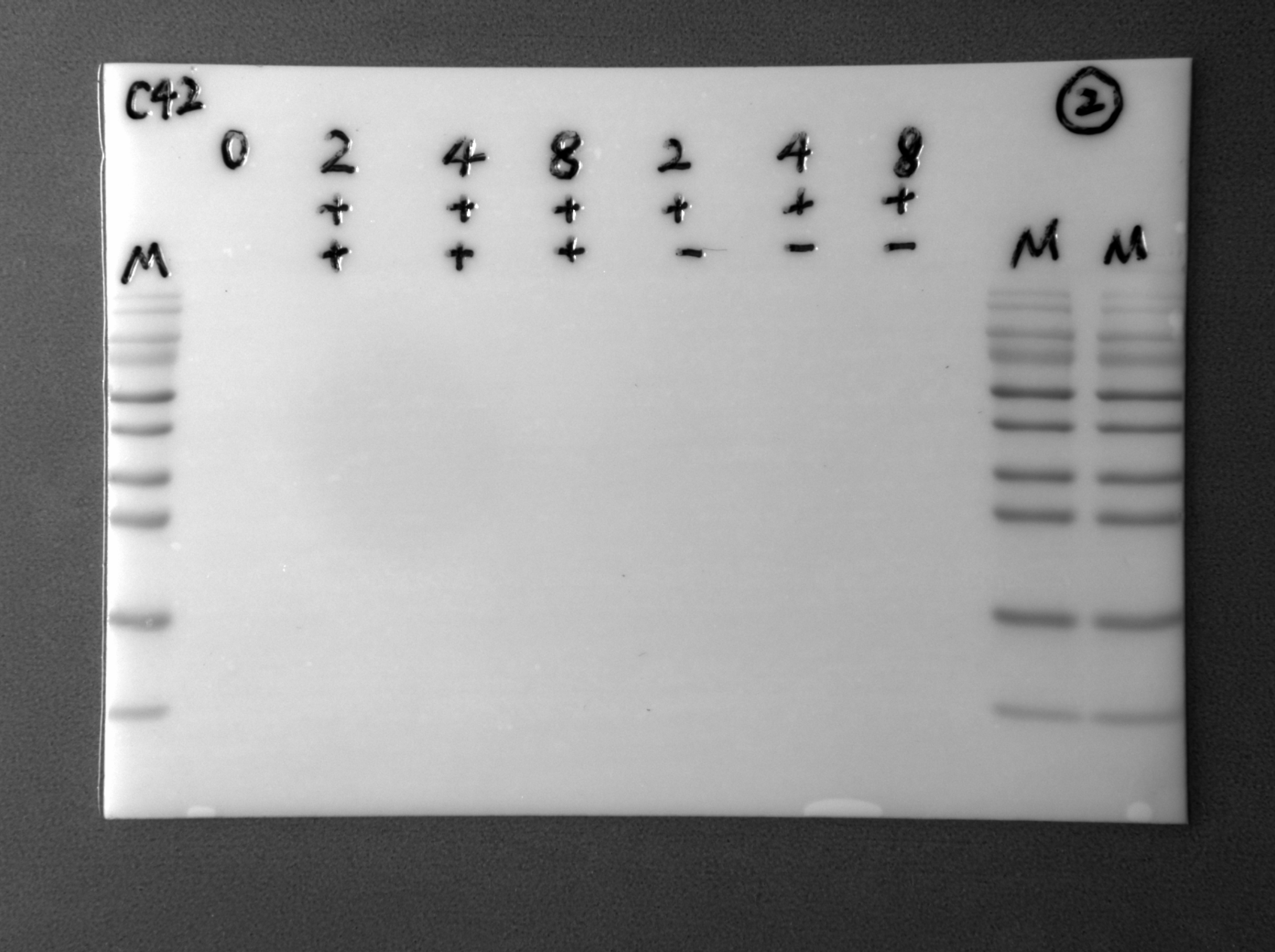

Supplement: Supplementary file 1 — Full and uncropped western blots [file 41419_2025_7809_MOESM1_ESM.zip › Full and uncropped western blots/Fig5C/C4-2/film2/SLC7A11-picture of film.tif]

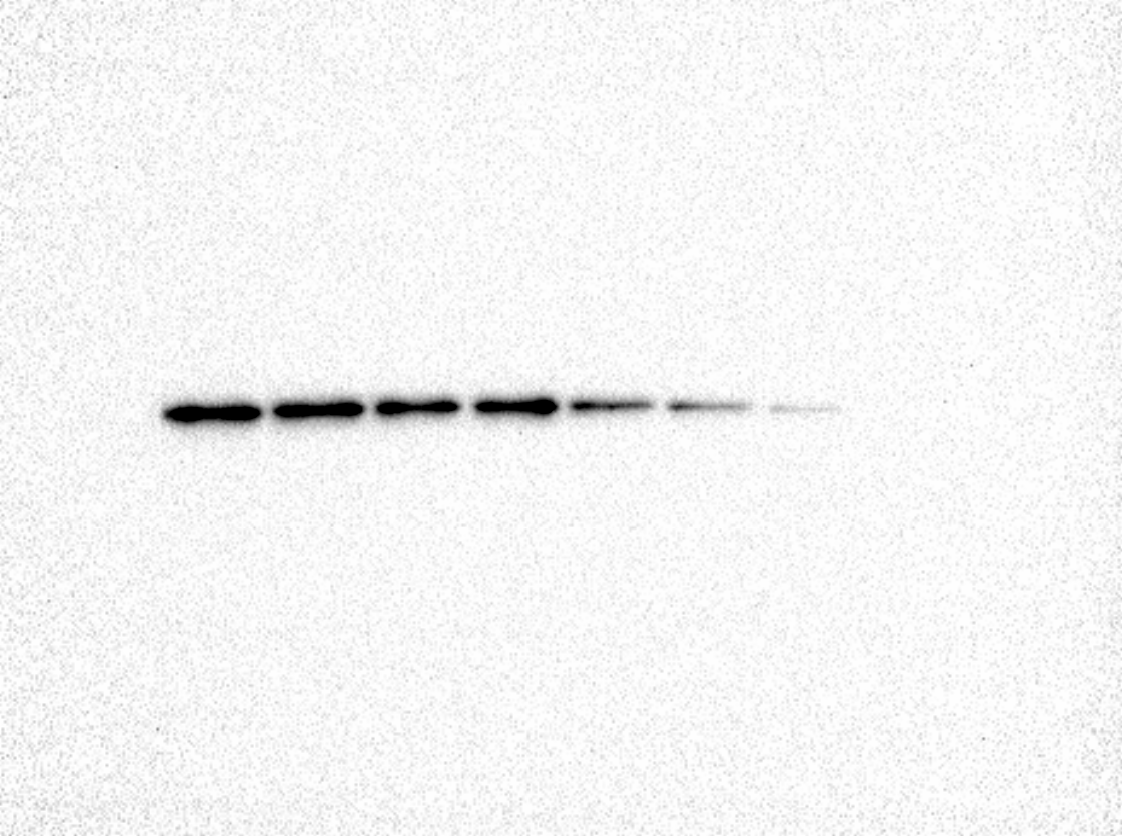

Supplement: Supplementary file 1 — Full and uncropped western blots [file 41419_2025_7809_MOESM1_ESM.zip › Full and uncropped western blots/Fig5C/C4-2/film2/SLC7A11.tif]

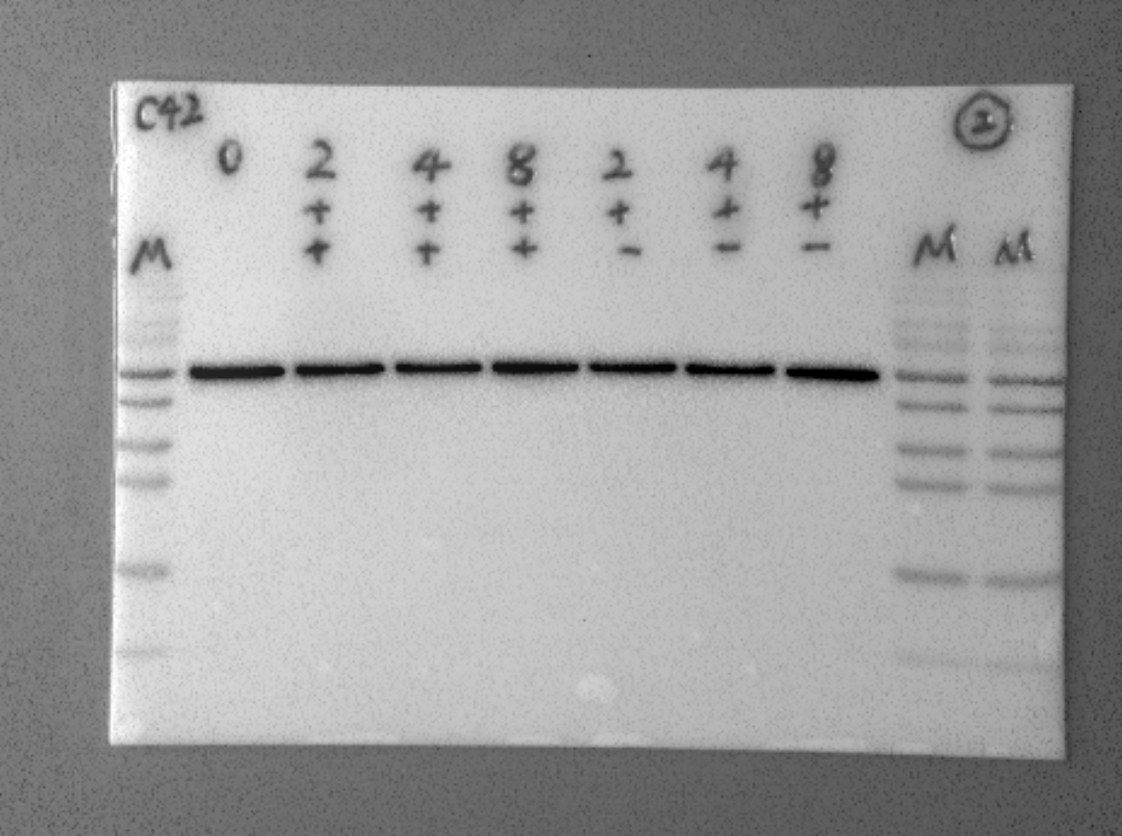

Supplement: Supplementary file 1 — Full and uncropped western blots [file 41419_2025_7809_MOESM1_ESM.zip › Full and uncropped western blots/Fig5C/C4-2/film2/Tubulin-Merge.tif]

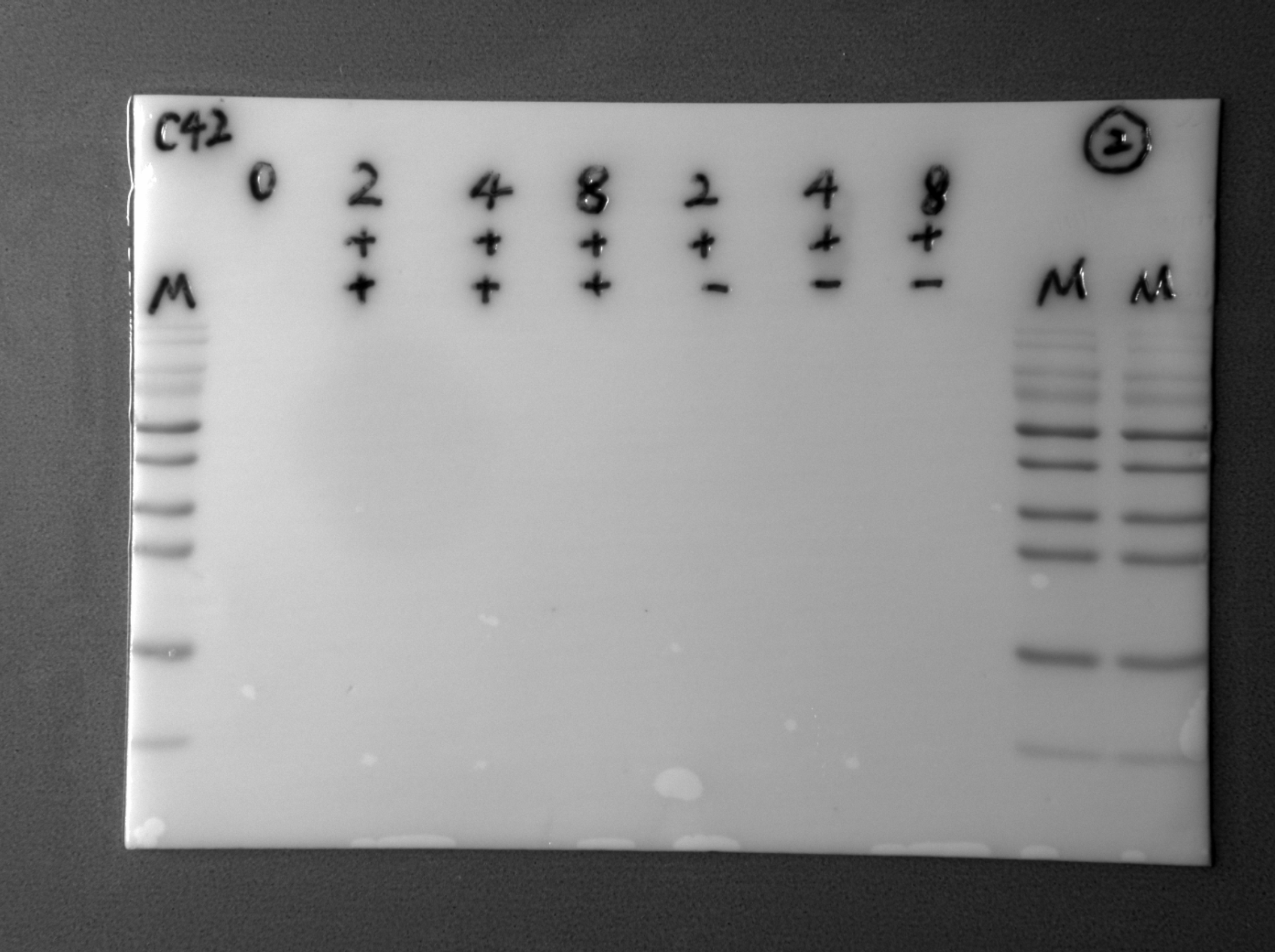

Supplement: Supplementary file 1 — Full and uncropped western blots [file 41419_2025_7809_MOESM1_ESM.zip › Full and uncropped western blots/Fig5C/C4-2/film2/Tubulin-picture of film.tif]

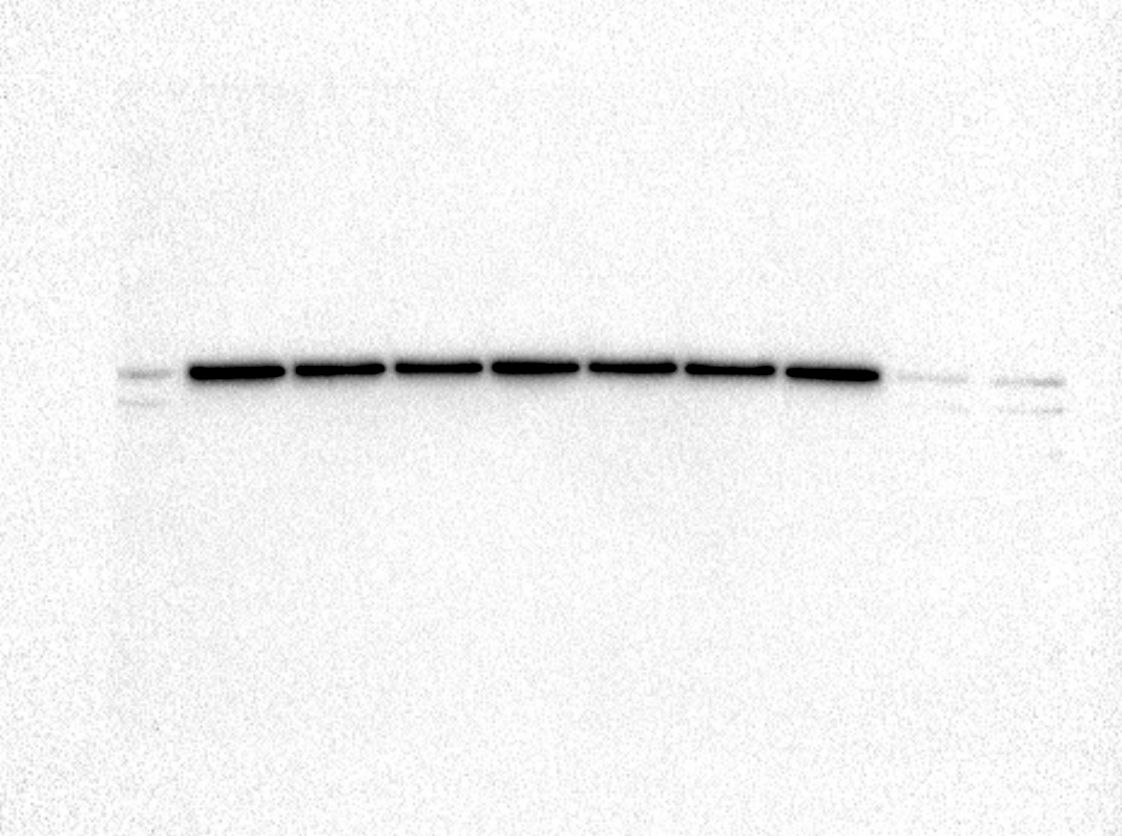

Supplement: Supplementary file 1 — Full and uncropped western blots [file 41419_2025_7809_MOESM1_ESM.zip › Full and uncropped western blots/Fig5C/C4-2/film2/Tubulin.tif]

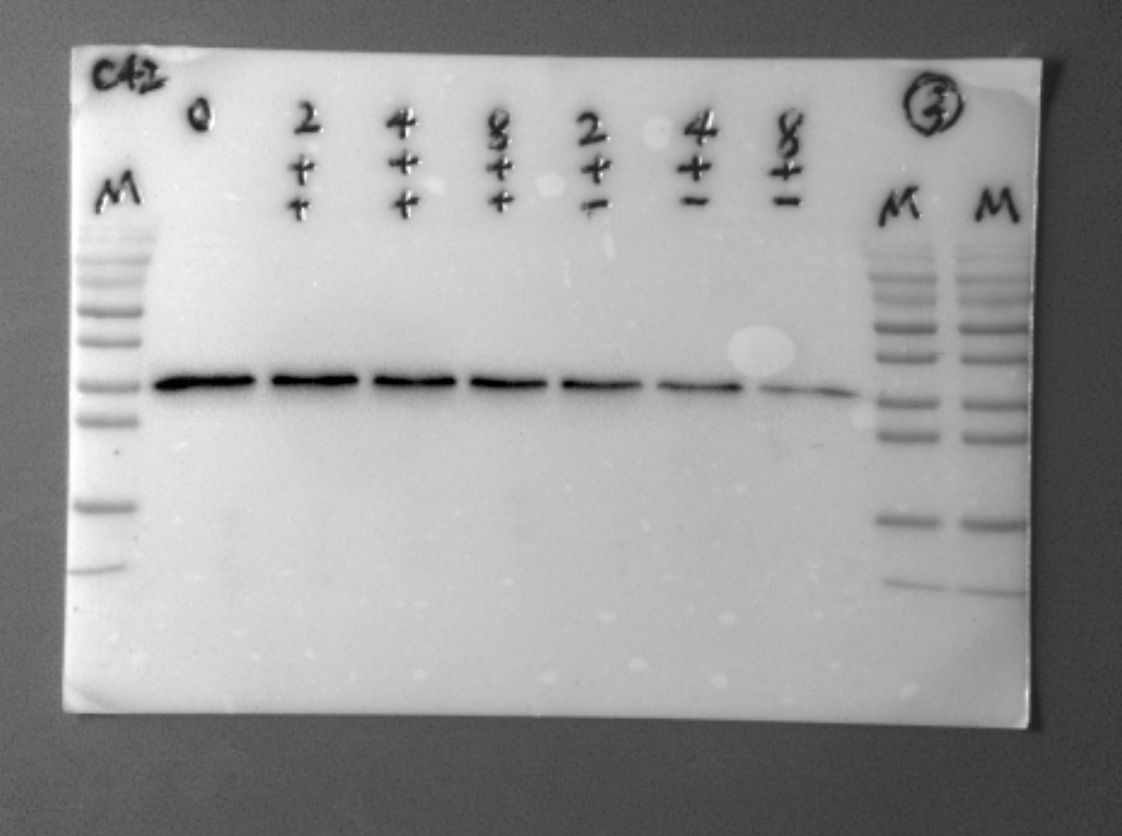

Supplement: Supplementary file 1 — Full and uncropped western blots [file 41419_2025_7809_MOESM1_ESM.zip › Full and uncropped western blots/Fig5C/C4-2/film3/SLC7A11-Merge.tif]

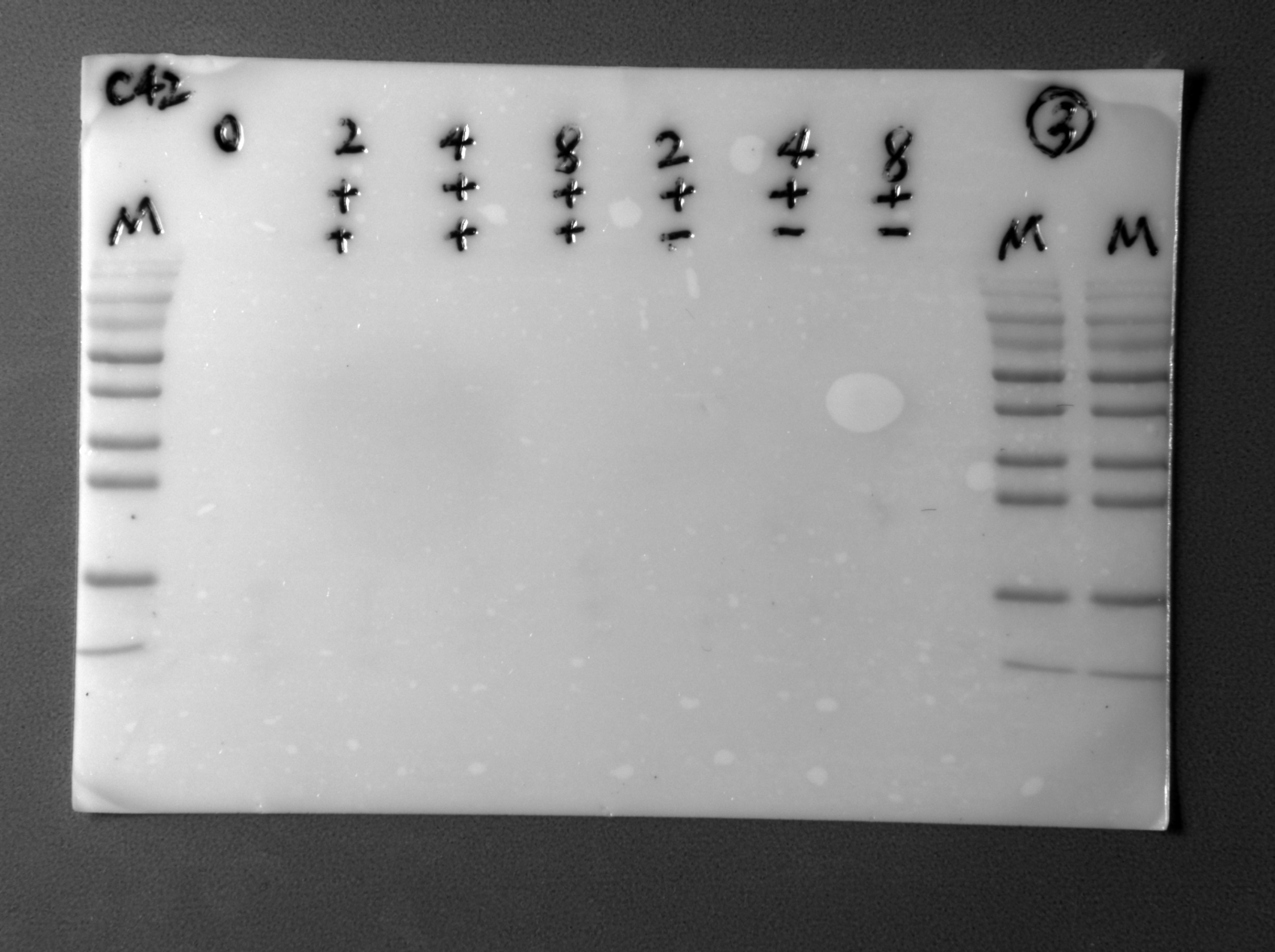

Supplement: Supplementary file 1 — Full and uncropped western blots [file 41419_2025_7809_MOESM1_ESM.zip › Full and uncropped western blots/Fig5C/C4-2/film3/SLC7A11-picture of film.tif]

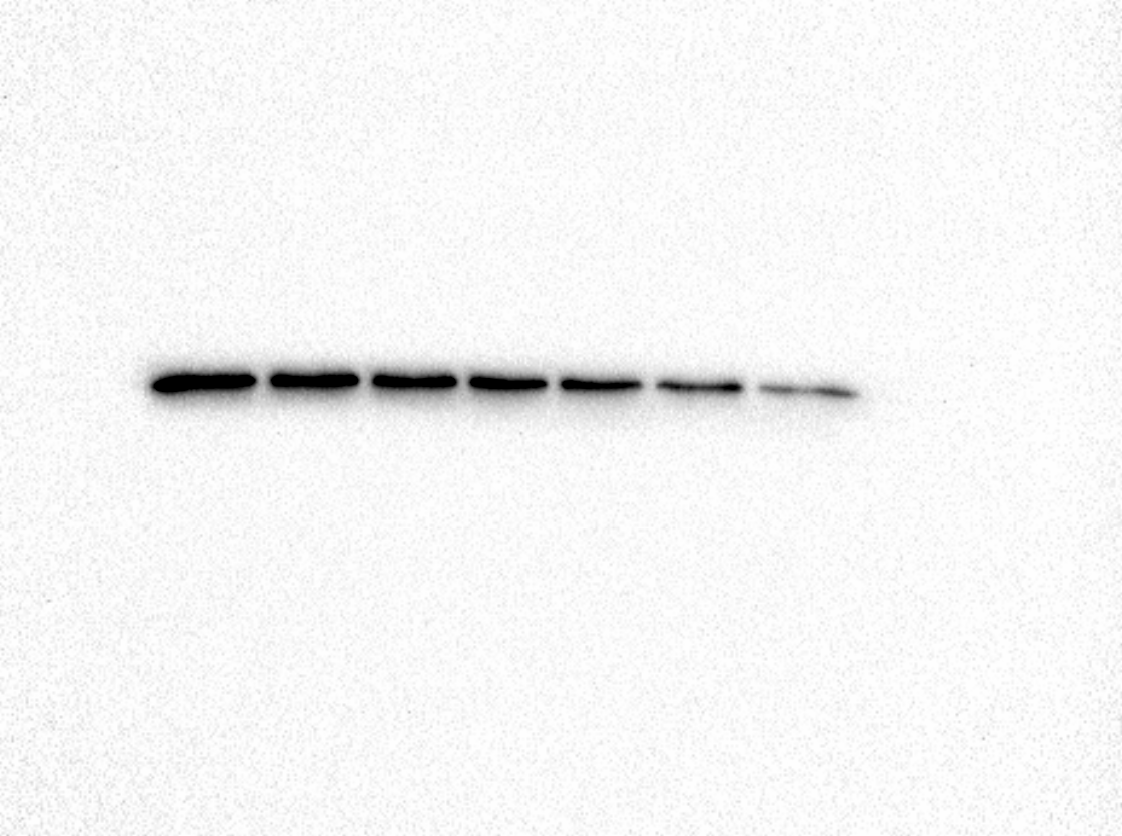

Supplement: Supplementary file 1 — Full and uncropped western blots [file 41419_2025_7809_MOESM1_ESM.zip › Full and uncropped western blots/Fig5C/C4-2/film3/SLC7A11.tif]

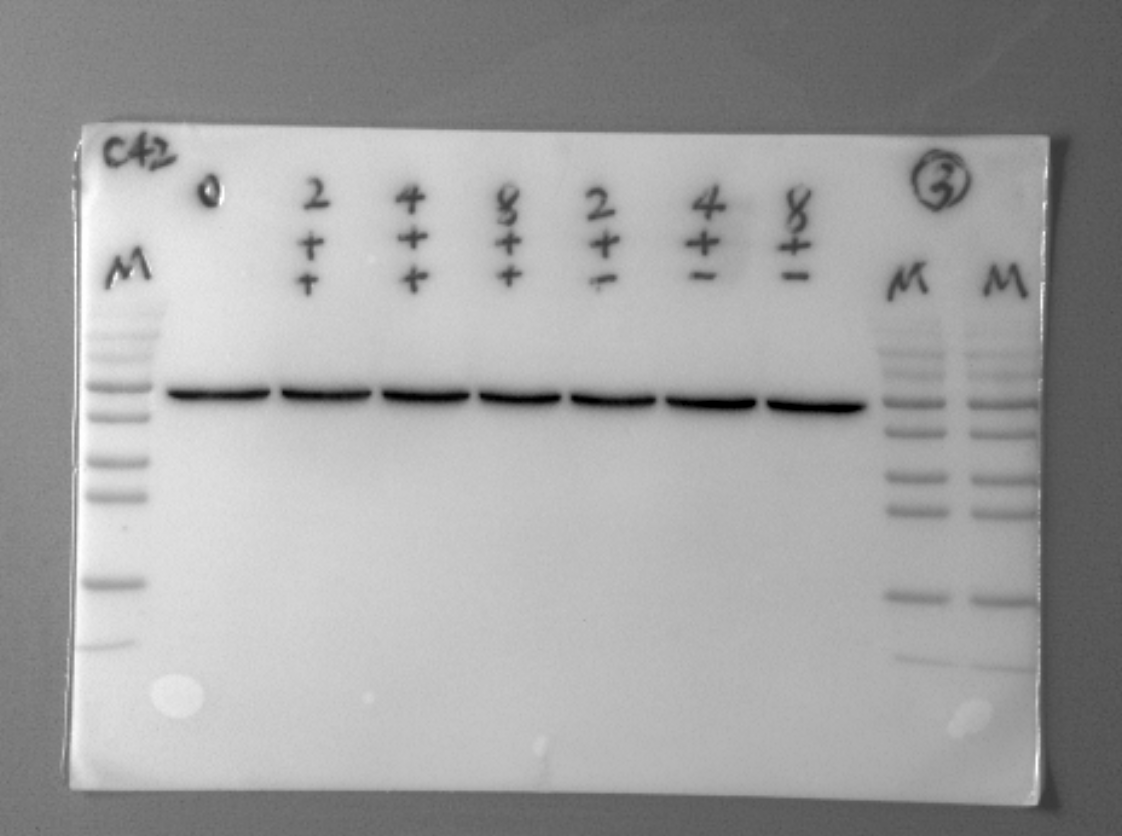

Supplement: Supplementary file 1 — Full and uncropped western blots [file 41419_2025_7809_MOESM1_ESM.zip › Full and uncropped western blots/Fig5C/C4-2/film3/Tubulin-Merge.tif]

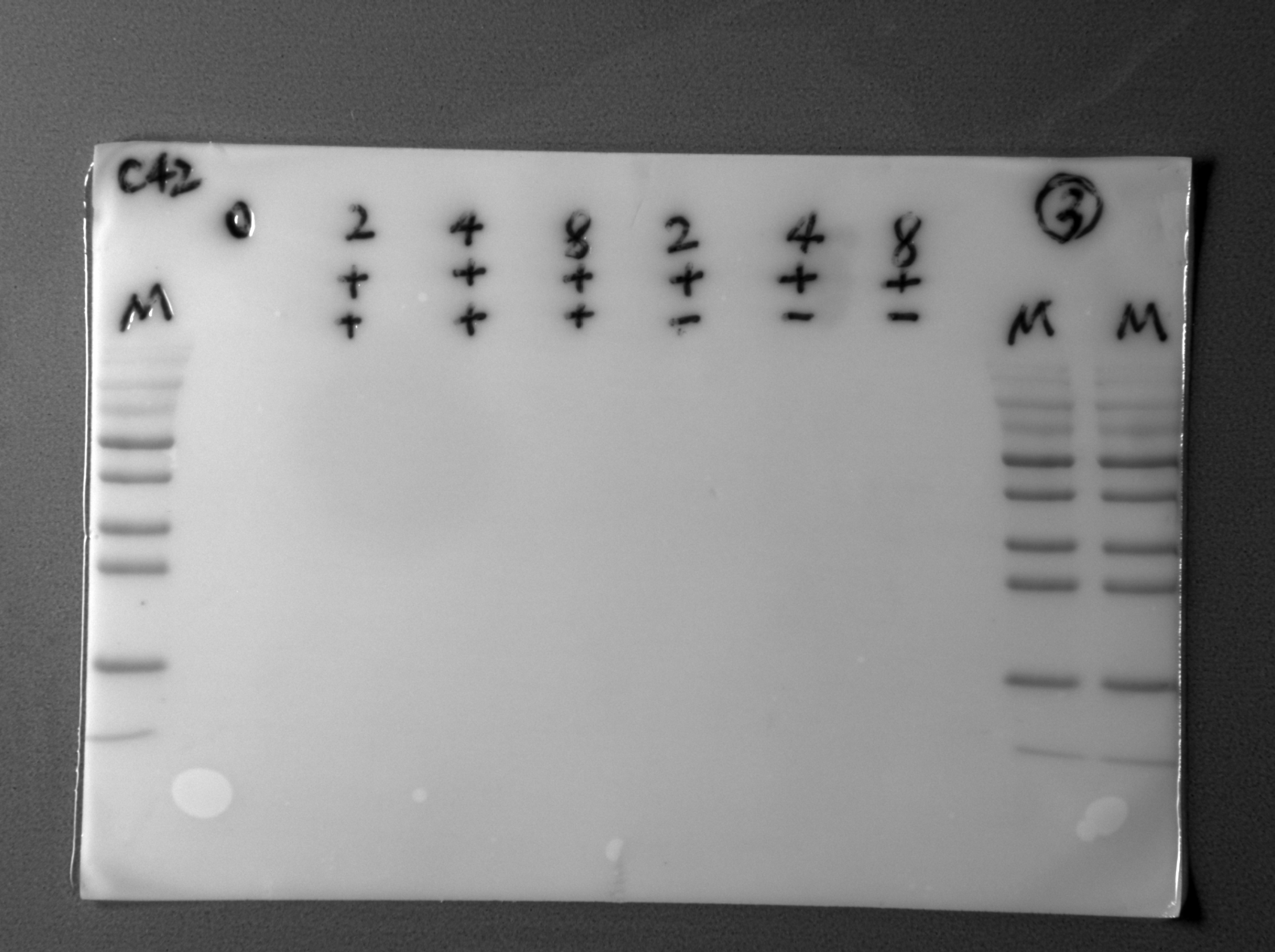

Supplement: Supplementary file 1 — Full and uncropped western blots [file 41419_2025_7809_MOESM1_ESM.zip › Full and uncropped western blots/Fig5C/C4-2/film3/Tubulin-picture of film.tif]

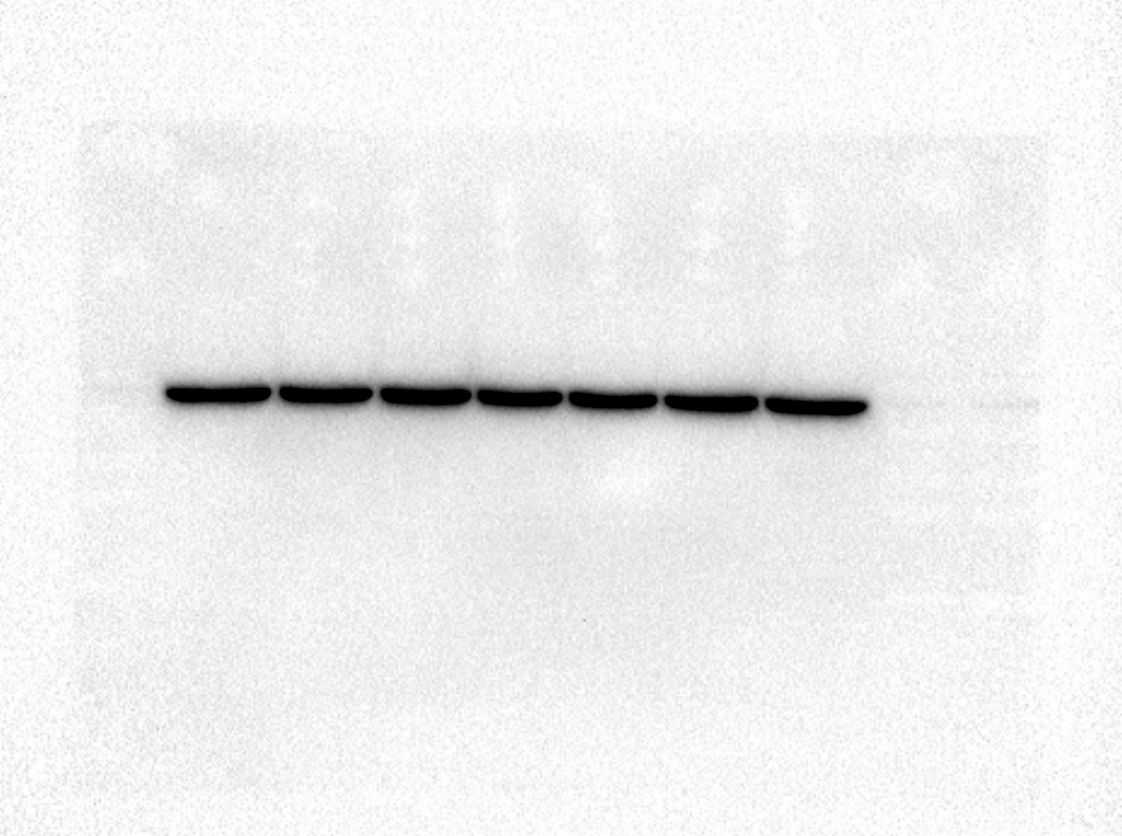

Supplement: Supplementary file 1 — Full and uncropped western blots [file 41419_2025_7809_MOESM1_ESM.zip › Full and uncropped western blots/Fig5C/C4-2/film3/Tubulin.tif]

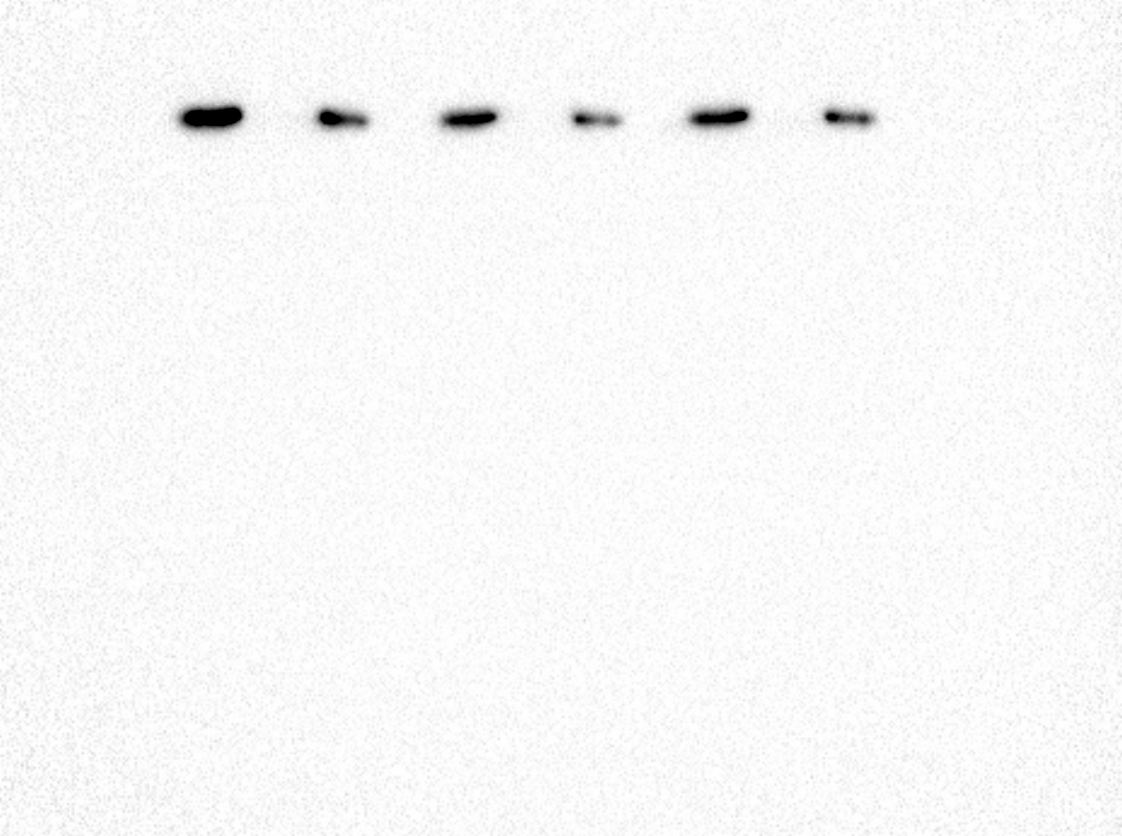

Supplement: Supplementary file 1 — Full and uncropped western blots [file 41419_2025_7809_MOESM1_ESM.zip › Full and uncropped western blots/Fig5I-co-ip/IP-NEDD4L/IB-NEDD4L/IP NEDD4L+IB NEDD4L 22RV1.tif]

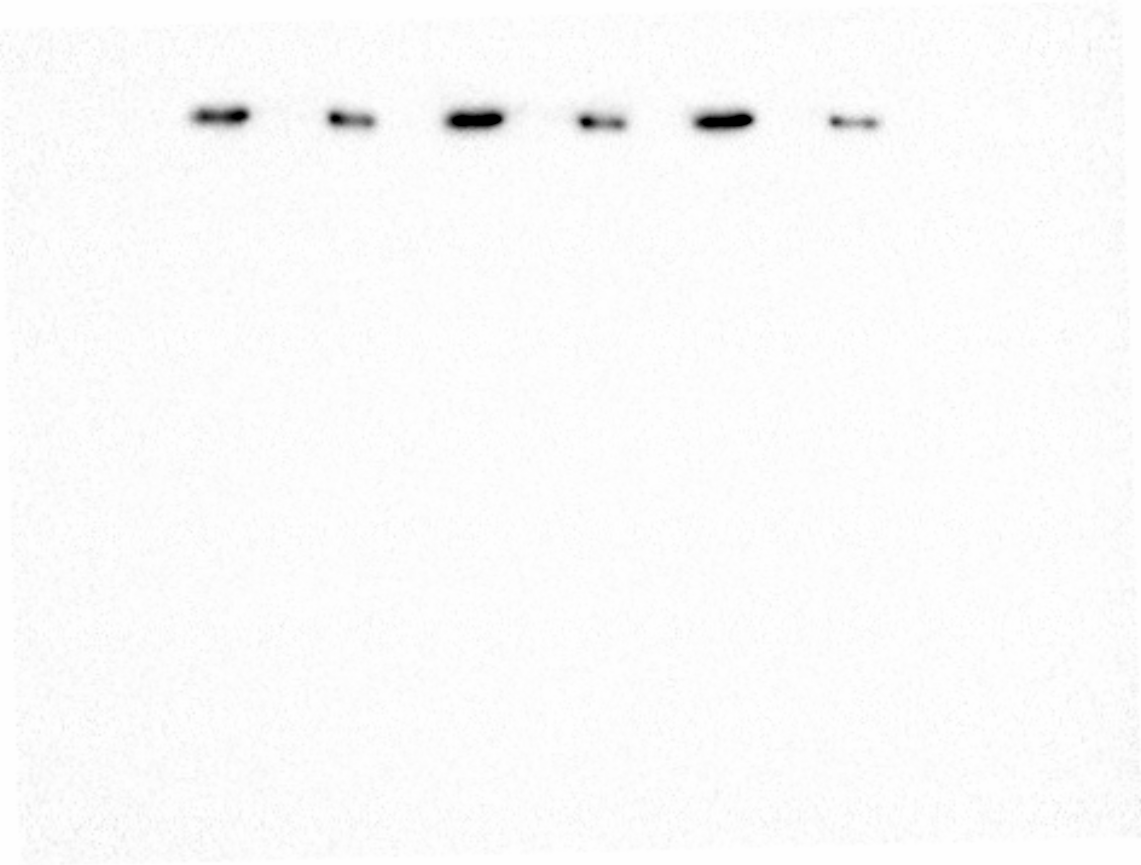

Supplement: Supplementary file 1 — Full and uncropped western blots [file 41419_2025_7809_MOESM1_ESM.zip › Full and uncropped western blots/Fig5I-co-ip/IP-NEDD4L/IB-NEDD4L/IP NEDD4L+IB NEDD4L C42.tif]

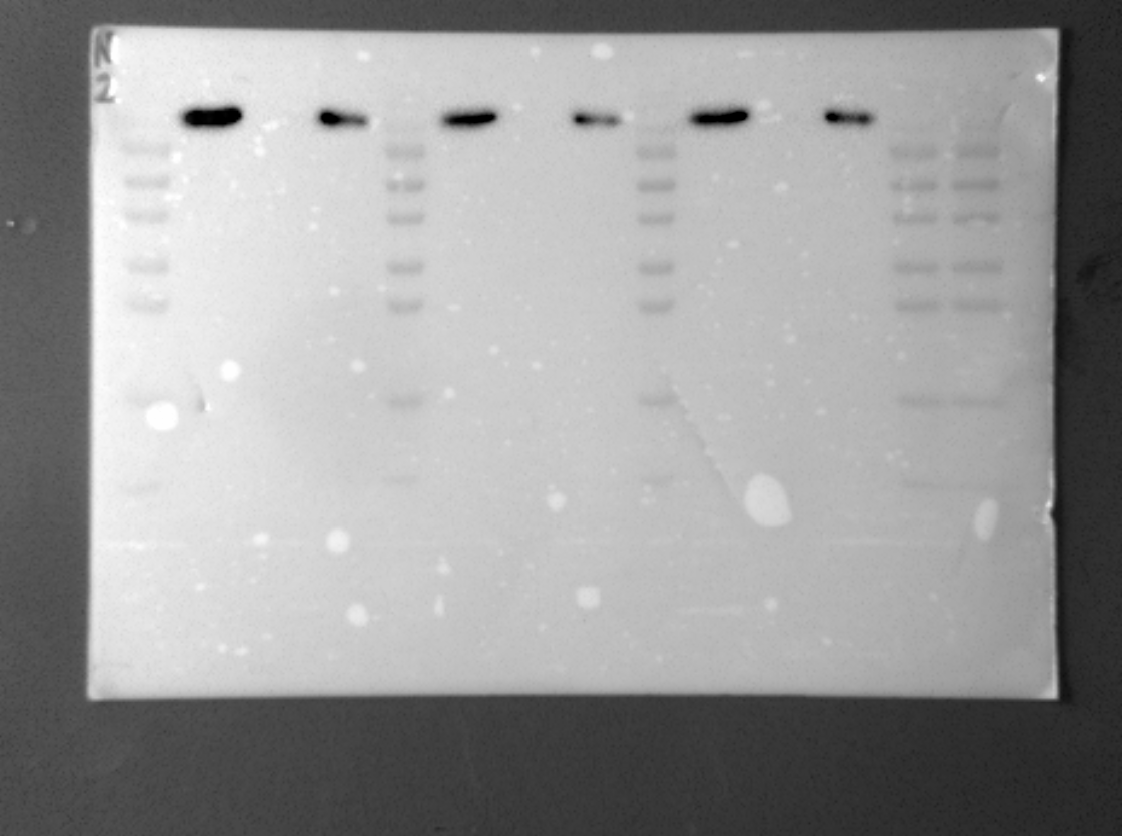

Supplement: Supplementary file 1 — Full and uncropped western blots [file 41419_2025_7809_MOESM1_ESM.zip › Full and uncropped western blots/Fig5I-co-ip/IP-NEDD4L/IB-NEDD4L/Merge 22RV1.tif]

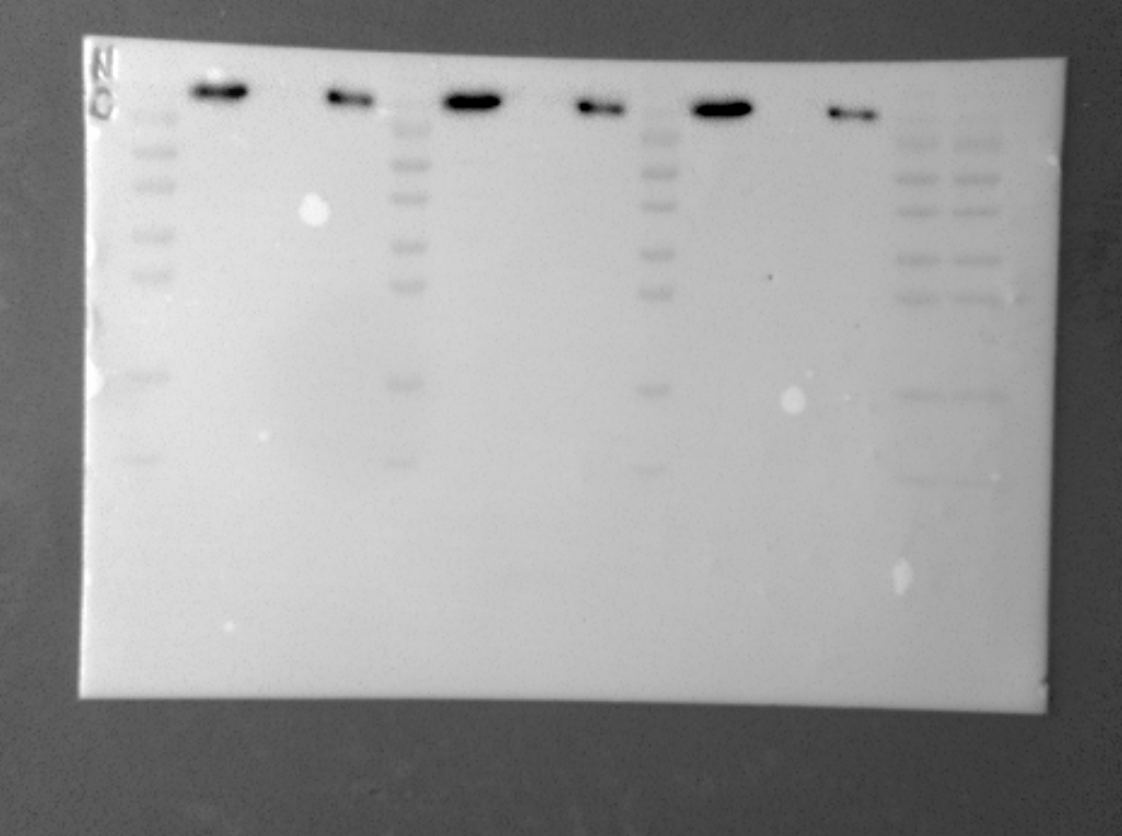

Supplement: Supplementary file 1 — Full and uncropped western blots [file 41419_2025_7809_MOESM1_ESM.zip › Full and uncropped western blots/Fig5I-co-ip/IP-NEDD4L/IB-NEDD4L/Merge C42.tif]

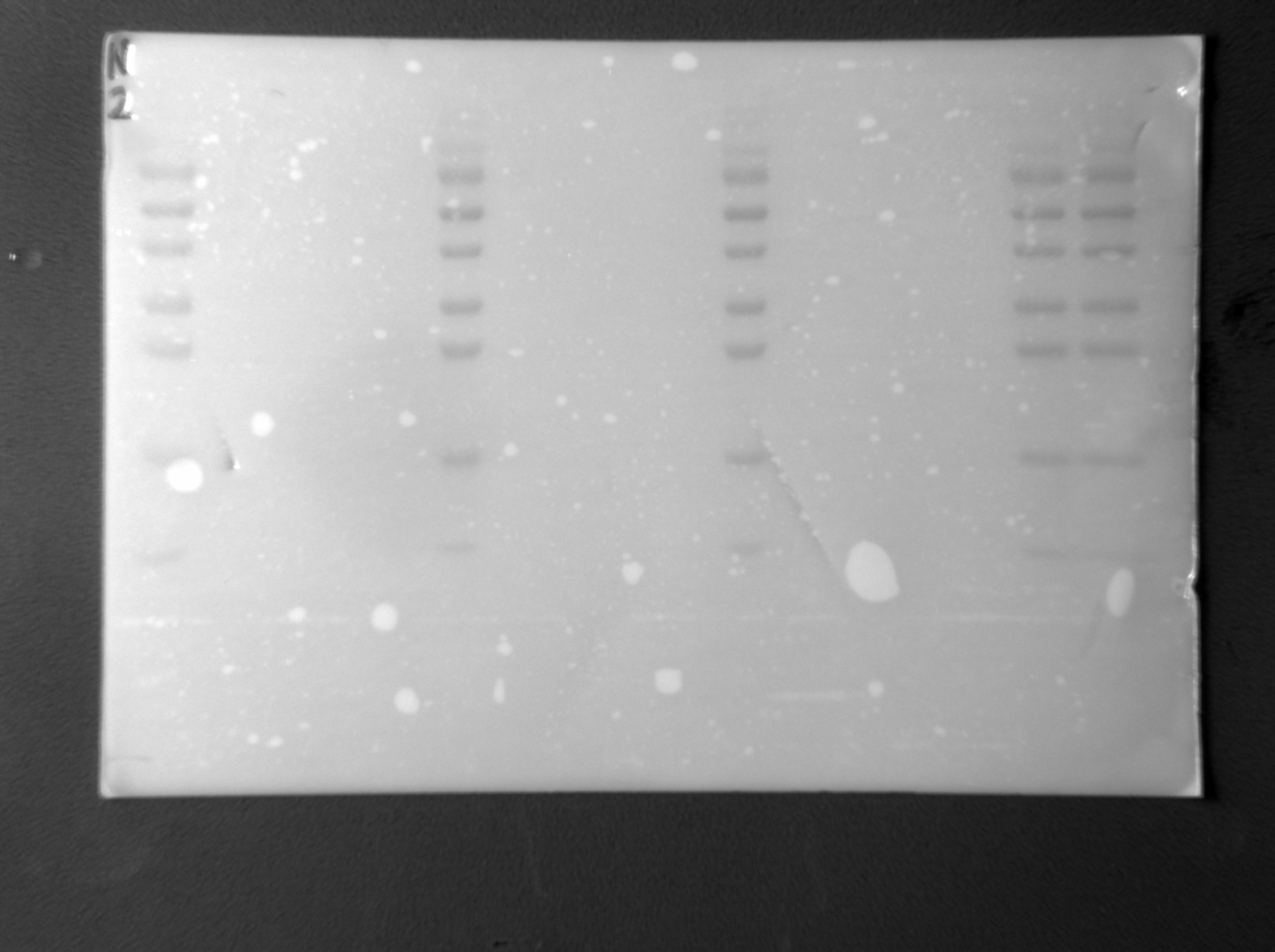

Supplement: Supplementary file 1 — Full and uncropped western blots [file 41419_2025_7809_MOESM1_ESM.zip › Full and uncropped western blots/Fig5I-co-ip/IP-NEDD4L/IB-NEDD4L/picture of film 22RV1.tif]

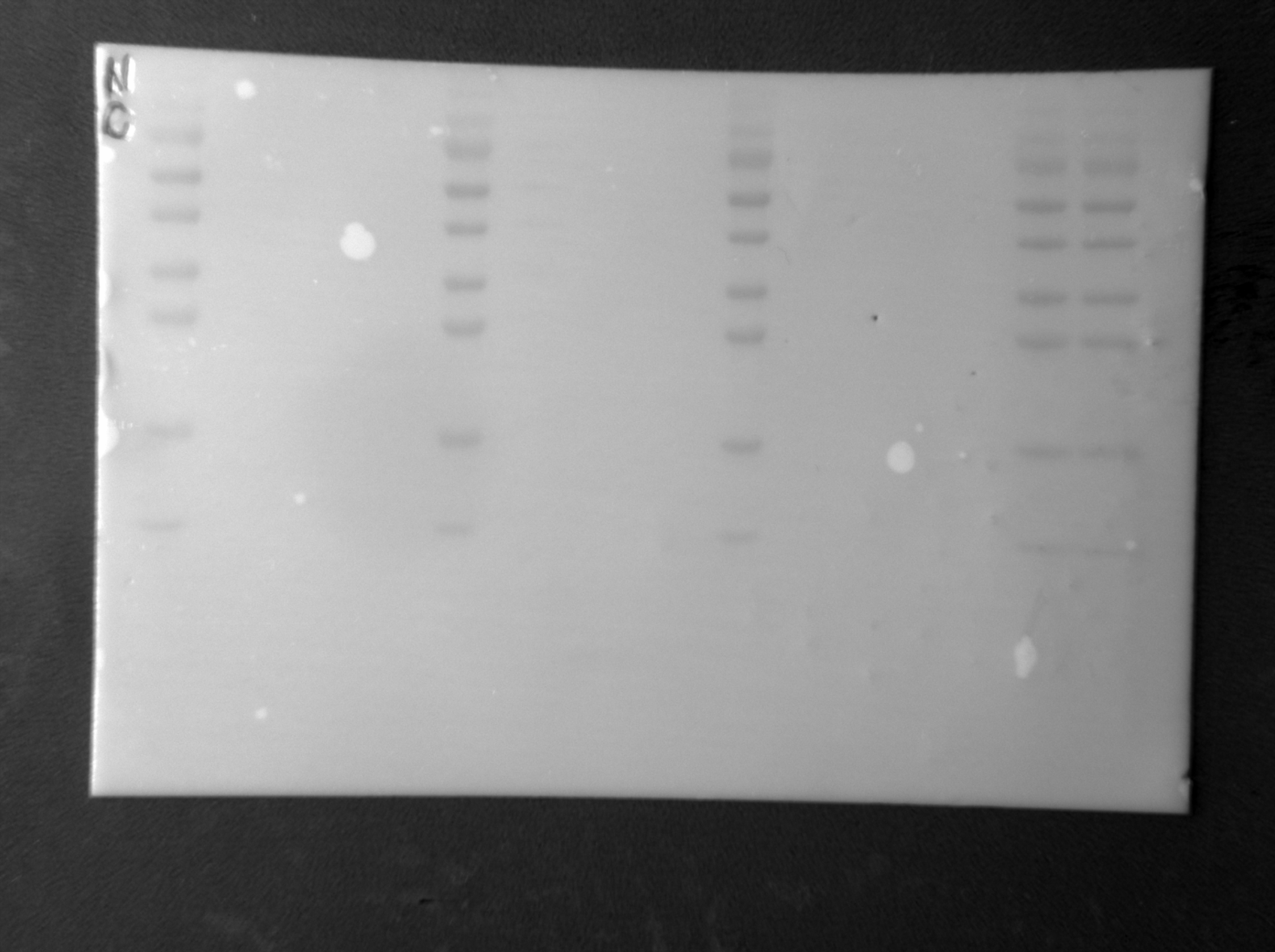

Supplement: Supplementary file 1 — Full and uncropped western blots [file 41419_2025_7809_MOESM1_ESM.zip › Full and uncropped western blots/Fig5I-co-ip/IP-NEDD4L/IB-NEDD4L/picture of film C42.tif]

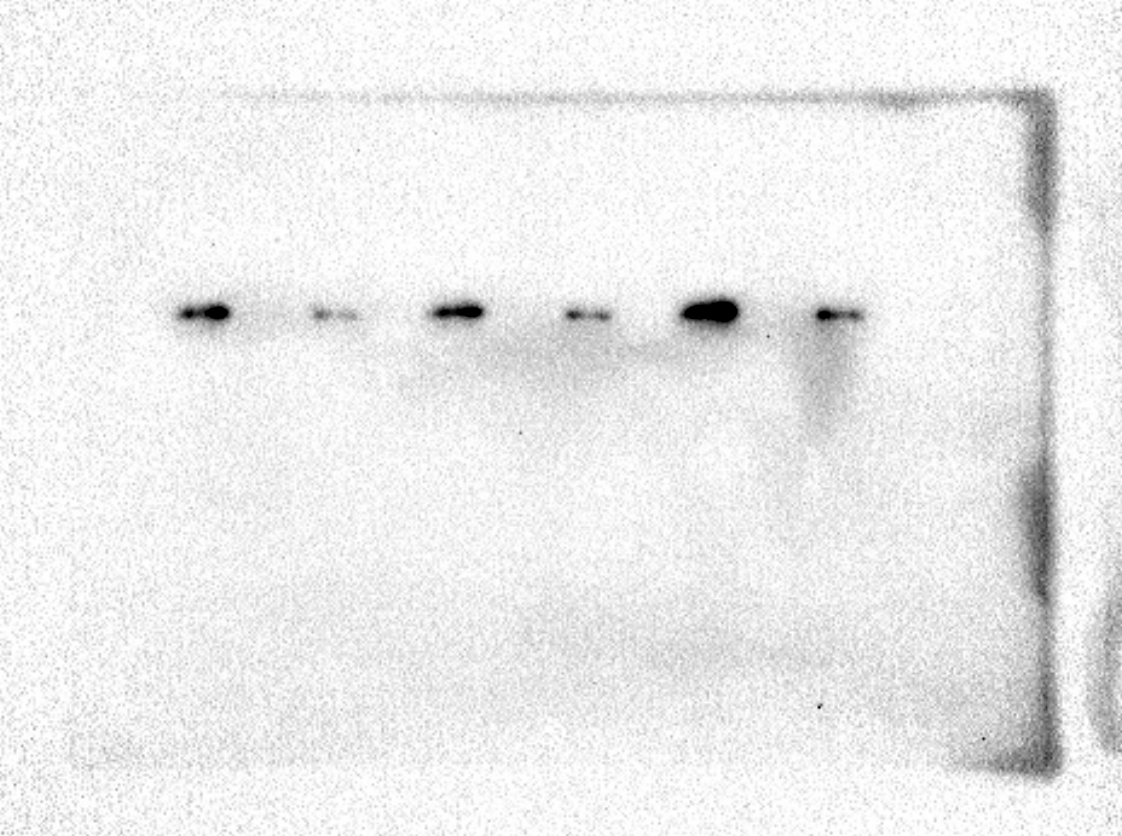

Supplement: Supplementary file 1 — Full and uncropped western blots [file 41419_2025_7809_MOESM1_ESM.zip › Full and uncropped western blots/Fig5I-co-ip/IP-NEDD4L/IB-SLC7A11/IP NEDD4L+IB SLC7A11 22Rv1.tif]

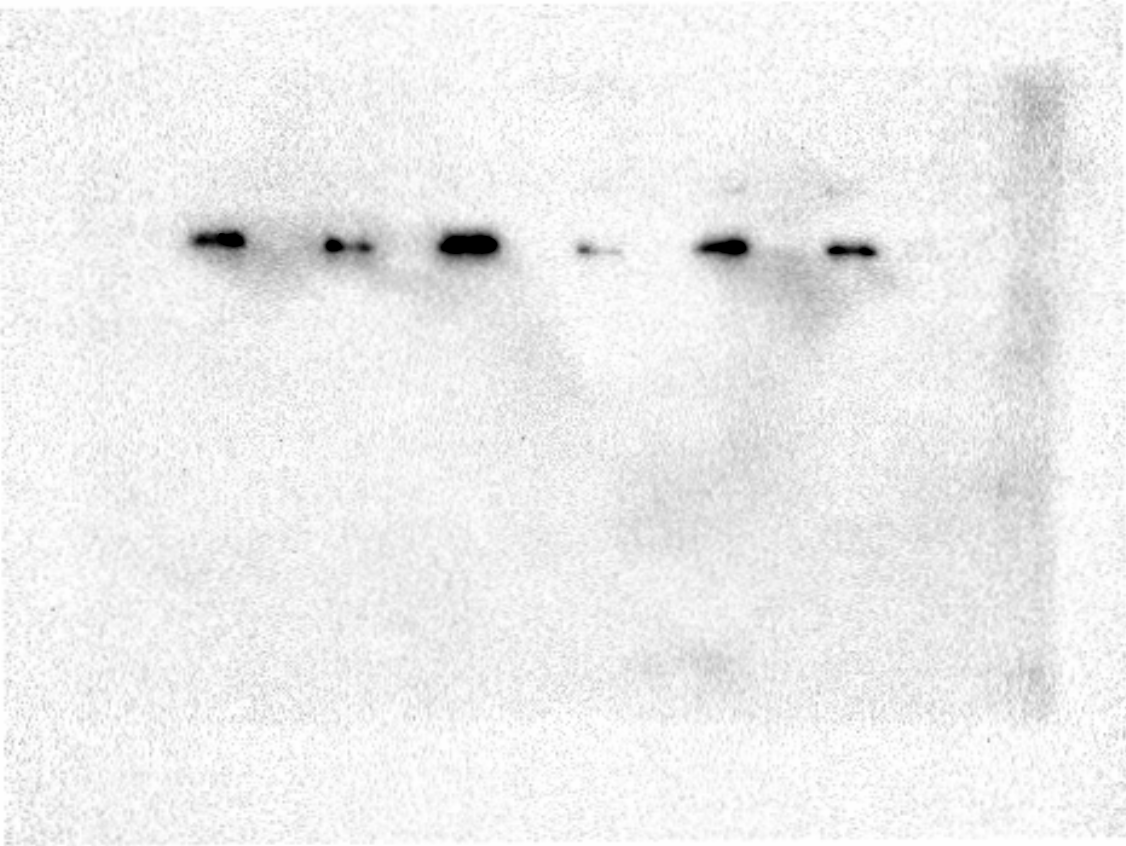

Supplement: Supplementary file 1 — Full and uncropped western blots [file 41419_2025_7809_MOESM1_ESM.zip › Full and uncropped western blots/Fig5I-co-ip/IP-NEDD4L/IB-SLC7A11/IP NEDD4L+IB SLC7A11 C42.tif]

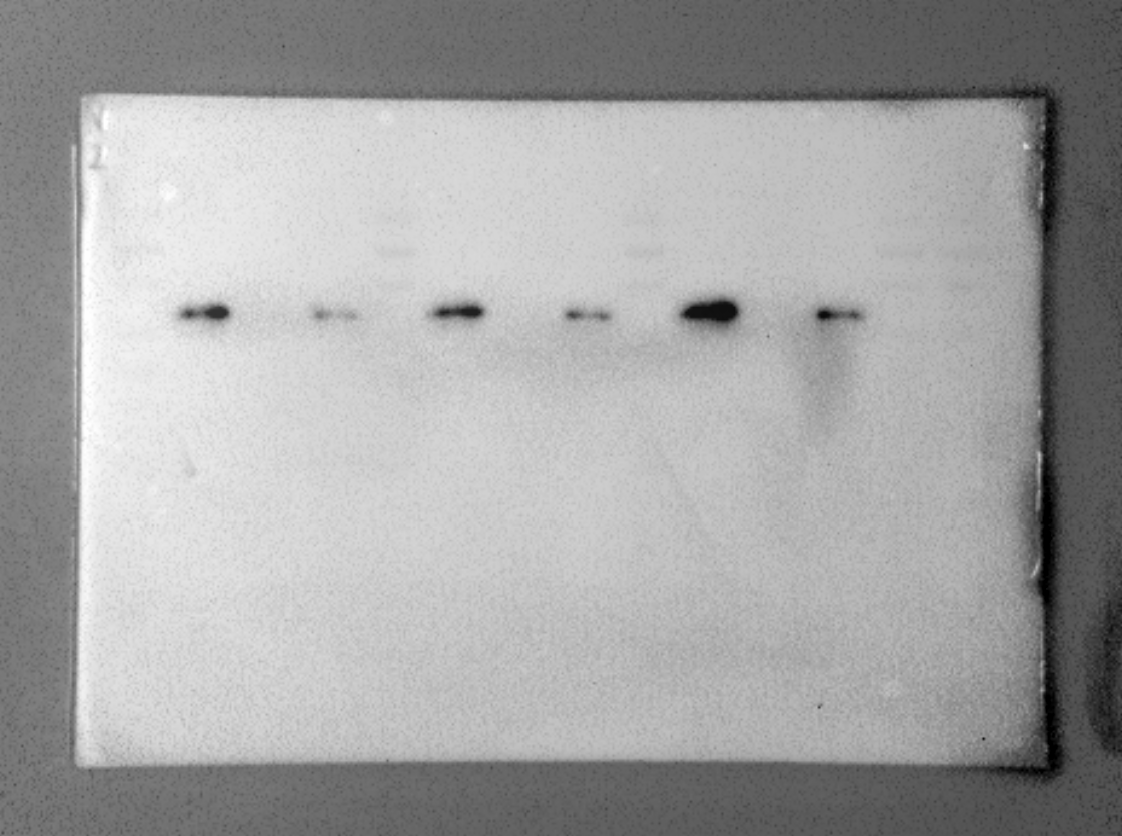

Supplement: Supplementary file 1 — Full and uncropped western blots [file 41419_2025_7809_MOESM1_ESM.zip › Full and uncropped western blots/Fig5I-co-ip/IP-NEDD4L/IB-SLC7A11/Merge 22Rv1.tif]

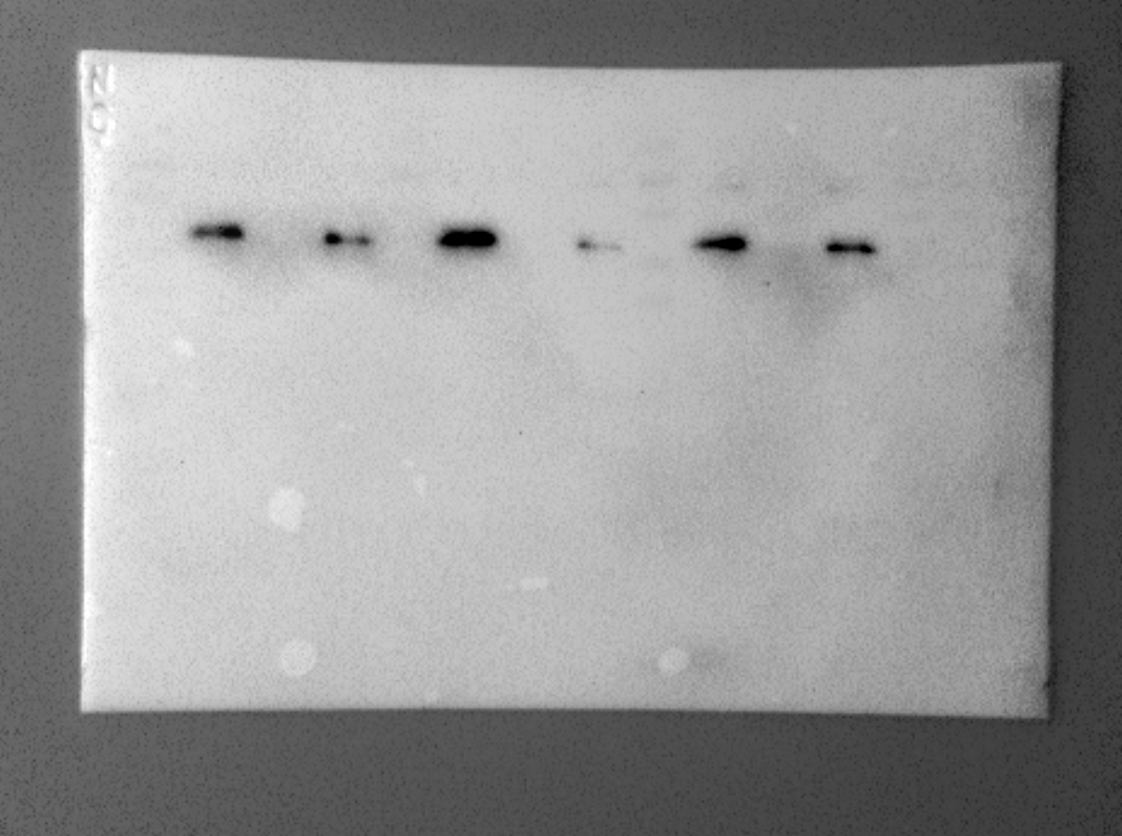

Supplement: Supplementary file 1 — Full and uncropped western blots [file 41419_2025_7809_MOESM1_ESM.zip › Full and uncropped western blots/Fig5I-co-ip/IP-NEDD4L/IB-SLC7A11/Merge C42.tif]

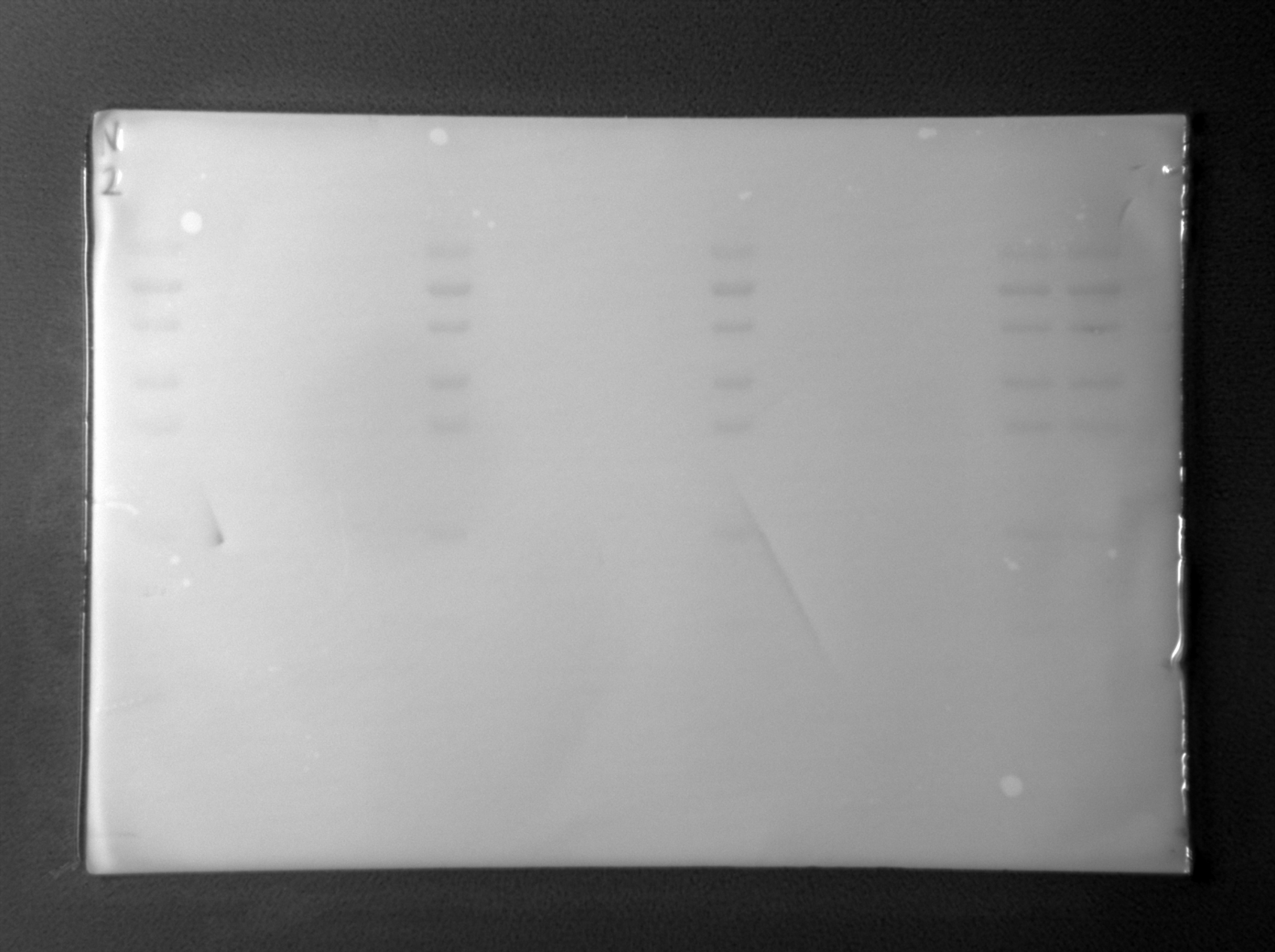

Supplement: Supplementary file 1 — Full and uncropped western blots [file 41419_2025_7809_MOESM1_ESM.zip › Full and uncropped western blots/Fig5I-co-ip/IP-NEDD4L/IB-SLC7A11/picture of film 22Rv1.tif]

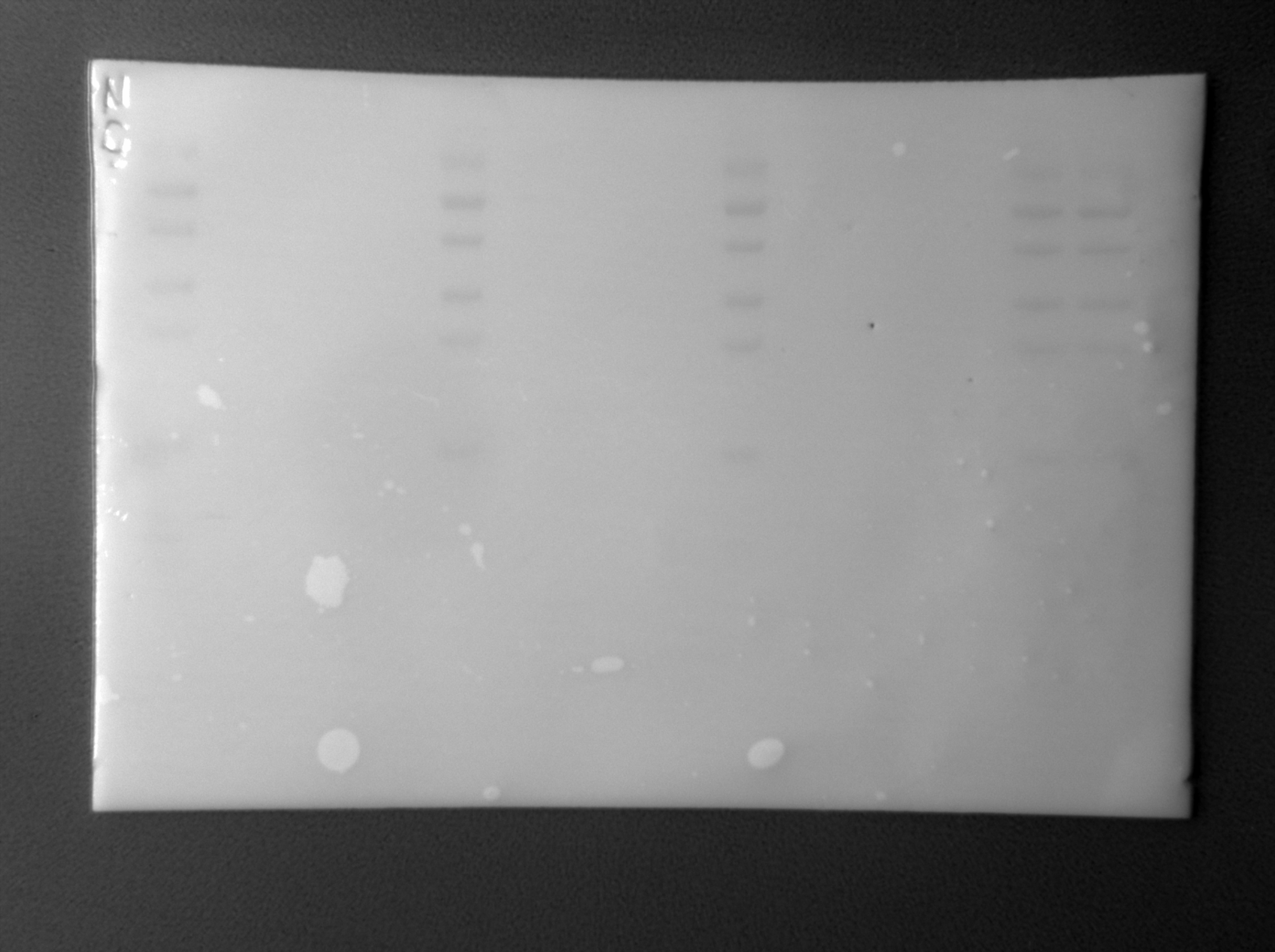

Supplement: Supplementary file 1 — Full and uncropped western blots [file 41419_2025_7809_MOESM1_ESM.zip › Full and uncropped western blots/Fig5I-co-ip/IP-NEDD4L/IB-SLC7A11/picture of film C42.tif]

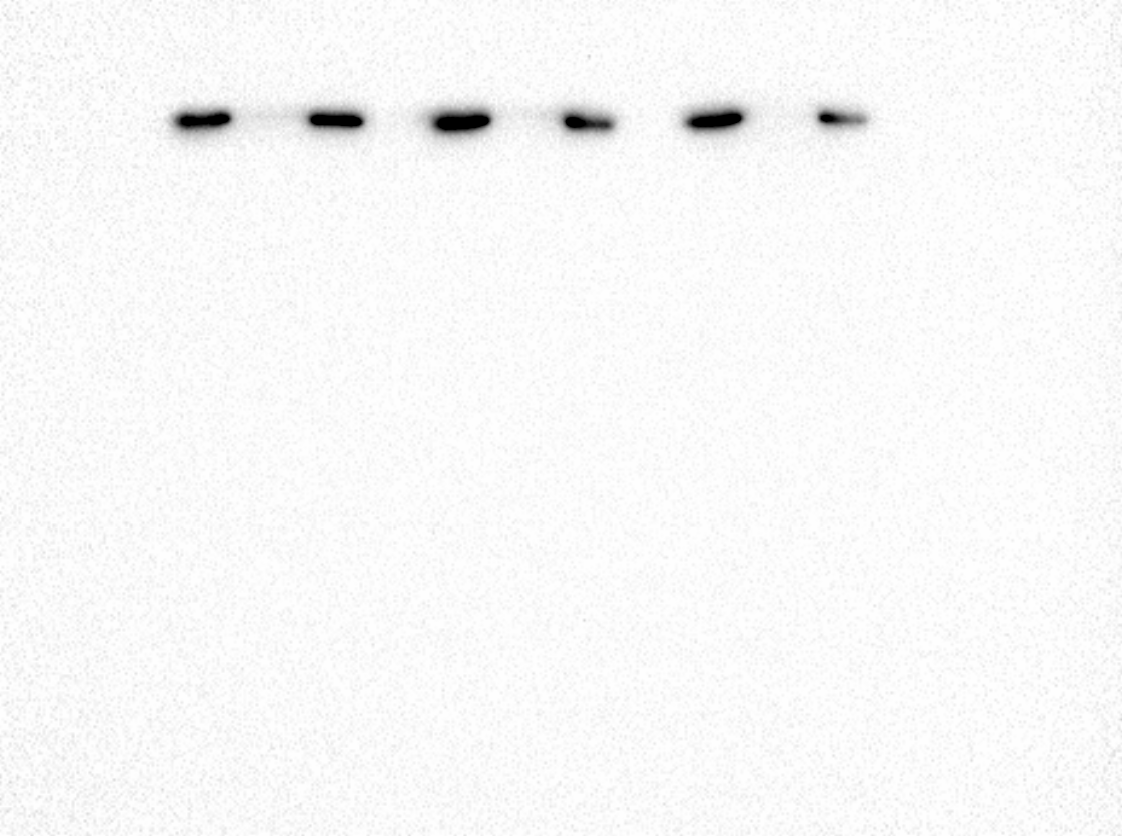

Supplement: Supplementary file 1 — Full and uncropped western blots [file 41419_2025_7809_MOESM1_ESM.zip › Full and uncropped western blots/Fig5I-co-ip/IP-SLC7A11/IB-NEDD4L/IP SLC7A11+IB NEDD4L 22Rv1.tif]

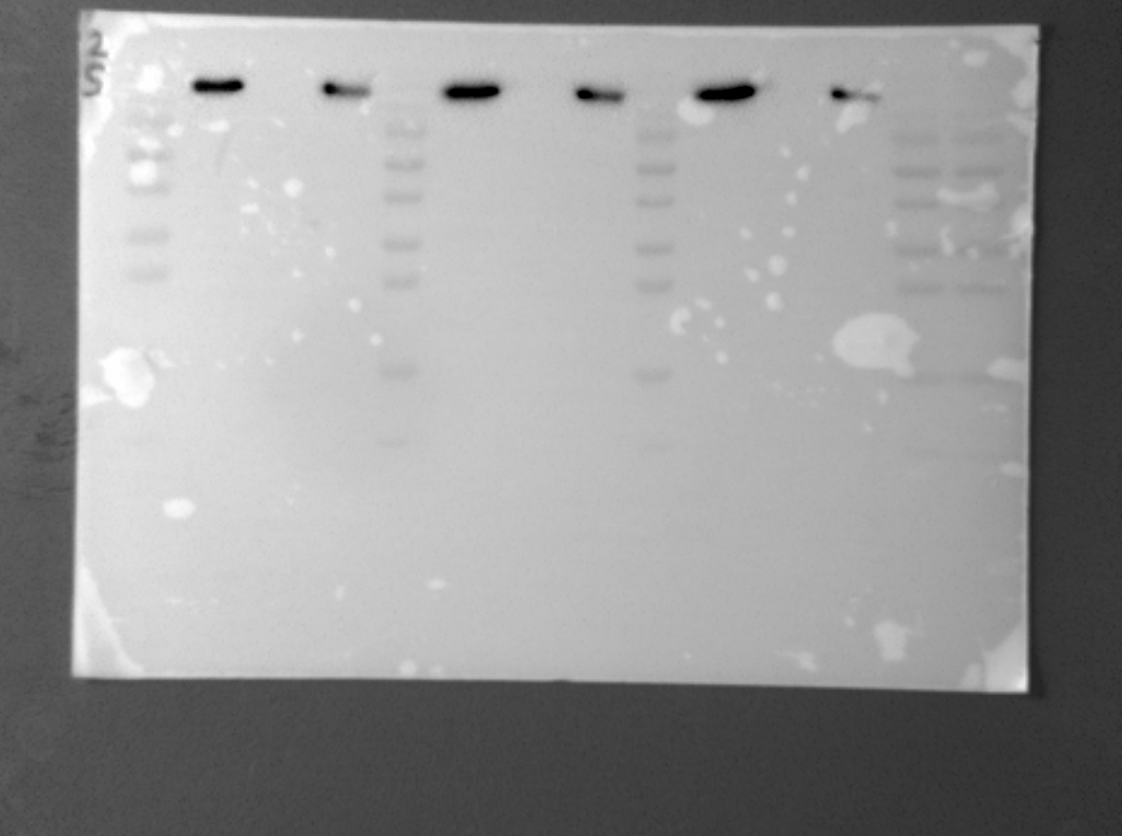

Supplement: Supplementary file 1 — Full and uncropped western blots [file 41419_2025_7809_MOESM1_ESM.zip › Full and uncropped western blots/Fig5I-co-ip/IP-SLC7A11/IB-NEDD4L/Merge 22RV1.tif]
